# Ex situ conservation of two rare oak species using microsatellite and SNP markers

**DOI:** 10.1111/eva.13650

**Published:** 2024-03-22

**Authors:** Austin C. Koontz, Emily K. Schumacher, Emma S. Spence, Sean M. Hoban

**Affiliations:** ^1^ Morton Arboretum Center for Tree Science Lisle Illinois USA; ^2^ Cornell University Department of Public and Ecosystem Health Ithaca New York USA

**Keywords:** bioinfomatics/phyloinfomatics, captive populations, conservation biology, conservation genetics, population genetics—empirical

## Abstract

Plant collections held by botanic gardens and arboreta are key components of ex situ conservation. Maintaining genetic diversity in such collections allows them to be used as resources for supplementing wild populations. However, most recommended minimum sample sizes for sufficient ex situ genetic diversity are based on microsatellite markers, and it remains unknown whether these sample sizes remain valid in light of more recently developed next‐generation sequencing (NGS) approaches. To address this knowledge gap, we examine how ex situ conservation status and sampling recommendations differ when derived from microsatellites and single nucleotide polymorphisms (SNPs) in garden and wild samples of two threatened oak species. For *Quercus acerifolia*, SNPs show lower ex situ representation of wild allelic diversity and slightly lower minimum sample size estimates than microsatellites, while results for each marker are largely similar for *Q. boyntonii*. The application of missing data filters tends to lead to higher ex situ representation, while the impact of different SNP calling approaches is dependent on the species being analyzed. Measures of population differentiation within species are broadly similar between markers, but larger numbers of SNP loci allow for greater resolution of population structure and clearer assignment of ex situ individuals to wild source populations. Our results offer guidance for future ex situ conservation assessments utilizing SNP data, such as the application of missing data filters and the usage of a reference genome, and illustrate that both microsatellites and SNPs remain viable options for botanic gardens and arboreta seeking to ensure the genetic diversity of their collections.

## 1 INTRODUCTION

Ex situ conservation is an important tool for safeguarding plant species, of which an estimated 20% are threatened with extinction (Antonelli et al., 2020). Botanic gardens and arboreta are key institutions in the practice of ex situ conservation, serving as living collections and facilitating research and education in plant sciences (Westwood et al., 2021). Crucially, such living collections can act not only as repositories of species diversity, but reservoirs of genetic diversity (Griffith et al., 2019), which is necessary for plant populations to adapt to threats such as diseases, pests, climate change, and natural catastrophes (Reusch et al., 2005; Reynolds et al., 2012). For collections to serve as genetic resources for plant species, wild (in situ) populations need to be sampled in such a way that sufficiently represents their allelic diversity and ensures the overall genetic diversity of garden (ex situ) collections. A standard established by Marshall & Brown (1975) maintains that sampling 50 individuals from every wild population will ensure the representation of approximately 95% of the alleles in those populations. However, this guideline fails to accommodate biological aspects of plant species, such as population structure (Hoban & Schlarbaum, 2014) and different life history strategies (Hoban, 2019).

Despite widespread recognition that genetic diversity is essential to population persistence, many ex situ collections fall below genetic conservation targets (Hoban et al., 2020). To counteract this deficiency, a body of research has developed more comprehensive sampling guidelines for building genetically diverse ex situ collections (Bragg et al., 2021), based on empirical studies (Griffith et al., 2017; Zumwalde et al., 2022) and simulations (Bragg et al., 2021; Rosenberger et al., 2022). A majority of these studies have used microsatellites as genetic markers for determining minimum sample sizes and measuring the genetic diversity (heterozygosity, allelic richness, etc.) of in situ and ex situ populations (Puckett, 2017; Wei & Jiang, 2020).

Because of their highly polymorphic nature, microsatellites can provide relatively high statistical power to distinguish between closely related individuals (Guichoux et al., 2011) for a moderate price compared to other markers (Flanagan & Jones, 2019), and their usage in population genetics remains well‐supported (Guichoux et al., 2011; Hauser et al., 2021; Hodel et al., 2016). However, the mutation processes which contribute to the highly polymorphic nature of microsatellites can also lead to homoplasy, confounding common population genetic inferences based on an infinite allele mutation model (Putman & Carbone, 2014). Moreover, the number of microsatellite loci used in a given study may be insufficient for resolving fine‐scale differences between individuals (Thrasher et al., 2018). Single nucleotide polymorphisms (SNPs), often generated by restriction site‐associated DNA sequencing (RADseq) techniques, have become a popular alternative to microsatellites (Andrews et al., 2016; Schlötterer, 2004), in part because these approaches generate thousands of loci without requiring prior genetic information. The advent of RADseq approaches has led to numerous publications comparing microsatellites and SNPs and the inferences drawn from each (Hauser et al., 2021; Lemopoulos et al., 2019; Thrasher et al., 2018).

Genetic marker comparisons have largely demonstrated that broad measures of population differentiation generally correspond between microsatellites and SNPs, but the increased resolution of SNPs often allows for a more nuanced interpretation of population demography (Osborne et al., 2022; Putman & Carbone, 2014; Thrasher et al., 2018). The large number of SNP loci generated by RADseq approaches also allow for detection of subtle genetic distinctions even when only a few individuals are sampled (Jeffries et al., 2016). However, the conservation implications of using SNPs remain uncertain, especially in the context of ex situ collections, and a reconsideration of sample size recommendations for genetic ex situ conservation remains unaddressed in the volume of publications comparing markers.

Differences in the mutation processes, number of alleles, and allele frequencies between marker types may result in different recommended sample guidelines for botanic gardens to conserve the majority of wild genetic variation. Botanic gardens are also often hampered by incomplete or dubious accession data (documentation of geographic and/or demographic origin of a collection; Goodwin et al., 2015), and even subtle alterations in the genetic assignment of garden samples to wild source populations could have notable conservation implications. An additional, practical concern is that the usage of either marker type implies taking into account different methodological considerations, which have the potential to impact genotyping results. For microsatellites, this includes ambiguities when scoring alleles and addressing the presence of null alleles (Choquet et al., 2023). For SNPs, decisions must be made regarding the approach used for SNP calling (de novo assembly or mapping sequences to a reference genome), which have been shown to impact downstream population genetic inferences (Cumer et al., 2021; Shafer et al., 2017). Additionally, the large number of loci afforded by SNP approaches often lead to higher levels of missing data (loci unshared with all individuals in a sample set), which can impact estimations of key parameters (Chattopadhyay et al., 2014; Gautier et al., 2013). This requires researchers to be more deliberate in their methodology for filtering loci, which can influence the level of differentiation observed between marker types (Hodel et al., 2017).

In light of a growing understanding of how population genetic analyses are affected by the decision to use microsatellite or SNP markers, and because most research informing the genetic sufficiency of ex situ collections is based on microsatellites, we sought to examine how both markers impact measures of ex situ conservation. To do so, we analyze two rare oak species—*Quercus acerifolia* and *Q. boyntonii*—endemic to the southeastern United States, with narrow distributions in the wild but global representation in ex situ collections. We perform a novel comparison of ex situ representation measures generated using microsatellite and SNP data for each species. The aims of this study are as follows:

1. Determine if different genetic markers (microsatellites versus SNPs) impact measures of ex situ representation, and how those differences change conservation recommendations for botanic gardens (e.g., minimum sample sizes).
2. Determine if the approach used for processing SNP loci (de novo assembly versus reference alignment and different filters for missing data) modifies estimates of ex situ representation and sampling recommendations provided to botanic gardens.
3. Compare population clustering using both microsatellites and SNPs to determine whether marker effects on population assignment influence the representation of wild populations ex situ.

To our knowledge, this study is the first to measure ex situ conservation using both of these widely used genetic markers, and thus provides a valuable opportunity for guiding future ex situ conservation of genetic diversity.

## 2 MATERIALS AND METHODS

### 2.1 Study species

To examine how microsatellite and SNP markers impact measures of ex situ conservation, we studied two IUCN Red List Endangered Oak species endemic to the southeastern United States: *Quercus acerifolia* and *Quercus boyntonii*.

*Quercus acerifolia* (Maple‐leaved oak; Palmer, Stoynoff & Hess; Fagaceae) is a red oak (section *Lobatae*) which grows as a large shrub or a small tree (up to 9 m tall) in early successional woodland areas, with a preference for xeric to mesic sites with rocky outcrops and steep slopes. Its distribution consists of four (possibly five) disjunct populations located on mountaintops of west‐central Arkansas, largely within the Ouachita National Forest (Jerome et al., 2017). It is categorized as Endangered by the International Union for the Conservation of Nature (IUCN) Red List, with human landscape use identified as the top threat to its narrow distribution (Beckman et al., 2019).

*Q. boyntonii* (Alabama sandstone post oak; Beadle; Fagaceae) is a white oak (section *Quercus*) which grows as a large shrub or a small tree (up to 6 m in height). It has a patchy distribution across several counties in Alabama, occurring on sandstone outcrops of pine‐oak‐hickory forests (Beckman et al., 2019). It is categorized as Critically Endangered, but with a stable population, on the IUCN Red List and is threatened by encroaching human development (Carrero et al., 2020).

Figure S1 provides a map of the sampling locations for wild *Q. acerifolia* and *Q. boyntonii* individuals.

### 2.2 Sampling

Sampling for *Q. acerifolia* and *Q. boyntonii* individuals is briefly described here, but more information can be found in Schumacher et al. (2023) for *Q. acerifolia*, and Hoban et al. (2020) and Spence et al. (2021) for *Q. boyntonii*.

Leaves of 174 wild individuals from all of *Q. acerifolia*'s wild populations—Porter Mountain, Magazine Mountain, Pryor Mountain, Sugar Loaf Mountain, and Kessler Mountain Regional Park—were collected in 2019 and shipped to the Morton Arboretum on silica for processing. Leaf tissue from 289 *Q. acerifolia* garden samples were received from 17 different institutions in 2019. For *Q. boyntonii*, leaf tissue from 246 wild individuals were sampled in May 2017 from 4 counties in Alabama; tissue from 86 garden individuals from 15 different institutions was shipped to the Morton Arboretum on ice and stored at –80°C upon arrival between April and September 2017.

A subset of samples was selected for NextRAD sequencing on four 96‐well plates. Wild samples of each species were selected to evenly represent all populations and maximize the geographic distances between individuals within populations, to minimize the likelihood of sequencing related individuals. For *Q. acerifolia*, ex situ individuals were selected to represent all gardens and as many accessions, or maternal lines, as possible; for *Q. boyntonii*, all 86 ex situ individuals were sequenced. Six duplicate samples (four *Q. acerifolia* garden, one *Q. acerifolia* wild, and one *Q. boyntonii* wild) were included to assess potential plate‐based impacts on sequencing quality and the extent of inflated polymorphism due to sequencing error.

Ultimately, 198 *Q. acerifolia* individuals (100 garden, 98 wild) were sequenced for SNPs, 441 samples were sequenced for microsatellites, and 179 samples were sequenced using both markers (Table S1). For *Q. boyntonii*, 183 individuals (86 garden, 97 wild) were sequenced for SNPs, 322 samples were sequenced for microsatellites, and 171 samples were sequenced using both markers (Table S2).

### 2.3 Library preparation and sequencing

DNA was extracted following a protocol described in Zumwalde et al. (2022). Details for microsatellite genotyping can be found in the Data S1: Section 1.4.1. For SNPs, after quality checks and standardization, samples were shipped to SNPsaurus LLC for final library preparation and sequencing using the NextRAD genotyping approach (Russello et al., 2015). All 381 libraries were sequenced on a NovaSeq 6000 S4 lane to generate 150 bp paired‐end reads. Genome sizes for each species are estimated to be ~750 Mb, similar to the sizes of reference genomes used (Plomion et al., 2018 for *Q. robur*; Kapoor et al., 2023 for *Q. rubra*; see below). More genotyping details for SNPs can be found in the Data S1: Section 1.4.2.

### 2.4 Bioinformatic analyses

Bioinformatic analyses were run using Stacks2 (v2.59; Catchen et al., 2013) and packages in the R environment (v4.2.2; R Core Team, 2022). Commands and parameters from the Stacks2 software are presented in block text; R packages are presented in *italics*. Other software used in the analysis are cited accordingly.

#### 2.4.1 Quality filtering

Raw demultiplexed reads were passed through the FastQC (Andrews, 2010) and Stacks software to assess sequencing quality. Specifically, the Stacks process_radtags command was used to remove adapter sequences, drop low quality bases, and drop reads below 100 bp in length. Due to low numbers of reads, two *Q. boyntonii* samples were removed from all downstream analyses. Further information on quality filtering is available in the Supporting Information.

#### 2.4.2 Genotyping

To determine how different approaches for processing SNP loci modify measures of ex situ conservation, we analyzed SNP datasets using two different techniques (Rochette & Catchen, 2017). First, we generated optimized de novo assemblies following the method described in Paris et al. (2017). Second, we built reference alignments for both species, and passed these through Stacks. We describe the general steps for each approach below, as well as in the Supporting Information; details for microsatellite genotyping can be found in the Data S1: Section 1.4.1.

##### De novo assembly

We generated optimized de novo assemblies following Paris et al. (2017). For sample subsets in each species, loci were assembled using the Stacks denovo_map.pl script with different values of various parameters (Table S7), and metrics were calculated for each assembly (Figures [Link], [Link], [Link], [Link], [Link], [Link]). The parameter values which maximized assembly metrics (Table S8) were then used to build a de novo assembly for all samples of that species.

##### Reference alignment

To generate reference alignment datasets, quality‐filtered sequences were aligned using GSNAP (Wu & Nacu, 2010) to different reference genomes based on the section of the *Quercus* genus each sample species was found in. *Q. acerifolia* (section *Lobatae*, *Quercus* subgenus) samples were aligned to the red oak (*Q. rubra*) reference genome (Kapoor et al., 2023). *Q. boyntonii* (section *Quercus*, *Quercus* subgenus) samples were aligned to the English oak (*Q. robur*) reference genome (V2_2N, Plomion et al., 2018). Because the two study species are separated by over 50 million years of evolution (Hipp et al., 2020), we chose to utilize two different reference genomes to optimize the reference alignment for each species separately. Reference alignments were then passed through the Stacks ref_map.pl script to generate loci datasets.

#### 2.4.3 SNP filtering and population assignment

After generating loci using each approach described above, results were passed to the Stacks populations module to apply filters to loci and alleles, group garden and wild samples into separate populations, and generate outputs for downstream analyses. Following recommendations from Hodel et al. (2017) on analyzing genomic data using multiple missing data filters, we utilized two values for the ‐R parameter, which specifies the minimum percentage of individuals in which a locus must be present to be included in the dataset. For both de novo and reference approaches, data were generated using R0 (no filter on missing data) and R80 (loci present in less than 80% of all samples are dropped). While the absence of a missing data filter (R0) is uncommon for many SNP analyses, allowing for higher levels of missing data can reveal informative patterns in the relations between populations (Crotti et al., 2019; Huang & Knowles, 2016). We therefore chose to examine SNP datasets with and without missing data filters, to assess the impacts of missing data on assessments of ex situ conservation.

To ease downstream computation, account for linkage between proximate SNP loci, and ensure replicability of our ex situ conservation analyses, we used the populations module to write only the first SNP per locus. To avoid the possible removal of loci valuable for our ex situ conservation analyses (especially within garden populations of samples), we chose not to utilize a filter for Hardy–Weinberg equilibrium. Additionally, we found in preliminary analyses that specification of a minimum minor allele frequency threshold (using the ‐‐min‐maf argument in the Stacks populations module) inflated levels of ex situ representation of rare alleles (<1% frequency in the wild population; see below) to 100% representation. Rare alleles are of particular interest in the context of ex situ conservation, as botanic garden collections often seek to safeguard unique genetic polymorphisms that may be lost in wild populations. Therefore, we chose to conduct our analyses without the specification of minor allele frequency threshold, to avoid potentially biasing our measures of ex situ representation of rare alleles. Finally, recent analyses have demonstrated that the Kessler Mountain population of *Q. acerifolia* is partly composed of admixed individuals likely resulting from crosses between *Q. acerifolia* and *Q. shumardii* (Wu et al., 2023). The presence of hybrid individuals could have confounding impacts on population clustering analyses (Vähä & Primmer, 2006); moreover, hybrid individuals are likely to harbor alleles from parental species not included in this study, which would greatly distort any ex situ representation results. For these reasons, we chose to remove these 6 wild individuals from our datasets.

### 2.5 Ex situ conservation analyses

For each combination of markers and SNP calling approaches (microsatellite and SNP datasets; for SNPs, R0 and R80 loci from de novo assemblies and reference alignments) we calculated two ex situ conservation measures. First, we measured the ex situ representation of wild alleles at different frequencies as a percentage, which indicates how well wild genetic diversity is maintained in botanic gardens and arboreta. Second, we estimated the minimum sampling size required to represent 95% of the total wild allelic diversity, a measure meant to represent the sampling “efficiency” of an ex situ collection.

For each of these measures, we calculated values for all the samples included in the microsatellite and SNP studies (“Complete” datasets). Additionally, to control for the impact of different sample sizes between marker studies, we subset Complete datasets to only include samples shared between microsatellite and SNP studies (“Subset” datasets). For *Q. acerifolia*, 179 samples (88 garden, 91 wild) were shared between marker datasets; for *Q. boyntonii*, 169 samples (75 garden, 94 wild) were shared between marker datasets. Analyses using Subset datasets allowed us to generate a more direct comparison between markers. Table S10 outlines the usage of garden and wild samples for each loci dataset.

#### 2.5.1 Ex situ representation

We analyzed how many wild alleles were observed at least once in garden samples by processing the genind objects generated by the Stacks populations module with R the package *adegenet* v2.1.5 (Jombart, 2008; Jombart & Ahmed, 2011). We generated percentages of ex situ representation for all wild alleles (“Total”), as well for alleles of four different frequency categories (as described in Hoban et al., 2020: “Very common,” >10%; “Common,” greater than 5%; “Low frequency,” between 1% and 10%; “Rare,” <1%) for both Complete and Subset microsatellite and SNP datasets.

#### 2.5.2 Resampling analyses

Similar to Richards et al. (2007) and Hoban et al. (2020), we generated estimates of the minimum sampling size required to reach 95% of the total allelic diversity represented in wild samples. To do so, we randomly subsampled the total number of wild individuals of each species and measured the allelic diversity represented in that subsample. We did this for every subsample size between 2 and the total number of wild samples, and determined the subsample size required to represent 95% of the total allelic diversity. We iterated this process 5000 times, and calculated the mean and standard deviation of the 95% minimum sampling size estimate across replicates. We performed these calculations for every loci dataset (microsatellites, SNP de novo R0, SNP de novo R80, SNP Reference R0, SNP Reference R80) using both Complete and Subset datasets (see Table S10).

### 2.6 Population analyses

#### 2.6.1 Population genetic statistics

For each species, the following values were calculated: expected heterozygosity (*H*_e_) and total allele counts, using the *adegenet* package v.2.1.5; allelic richness (*A*_R_) and Nei's *F*_ST_ (mean pairwise values), using the R package *hierfstat* v0.5‐10 (Goudet & Jombart, 2021). Mean pairwise *F*_ST_ values were calculated using only wild samples, by grouping wild samples into populations based on previous studies (for *Q. acerifolia*, see Jerome et al., 2017; for *Q. boyntonii*, see Spence et al., 2021); all other metrics were calculated using garden and wild samples.

All values were calculated using both Complete and Subset datasets for both markers. In addition, for SNP datasets, values were calculated for each approach (de novo and Reference) and each level of missing data (R0 and R80).

#### 2.6.2 Population clustering analyses

We briefly describe the two methods we took to assess population structure across markers and SNP genotyping approach. More details for each method are available in the Supporting Information.

##### STRUCTURE

To examine the impact of genetic markers on population clustering, microsatellite and SNP datasets were processed using STRUCTURE (Pritchard et al., 2000). To directly compare results from different markers, we clustered only wild samples shared between microsatellite and SNP (R80) datasets (“Subset” samples). For each species, we assessed *K* values ranging from 2 to 7, and used CLUMPAK (Kopelman et al., 2015) to summarize results across replicate STRUCTURE runs and generate CLUMPP files (Jakobsson & Rosenberg, 2007), which were used for visualization.

###### Garden provenance assessments.

In addition to assessing differences in wild population clustering across markers, we sought to examine how different markers impacted STRUCTURE's ability to assign garden samples back to wild source populations, as incorrect provenance data is known to be an issue in many ex situ collections (Diaz‐Martin et al., 2023). To do so, we analyzed Subset garden and wild samples using microsatellite and SNP (de novo and Reference) markers. Because *Q. acerifolia* has stronger wild population structure (Wu et al., 2023) than *Q. boyntonii* (Spence et al., 2021), we focused solely on *Q. acerifolia* for this analysis.

##### Discriminant Analysis of Principal Components

To complement our STRUCTURE results, we also examined population clustering using Discriminant Analysis of Principal Components (DAPC) in the *adegenet* R package (Jombart et al., 2010). DAPC first transforms SNP data using a traditional principal component analysis (PCA) and then utilizes k‐means clustering to generate likelihoods corresponding to each number of clusters. Bayesian Information Criterion (BIC) scores are calculated for each number of clusters, allowing for the best supported number of clusters to be assessed. In order to make our results across clustering analyses as comparable as possible, we used the same datasets as those for our STRUCTURE analyses: only wild samples in Subset datasets, with R80 loci used for SNP datasets.

## 3 RESULTS

### 3.1 Bioinformatic analyses

Sequencing generated 2,610,711,858 *Q. acerifolia* and 2,471,556,790 *Q. boyntonii* raw reads. De novo assembly metric values across m, M, n, and gt‐alpha parameters are shown in Figures [Link], [Link], [Link] for *Q. acerifolia*, and Figures [Link], [Link], [Link] for *Q. boyntonii*. Optimized de novo assembly values and reference alignment statistics for both species are listed in Tables S4 and S5, respectively. Mapping percentages to reference genomes were higher for *Q. acerifolia* than for *Q. boyntonii* samples, likely a result of *Q. acerifolia* being more closely related to *Q. rubra* than *Q. boyntonii* is to *Q. robur* (Hipp et al., 2020). Frequency spectra for wild alleles using microsatellite, SNP de novo (R80), and SNP reference alignment datasets (Subset) are shown in Figure S8 (*Q. acerifolia*) and Figure S9 (*Q. boyntonii*).

### 3.2 Ex situ conservation

#### 3.2.1 Ex situ representation

##### Marker comparison

For *Q. acerifolia*, microsatellites generated higher measures of ex situ representation than SNPs, for both Complete and Subset datasets (Table 1; see Table S11 for raw values). The difference between markers in overall ex situ representation is mostly driven by lower representation of low frequency and rare alleles.

**TABLE 1:** Ex situ representation values for *Quercus acerifolia* across marker types.

*Quercus acerifolia*: Ex situ representation						
	Data type (sample size)	Very common (>10%)	Common (>5%)	Low frequency (1%–10%)	Rare (<1%)	Total (>0%)
*Complete*	Microsatellites (277 Garden, 164 Wild)	100.00%	100.00%	98.53%	82.61%	96.24%
	SNPs: De novo, R0 (96 Garden, 91 Wild)	99.82%	97.97%	74.77%	42.26%	76.43%
	SNPs: De novo, R80 (96 Garden, 91 Wild)	100.00%	99.91%	73.28%	41.06%	84.96%
	SNPs: Reference, R0 (96 Garden, 91 Wild)	99.93%	98.73%	89.37%	51.02%	87.58%
	SNPs: Reference, R80 (96 Garden, 91 Wild)	100.00%	99.93%	67.01%	43.99%	83.40%
*Subset*	Microsatellites (88 Garden, 91 Wild)	100.00%	100.00%	92.00%	90.00%	94.40%
	SNPs: De novo, R0 (88 Garden, 91 Wild)	99.79%	97.72%	73.36%	40.71%	75.32%
	SNPs: De novo, R80 (88 Garden, 91 Wild)	99.98%	99.87%	72.27%	39.74%	84.50%
	SNPs: Reference, R0 (88 Garden, 91 Wild)	99.92%	98.56%	88.32%	49.44%	86.73%
	SNPs: Reference, R80 (88 Garden, 91 Wild)	100.00%	99.92%	65.85%	43.03%	82.98%

*Note*: Values represent allelic diversity represented in different categories of alleles based on their frequency in wild samples, as well as the sum across all frequency categories (“Total”). Rows indicate different marker types and processing approaches for SNP markers (de novo assembly and reference alignment). R values indicate the missing data level (R0: no filter on missing data; R80: loci present in at least 80% of all samples). “Complete” values indicate all samples for each marker type; “Subset” values indicate only samples shared across microsatellite and SNP datasets. Values in parentheses show the number of garden and wild samples for each dataset. Raw counts for alleles in each category can be found in Table S11.

For *Q. boyntonii*, measures of ex situ representation are more comparable, but SNP ex situ representation values are higher than microsatellite values in both Complete and Subset datasets (Table 2; see Table S12 for raw values). This difference is driven by representation rates of rare alleles, which (in Subset datasets) are represented in gardens at double the frequency than rare alleles represented for microsatellites. Ex situ representation for *Q. boyntonii* is consistently lower than *Q. acerifolia*.

**TABLE 2:** Ex situ representation values for *Quercus boyntonii* across marker types.

*Quercus boyntonii*: Ex situ representation						
	Data type (sample size)	Very common (>10%)	Common (>5%)	Low frequency (1%–10%)	Rare (<1%)	Total (>0%)
*Complete*	Microsatellites (77 Garden, 245 Wild)	100.00%	100.00%	66.20%	31.71%	60.31%
	SNPs: De novo, R0 (85 Garden, 95 Wild)	99.67%	96.94%	66.55%	36.24%	69.46%
	SNPs: De novo, R80 (85 Garden, 95 Wild)	99.98%	99.84%	66.44%	28.19%	77.87%
	SNPs: Reference, R0 (85 Garden, 95 Wild)	99.88%	98.21%	86.42%	44.59%	84.37%
	SNPs: Reference, R80 (85 Garden, 95 Wild)	100.00%	99.80%	59.71%	31.79%	76.34%
*Subset*	Microsatellites (75 Garden, 94 Wild)	100.00%	97.14%	69.12%	16.67%	66.04%
	SNPs: De novo, R0 (75 Garden, 94 Wild)	99.59%	96.43%	64.21%	34.02%	67.54%
	SNPs: De novo, R80 (75 Garden, 94 Wild)	99.98%	99.83%	63.99%	26.25%	76.90%
	SNPs: Reference, R0 (75 Garden, 94 Wild)	99.80%	97.79%	84.52%	42.35%	82.81%
	SNPs: Reference, R80 (75 Garden, 94 Wild)	100.00%	99.77%	56.73%	29.87%	75.36%

*Note*: See Table 1 caption for explanation. Raw counts for alleles in each category can be found in Table S12.

##### SNP methodological comparison

###### De novo assembly versus reference alignment

In both species, de novo datasets generate lower measures of ex situ representation than reference alignment datasets, and these differences are driven by lower levels of representation of low frequency and rare alleles.

###### Missing data

In de novo datasets for both species, moving from R0 to R80 increases measures of ex situ representation, despite lower percentages of rare alleles being represented in R80 datasets compared to R0. In reference alignment datasets for both species, moving to R80 decreases measures of ex situ representation, and this is driven by lower levels of representation for low frequency and rare alleles.

#### 3.2.2 Resampling

##### Marker comparison

For marker comparisons in both species, we utilized Subset datasets to compare estimates of the minimum sample size required to represent 95% of the total wild allelic diversity, as these estimates are impacted by the total number of wild samples included in the analysis (for example, compare the Complete and Subset *Q. boyntonii* microsatellites datasets in Figures S16 and S17).

**FIGURE 1:**
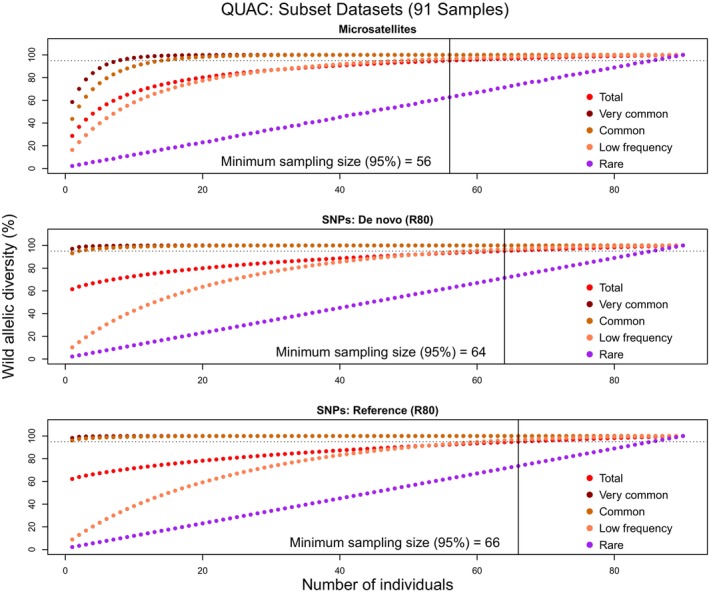
QUAC resampling plots. Allelic diversity percentages represented by increasing numbers of wild *Quercus acerifolia* individuals. Horizontal axes represent the number of wild individuals (Subset, shared between microsatellite and SNP studies); vertical axes represent the average percentage of alleles represented in those individuals. Different colors represent different allele frequency categories, with bright red being the Total level of allelic diversity. Horizontal dotted lines represent the 95% allelic diversity threshold taken as a standard for ex situ collections; solid vertical lines represent the average number of samples required to reach that 95% threshold. Top graph: microsatellite markers; middle graph: de novo (R80) SNP markers; bottom graph: reference‐aligned (R80) SNP markers.

For *Q. acerifolia*, the 95% minimum sample size is larger when using SNP markers (de novo: 64 samples; reference: 66 samples), compared to microsatellite estimates (56 samples; Figure 1; Figures [Link], [Link], [Link], [Link], [Link], [Link]; Table 3). Increasing missing data filters from R0 to R80 reduces SNP estimates slightly (for both approaches), but not to the level of microsatellite estimates. This corresponds with our ex situ representation findings for *Q. acerifolia*, which suggest slightly lower ex situ representation using SNPs versus microsatellites.

**TABLE 3:** Mean minimum sampling size estimates for representing 95% of total wild allelic diversity of *Quercus acerifolia* and *Quercus boyntonii*.

Mean 95% minimum sample size estimates				
	*Q. acerifolia*		*Q. boyntonii*	
	Complete	Subset	Complete	Subset
Microsatellite	94 ± 1.60 (164)	56 ± 1.57 (91)	181 ± 1.64 (245)	75 ± 1.81 (94)
SNP, De novo, R0	75 ± 0.41 (91)	75 ± 0.41 (91)	80 ± 0.41 (95)	80 ± 0.41 (94)
SNP, De novo, R80	64 ± 0.36 (91)	64 ± 0.35 (91)	73 ± 0.33 (95)	72 ± 0.33 (94)
SNP, Reference, R0	70 ± 0.39 (91)	70 ± 0.39 (91)	73 ± 0.41 (95)	73 ± 0.40 (94)
SNP, Reference, R80	66 ± 0.29 (91)	66 ± 0.30 (91)	75 ± 0.35 (95)	74 ± 0.36 (94)

*Note*: “Complete” values represent estimates made using all samples originally analyzed for each dataset; “Subset” values represent estimates made using only samples shared between microsatellite and SNP datasets. Values are based on 5000 resampling replicates. Values in parentheses are the number of wild samples in each dataset.

**FIGURE 2:**
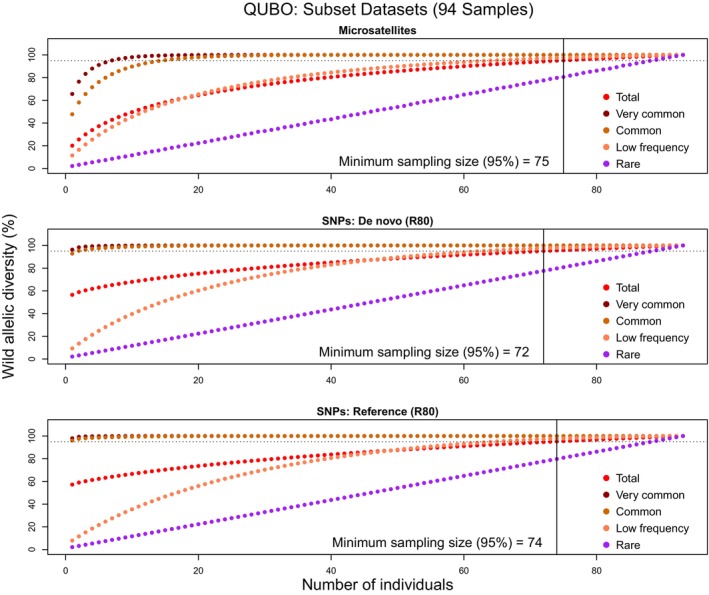
QUBO resampling plots. Allelic diversity percentages represented by increasing numbers of wild *Quercus boyntonii* individuals. Top graph: microsatellite markers; middle graph: de novo (R80) SNP markers; bottom graph: reference‐aligned (R80) SNP markers.

For *Q. boyntonii*, 95% minimum sample size estimates generated using SNPs (de novo: 72 samples; reference: 74 samples) are comparable to those generated using microsatellites (75 samples). SNP R80 values generated slightly lower minimum sample sizes estimates (Figure 2), and only R0 de novo datasets generated larger estimates than microsatellites (Figures [Link], [Link], [Link], [Link], [Link], [Link]; Table 3). Again, resampling results support our findings regarding overall ex situ representation in this species. In both species, minimum sample size estimates are more precise (lower standard deviations) using SNPs compared to microsatellites (Table 3).

##### SNP methodological comparison

Comparisons of minimum sample size estimates between de novo and reference alignment approaches depend on missing data filters. In both species, de novo R0 minimum sample size estimates are higher than reference alignment estimates (R0 and R80). In *Q. acerifolia*, de novo R80 minimum sample size estimates are lower than reference alignment estimates (R0 and R80). In *Q. boyntonii*, de novo R80 minimum sample size estimates are comparable to reference alignment estimates (R0 and R80).

### 3.3 Population differentiation and assignment

#### 3.3.1 Population genetic statistics

Population genetic statistics for *Q. acerifolia* and *Q. boyntonii* are shown in Tables 4 and 5, respectively. As expected, *H*_e_ and *A*_R_ values are lower using biallelic SNPs compared to multiallelic microsatellites, while differences in mean *F*_ST_ values across markers varies by species. Microsatellite datasets also show much greater variation in values of *A*_R_ and *H*_e_ between garden and wild samples; in SNPs, regardless of missing data filters or processing approach, values between garden and wild samples are almost identical. In SNPs, the reduction in loci caused by more stringent missing data filters (R80) tends to increase *A*_R_ values and greatly decreases *H*_e_ values; similar effects are seen between SNP processing approaches.

**TABLE 4:** *Quercus acerifolia* population genetic statistics samples using microsatellites and SNPs.

*Quercus acerifolia*: Population genetic statistics					
	Samples	Allele count	Allelic richness (*A*_R_)	Expected heterozygosity (*H*_e_)	Mean pairwise *F*_ST_ (Wild only)
Complete					
Microsatellites	Garden (277) Wild (164)	180	10.48 8.82	0.62 0.59	0.094
SNPs: De novo, R0	Garden (96) Wild (91)	254,886	1.28 1.28	0.28 0.27	0.24
SNPs: De novo, R80	Garden (96) Wild (91)	11,528	1.66 1.65	0.043 0.042	0.064
SNPs: Reference, R0	Garden (88) Wild (91)	234,230	1.33 1.33	0.30 0.29	0.12
SNPs: Reference, R80	Garden (88) Wild (91)	30,534	1.64 1.61	0.029 0.029	0.050
Subset					
Microsatellites	Garden (88) Wild (91)	163	10.29 8.28	0.63 0.60	0.089
SNPs: De novo, R0	Garden (88) Wild (91)	253,396	1.28 1.27	0.28 0.27	0.24
SNPs: De novo, R80	Garden (88) Wild (91)	11,501	1.65 1.63	0.043 0.042	0.064
SNPs: Reference, R0	Garden (88) Wild (91)	233,194	1.33 1.33	0.30 0.29	0.12
SNPs: Reference, R80	Garden (88) Wild (91)	30,425	1.60 1.58	0.030 0.029	0.050

*Note*: Complete and Subset values, as well as at two levels of missing data (R0 and R80), are shown. Only wild samples, grouped in populations according to previous research, are used for *F*_ST_ calculations.

**TABLE 5:** *Quercus boyntonii* population genetic statistics samples using microsatellites and SNPs.

*Quercus boyntonii*: Population genetic statistics					
	Samples	Allele count	Allelic richness (*A*_R_)	Expected heterozygosity (*H*_e_)	Mean pairwise *F*_ST_ (Wild only)
Complete					
Microsatellites	Garden (77) Wild (245)	134	11.65 9.01	0.65 0.59	0.046
SNPs: De novo, R0	Garden (85) Wild (95)	299,242	1.24 1.31	0.25 0.32	0.16
SNPs: De novo, R80	Garden (85) Wild (95)	10,566	1.53 1.73	0.038 0.043	0.072
SNPs: Reference, R0	Garden (85) Wild (95)	128,864	1.34 1.36	0.30 0.32	0.10
SNPs: Reference, R80	Garden (85) Wild (95)	12,496	1.50 1.68	0.025 0.029	0.058
Subset					
Microsatellites	Garden (75) Wild (94)	118	11.15 9.00	0.64 0.59	0.040
SNPs: De novo, R0	Garden (75) Wild (94)	295,977	1.23 1.30	0.24 0.31	0.16
SNPs: De novo, R80	Garden (75) Wild (94)	10,509	1.50 1.68	0.038 0.043	0.074
SNPs: Reference, R0	Garden (75) Wild (94)	127,939	1.34 1.36	0.30 0.30	0.10
SNPs: Reference, R80	Garden (75) Wild (94)	12,401	1.46 1.62	0.025 0.029	0.058

#### 3.3.2 Clustering results

##### STRUCTURE

**FIGURE 3:**
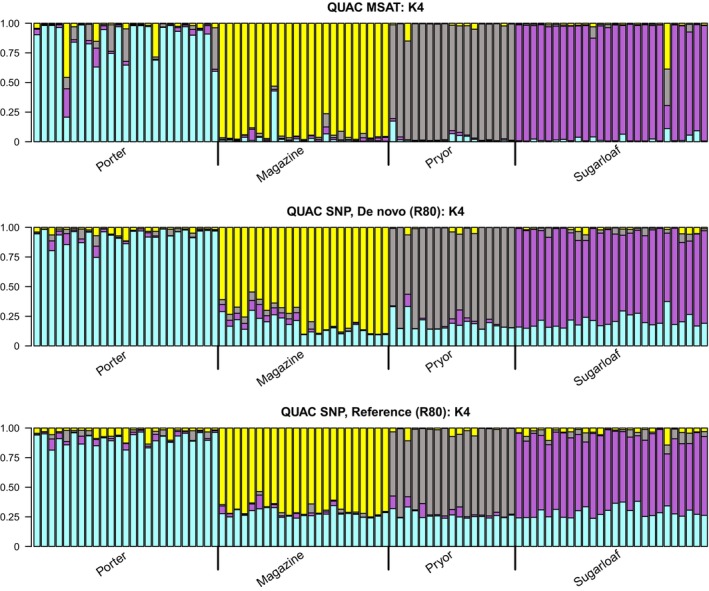
QUAC STRUCTURE plots. STRUCTURE plots across marker types for wild *Quercus acerifolia* individuals, at *K* = 4 clusters. Vertical axes represent Q‐matrix values, indicating percentage membership to different population clusters. Values shown are Major cluster results identified by CLUMPP across 50 replicate STRUCTURE runs. Geographic population assignments are indicated by text along the bottom of the graph. Top graph is using microsatellite markers; middle graph is using SNP markers from de novo datasets (R80); bottom graph is using SNP markers from reference‐aligned datasets (R80).

**FIGURE 4:**
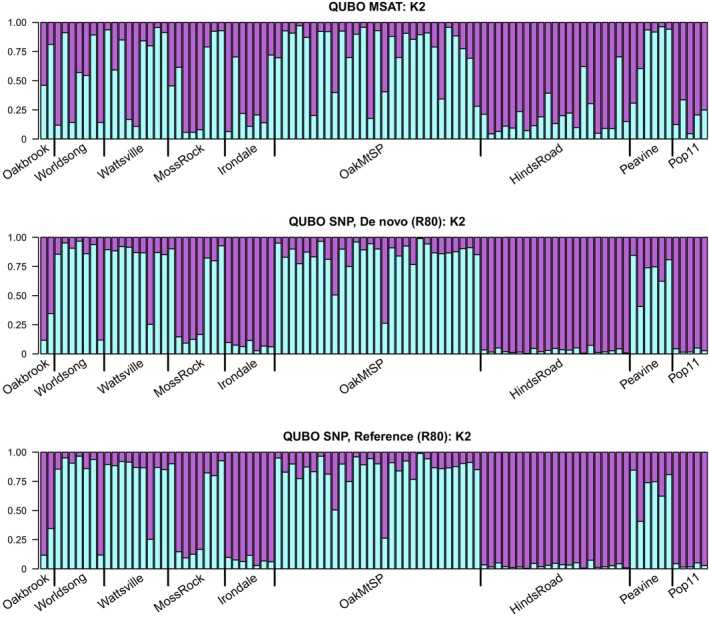
QUBO STRUCTURE plots. STRUCTURE plots across marker types for wild *Quercus boyntonii* individuals, at *K* = 2 clusters. Vertical axes represent Q‐matrix values, indicating percentage membership to different population clusters. Values are Major cluster results identified by CLUMPP across 50 replicate STRUCTURE runs. Site collection names, as outlined in Hoban et al. (2020) Supporting Information, are indicated along the bottom. R80 loci are used for SNP datasets (De novo and Reference).

Overall, STRUCTURE results correspond across marker types and approaches for both species (Figures S22 and S23), with similar assignment patterns across datasets. *Q. acerifolia* clusters largely coincide to the mountaintops on which the species is found (Figure 3). For *Q. boyntonii*, clustering is less determined by geography, illustrative of the more continuous distribution of this species (Figure 4).

While clustering patterns correspond across markers and approaches, there is no unambiguous agreement in optimal *K* values for either species (Table S13). At higher *K* values, microsatellite STRUCTURE assignments suggest a greater admixture within individuals; for SNP datasets, higher *K* values largely reiterate divisions observed at lower *K* values, especially in *Q. boyntonii*.

###### Garden provenance assessments

As in marker comparisons for both species, *Q. acerifolia* garden and wild samples cluster similarly across marker types, patterns in wild samples are strongly conserved, and there are minimal differences in results between SNP processing approaches (Figure S24). However, of the 83 garden samples with recorded wild provenances, 10 (12.04%) had differential assignments to wild populations between microsatellite and SNP clustering results. Additionally, eight garden samples appear to be misidentified (genetic clustering indicates an origin population different from garden records) across all marker datasets, with microsatellites and SNPs largely indicating similar wild source populations (although not always).

###### Discriminant Analysis of Principal Components

Similar to STRUCTURE results, DAPC reveals broad correspondence across datasets for both *Q. acerifolia* (Figures [Link], [Link], [Link]) and *Q. boyntonii* (Figures [Link], [Link], [Link]), but the optimal number of clusters (lowest BIC score) differs across markers. For *Q. acerifolia*, microsatellite datasets suggest an optimal number of clusters of *K* = 4, while for *Q. boyntonii*, microsatellite datasets suggest an optimal cluster value of *K* = 3. For SNP datasets (both de novo and reference) in both species, the optimal number of clusters is *K* = 2.

## 4 DISCUSSION

### 4.1 Overview

To guide botanic gardens and arboreta in the conservation of genetic diversity, we compared measures of ex situ conservation, as well as standard population genetic analyses and the assignment of garden plants back to wild populations, using microsatellites and SNPs. Marker choice impacts ex situ representation of certain classes of alleles and minimum sample size estimates, but to different extents across species. SNP methodological approaches also impact ex situ representation, and SNP data provide greater resolution of population structure, aiding gardens in identifying wild sources of garden plants. We consider the implications of how our findings fit into a larger body of work contrasting inferences drawn from these markers, and the impacts of our results on ex situ conservation of threatened species.

### 4.2 Ex situ conservation

#### 4.2.1 Marker comparison

The primary aim of our study was to compare ex situ representation across marker types. Our data demonstrate that representation of low frequency (present in 1%–10% of the wild population) and rare alleles (present in <1% of the wild population) is considerably different between microsatellites and SNPs. This is most notable in *Q. acerifolia*, where representation of these alleles is noticeably higher using microsatellites compared to SNPs (in both Complete and Subset datasets). In *Q. boyntonii*, representation rates are more comparable across markers, especially using the Subset dataset, and as with *Q. acerifolia*, the greatest difference between microsatellite and SNP results are in rare alleles. However, contrary to our expectations and our findings in *Q. acerifolia*, representation of rare alleles is higher using SNPs versus microsatellites in *Q. boyntonii*. This may be a result of sampling variance: in microsatellites, there are orders of magnitude fewer low frequency and rare alleles than in SNPs, so the presence or absence of a single allele in garden samples can cause the percentage of microsatellite alleles conserved ex situ to vary more widely (Tables 1 and 2).

Our findings on the ex situ representation of *Q. acerifolia* and *Q. boyntonii* strongly coincide with the results of our resampling analyses for determining minimum sample size estimates, which reflect the “efficiency” of an ex situ collection. For *Q. acerifolia* Subset datasets, microsatellite data indicate that fewer samples (56) are required to represent 95% of the total wild allelic diversity compared to R80 SNP datasets (de novo: 64 samples; reference: 66 samples). Meanwhile, resampling results are roughly equivalent across markers and approaches in *Q. boyntonii*. In both species, minimum sample size estimate variance is moderately decreased in SNP datasets, which results from more SNP loci allowing for more precise estimates of allelic diversity, but this effect is not so strong (differences of 1–2 samples) as to very meaningfully impact ex situ collections.

#### 4.2.2 SNP methodological comparison

Aim 2 of our study sought to explore the differences in ex situ representation based on methodologies used to process SNP data, to guide future studies utilizing SNPs to assess ex situ representation. In both species, there was an interaction between missing data filters and the approach used to generate SNP assemblies (de novo or reference): at R0, de novo approaches generated lower representation rates compared to R0 reference datasets, whereas at R80, representation values were more similar between approaches (Tables 1 and 2). Our resampling analyses also reflect this pattern: minimum sample size estimates are lower for R80 de novo datasets as compared to R0 de novo datasets, for both species.

This finding illustrates the impact of using reference alignments for generating SNP datasets. In de novo assemblies, it is possible for random, sample‐specific sequencing error to be interpreted as genuine polymorphism, which is reflected by higher numbers of loci for de novo datasets generally (Shafer et al., 2017). This is true even after parameter optimization, which (for the Stacks software) includes finding an optimal value for the parameter m, the minimum depth of coverage required to create a “stack” (putative allele). Because these alleles are unlikely to be shared with other samples, their representation in a sample subset (in this case, garden individuals) is lower. Applying a missing data filter mitigates this effect by pruning loci largely unshared with other samples from the dataset. Similarly, a minor allele frequency filter (−‐min‐maf, in the Stacks populations module) would mitigate the impact of erroneously identified loci in a de novo assembly; however, we intentionally chose to not specify a minor allele frequency, to avoid the biasing effect this parameter had on measures of rare allele ex situ representation.

In reference datasets, the impact of random sequencing error being misinterpreted as genuine polymorphism is moderated by the sequence alignment step. During alignment, the reference genome acts as a filter on uncommon polymorphisms present in the study species but unshared with the reference organism (Rivera‐Colón & Catchen, 2022). The extent to which reference alignment will filter loci depends both on the parameters used for alignment (such as the allowed number of mismatches, indel penalties, etc.) and on the phylogenetic distance between the reference organism and the study species, which is a proxy for the number of nucleotide differences between genomes. The impact of distance from the reference genome is observable in our results: *Q. acerifolia* is more closely related to *Q. rubra* than *Q. boyntonii* is to *Q. robur*, and as a result, *Q. acerifolia* reference datasets generate more loci than *Q. boyntonii* reference datasets, and ex situ representation levels in *Q. acerifolia* is higher. This comparison between study species highlights the value of having access to a reference genome of a closely related species.

As in de novo assemblies, the application of a missing data filter appears to mitigate the effect of reference genome filtering on ex situ representation values. In both species, the difference between de novo and reference approaches is lowered as missing data filters are applied. Thus, for future assessments of ex situ conservation using SNP data, the application of a missing data filter will likely increase the reproducibility of ex situ representation estimates, and is highly recommended. However, because these filters will also prune rare polymorphisms, it's important to consider ex situ representation levels using multiple data filter values (Hodel et al., 2017). Given the increase in genotyping accuracy offered by reference alignment approaches over de novo assemblies (Shafer et al., 2017), we also recommend that future SNP‐based ex situ conservation analyses utilize a reference approach, ideally of a closely related species (although a distantly related reference organism may suffice in some situations; see Galla et al., 2018). If no reference genome is available, then it is essential to optimize the parameters used for de novo assembly (for the Stacks software, see Paris et al., 2017). Finally, including duplicate samples during genotyping can allow researchers to assess (roughly) the extent to which random sequencing error is being misinterpreted as genuine polymorphism (Bresadola et al., 2020), and is highly recommended regardless of approach (de novo or reference).

### 4.3 Population differentiation and assignment

Aim 3 of our study sought to examine the impact of marker choice on population genetic statistics and clustering analyses. Our comparisons of *H*_e_, *A*_R_, and *F*_ST_ values across markers strongly coincide with prior studies (DeFaveri et al., 2013; Fischer et al., 2017; Zimmerman et al., 2020): relative patterns are similar, but absolute values of each statistic differ according to theoretical predictions. *H*_E_ and *A*_R_ values are larger using multiallelic microsatellites, while the biallelic nature of SNPs limits the possible ranges of these metrics. Measures of *F*_ST_ between microsatellites and R80 SNPs are comparable, coinciding with past findings of population divergence estimates using these marker types (Fischer et al., 2017; Muñoz et al., 2017). Similar to previous studies using simulated (Arnold et al., 2013; Gautier et al., 2013) and empirical data (Hodel et al., 2017), higher levels of missing data in SNPs (R0 datasets) increase heterozygosity and *F*_ST_ estimates by orders of magnitude. This results from low numbers of individuals having genotype data for many different loci, thereby falsely inflating the apparent genetic difference between populations. While these findings further emphasize the importance of utilizing a missing data filter (in addition to those mentioned above), evaluating datasets with higher levels of missing data can allow for sites with high levels of polymorphism to be retained in analyses (Crotti et al., 2019; Hodel et al., 2017; Huang & Knowles, 2016). Because such sites may reveal informative phylogenetic patterns that are hidden with more stringent missing data filters, the utilization of multiple missing data filter levels allows for a more complete and nuanced understanding of the genetic relations between individuals (Hodel et al., 2017).

In both study species, STRUCTURE and DAPC findings similarly follow trends established from past research. Broad population clustering patterns are shared across markers, but optimal *K* values (indicative of the number of “true” source populations) differ. Similar to Camacho‐Sanchez et al. (2020), we see higher admixture in microsatellite STRUCTURE results at higher *K* values, while SNP STRUCTURE results are reliably consistent across *K* values. For DAPC analyses, groups inferred from DAPC using SNP markers are more tightly clustered than those built using microsatellites, as in prior studies (Jeffries et al., 2016; Lemopoulos et al., 2019). Generally, results support the conclusion that larger numbers of SNP loci provide greater power to resolve finer‐scale spatial resolution in the genetic differences between individuals (Putman & Carbone, 2014; Thrasher et al., 2018; Osborne et al., 2022). This is particularly noticeable in STRUCTURE analysis of garden and wild *Q. acerifolia* individuals, where 10 garden samples are erroneously clustered using microsatellites. This finding illustrates that, despite increased costs and complexities, SNP approaches can be valuable for botanic gardens and arboreta, which are often beset by incorrect accession data (Goodwin et al., 2015; Diaz‐Martin et al., 2023).

Finally, our third aim sought to understand how SNP analyses build upon or refute past findings derived from microsatellites, to serve ongoing ex situ conservation efforts of these rare species. For *Q. acerifolia*, our data resolves similar population clusters seen in prior analyses, with individuals clustering according to mountaintop, and Magazine Mt. individuals splitting into 2 distinct groups at higher *K* values. For *Q. boyntonii*, our results support past findings of relatively low wild genetic diversity (Spence et al., 2021), and our SNP STRUCTURE analyses demonstrate a consistent bimodal clustering pattern even above values of *K* = 2. Further work needs to assess whether this bimodal pattern is caused by geographic separation, and what implications this may have for the ex situ conservation of this species.

### 4.4 Recommendations, caveats, and future directions

The primary goal of this study is to analyze how measures of ex situ conservation are impacted by genetic marker choice. As such, we provide recommendations for future ex situ conservation efforts of our study species and for practitioners seeking to develop genetically diverse botanic gardens and arboreta living collections. Our research indicates that ex situ representation of *Q. boyntonii* is stronger than what has been indicated using microsatellites (Spence et al., 2021). As this oak has a small geographic range (Beckman et al., 2019), this finding is encouraging and indicates that sufficient ex situ representation is feasible. Gardens and arboreta can more efficiently safeguard wild genetic diversity in this species by representing a greater proportion of rare alleles, possibly by sampling more maternal lines. For *Q. acerifolia*, results demonstrate that botanic gardens need to safeguard more wild individuals than what has previously been indicated using microsatellites, to safeguard 95% of the total wild allelic diversity of this species. If strong representation of rare alleles is desired (e.g., greater than ~50%), then ex situ sample sizes will need to be even higher.

Our findings of insufficient ex situ representation in *Q. acerifolia* reflect a scenario that seems likely for many ex situ collections assessed using microsatellite markers. That ex situ representation of low frequency and rare alleles is lower using SNPs versus microsatellites results from the higher numbers of loci generated by SNP methods generally. Detecting uncommon alleles requires an increased sampling of the genome of individuals, which might be unlikely for a marker typically yielding 10–20 loci. SNPs allow for orders of magnitude more loci than microsatellites, and in genomic regions that may be genic or nongenic, which in turn leads to an increased number of less common alleles. These less common alleles are less likely to be represented ex situ at the minimum sample sizes suggested by microsatellite data, and thus minimum sample size estimates increase in response to a more complete genomic sampling of individuals.

Given these methodological impacts on wild allelic representation, it's worth considering the value genetic diversity brings to ex situ collections. If a collection's intent is to safeguard imperiled species against all possible threats, then the importance of protecting rare genetic polymorphism will be greater, and minimum sample size estimates generated using SNP data should be followed. This is because several traits key to species survival, such as disease resistance, often occur at low frequencies in wild populations (Sniezko & Koch, 2017). However, if the conservation goal of ex situ collections is instead the representation of more common alleles, then our results demonstrate that microsatellites remain a relevant marker for measurements of that representation, and provide an accurate, efficient, and cost‐effective assessment of the overall genetic diversity represented ex situ (Hauser et al., 2022; Hodel et al., 2016).

As this is the first genetic marker comparison in the context of assessing ex situ conservation success and minimum sample size recommendations, it is important to specify limitations of our approach and how they might be influencing our findings. Most noteworthy is that, as in any other bioinformatic analysis (Shafer et al., 2017), our measures of ex situ allelic representation using SNPs are impacted by our approaches for filtering SNP loci. While we demonstrated the impacts of some approaches by analyzing SNP datasets in a factorized way (de novo assembly versus reference alignment; R0 versus R80 missing data filters), the complexity of genomic approaches limits the ability of any single study to comprehensively consider every filtering decision which could impact ex situ representation results. A more stringent SNP filtering approach than what was utilized in this comparison may not greatly change the relative patterns of “Total” ex situ representation levels across marker types observed here, simply because even after filtering there would still remain orders of magnitude more SNP loci as compared to microsatellites. However, a more restrictive SNP filtering approach could have a more pronounced effect on the relative representation levels of low frequency and rare alleles across markers—determining this would require a study with a scope larger than what we achieve here. Given the relative complexity of processing SNP datasets, future assessments of ex situ genetic diversity using SNPs would benefit from methods for estimating how many loci are required in order to confidently predict the number of samples needed to represent a specified threshold of the total allelic diversity in a wild population. Finally, while our analyses here quantified ex situ representation at the level of individual SNP loci, an alternative approach might consider diversity at the microhaplotype level (Reeves & Richards, 2017), which would likely impact ex situ conservation metrics.

Our results augment previous ex situ conservation research that has utilized microsatellites, and the recommendations to botanic gardens and arboreta based on that research. If a high degree of confidence in the measure of ex situ representation of rare alleles is important, then SNP loci, ideally derived from a reference alignment with a closely related organism, should be considered for determining relevant thresholds. Meanwhile, if the costs or complexities of processing SNP datasets are prohibitive (Puckett, 2017), then microsatellites remain an informative alternative for determining the overall genetic representation of an ex situ collection. Future research should assess whether the results described here apply to taxa with different demographic or life history traits from the two rare oaks we surveyed. Additionally, ex situ conservation assessments utilizing SNPs should establish a means of determining how many SNP loci are required for generating reliable minimum sample size estimates. These efforts will contribute to ongoing work ensuring that botanic gardens and arboreta act as valuable resources for safeguarding the genetic diversity of threatened plant species (Westwood et al., 2021).

## AUTHOR CONTRIBUTIONS

Conceptualization—SMH and ACK; Data curation—all authors; Formal analysis—all authors, led by ACK; Methodology—all authors, led by ACK and SMH; Project administration—SMH and ACK; Visualization—ACK, EKS, SMH; Writing—ACK; Editing—all authors, led by ACK.

## CONFLICT OF INTEREST STATEMENT

The authors declare no conflict of interest.

## Supporting information


Figure S1.

Figure S2.

Figure S3.

Figure S4.

Figure S5.

Figure S6.

Figure S7.

Figure S8.

Figure S9.

Figure S10.

Figure S11.

Figure S12.

Figure S13.

Figure S14.

Figure S15.

Figure S16.

Figure S17.

Figure S18.

Figure S19.

Figure S20.

Figure S21.

Figure S22.

Figure S23.

Figure S24.

Figure S25.

Figure S26.

Figure S27.

Figure S28.

Figure S29.

Figure S30.

Data S1.

## Data Availability

Genetic data: Raw, demultiplexed sequence files in .fasta format can be obtained on the National Institute of Health's Sequence Read Archive (SRA; BioProject PRJNA770293). Scripts used for quality filtering, SNP Calling, de novo assembly, reference alignment, ex situ representation analyses, and population clustering are available on GitHub. Sample metadata: Metadata are also stored in the SRA (BioProject PRJNA770293).
