# Supplementary figures and images for: Ex situ conservation of two rare oak species using microsatellite and SNP markers
Source: Evol Appl. 2024 Mar 22;17(3):e13650. doi: 10.1111/eva.13650 (PMC10960078; doi:10.1111/eva.13650)

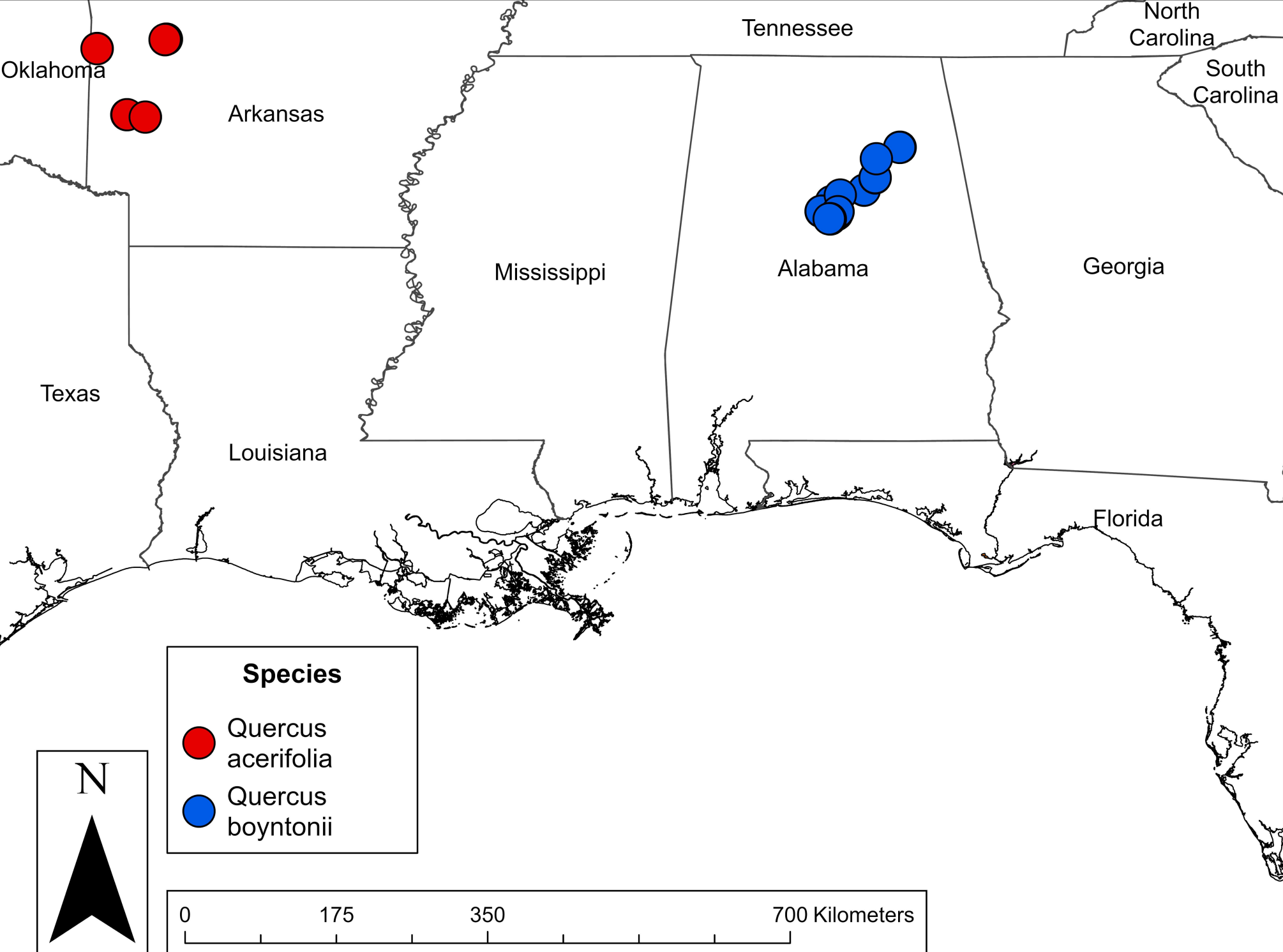

Supplement: Supplementary file 1 — Figure S1. [file EVA-17-e13650-s020.pdf]

## Q. acerifolia: assembly metrics across m values

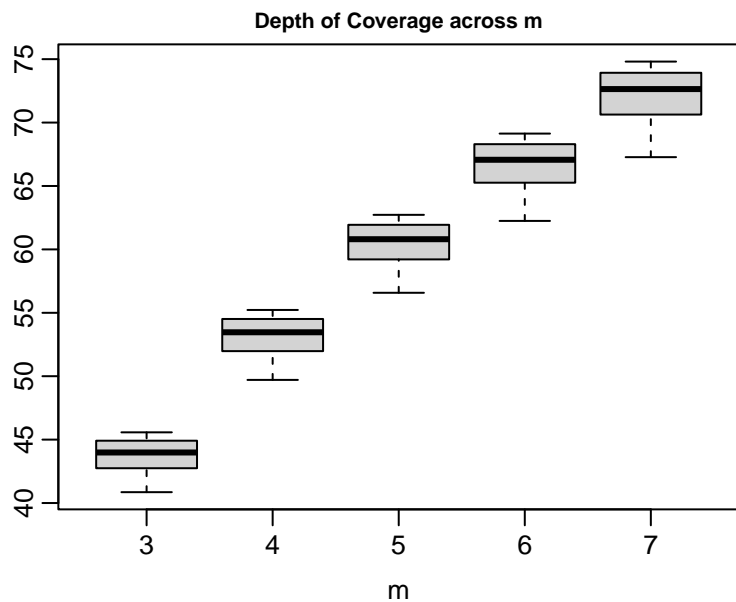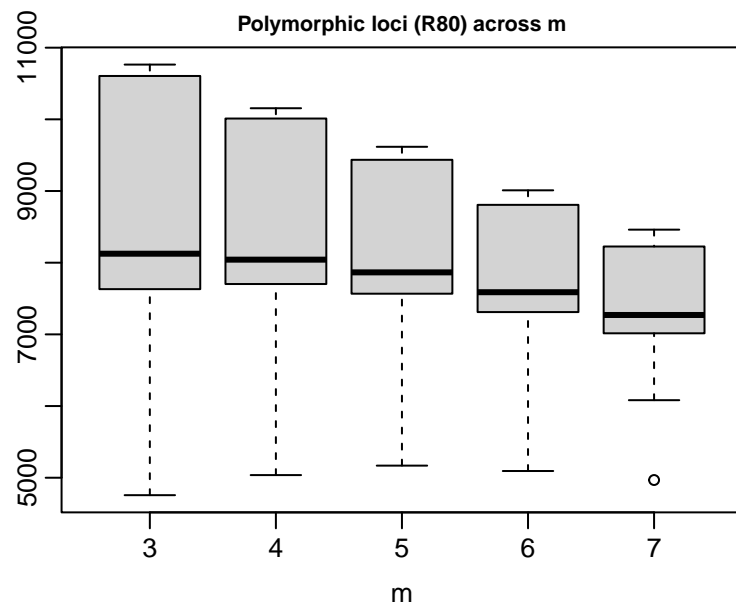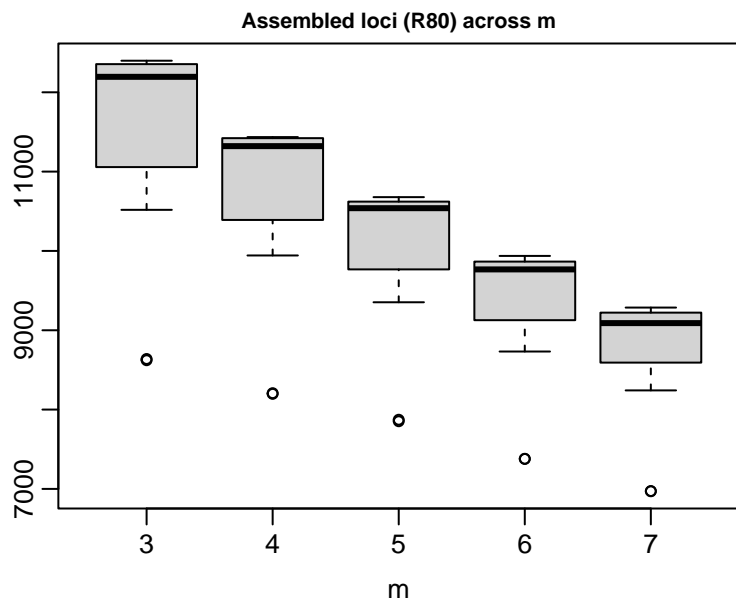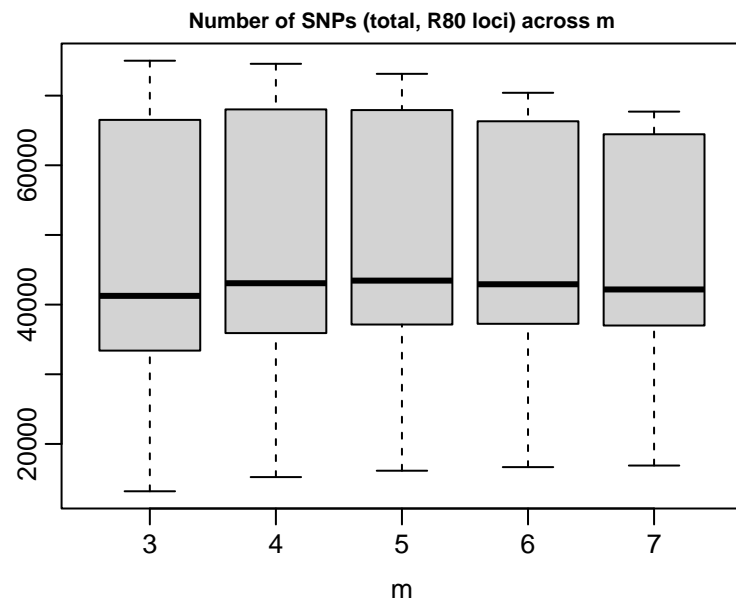

Supplement: Supplementary file 2 — Figure S2. [file EVA-17-e13650-s019.pdf]

# Q. acerifolia: assembly metrics across gt-alpha values

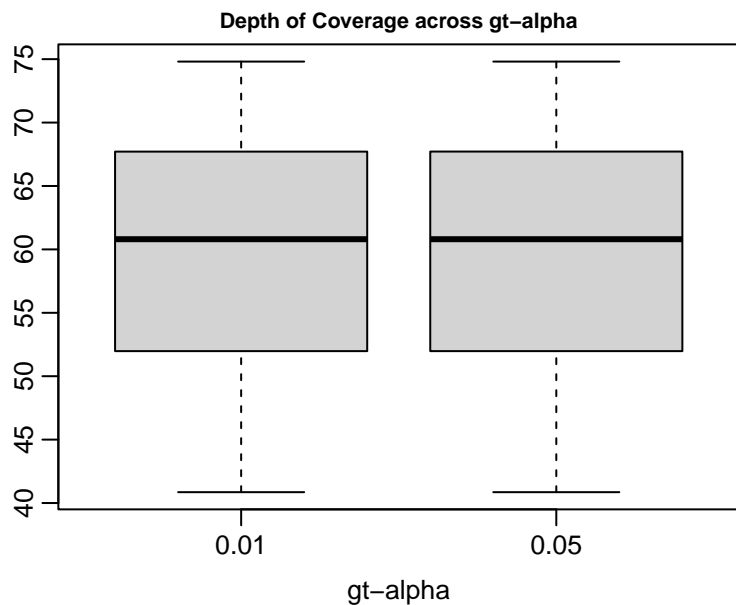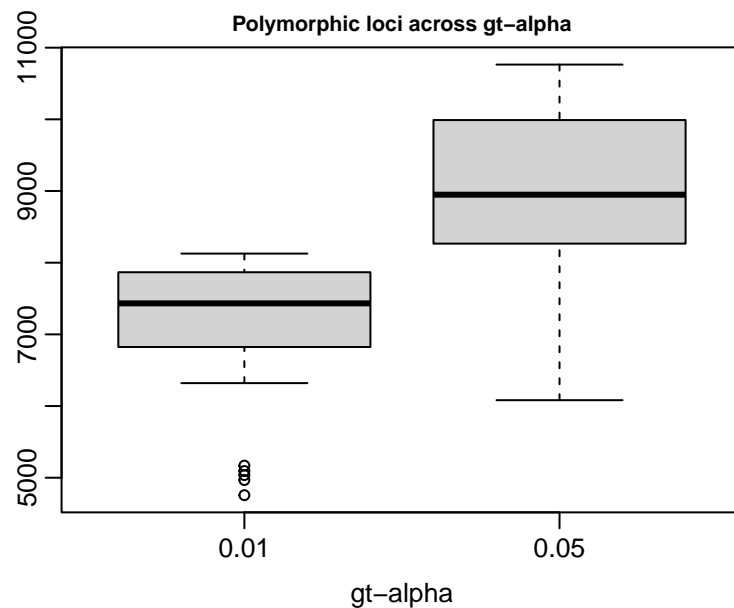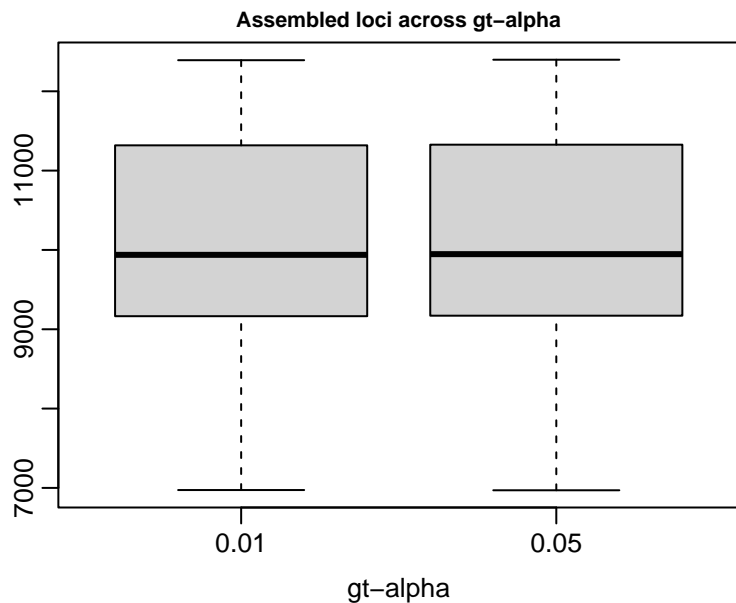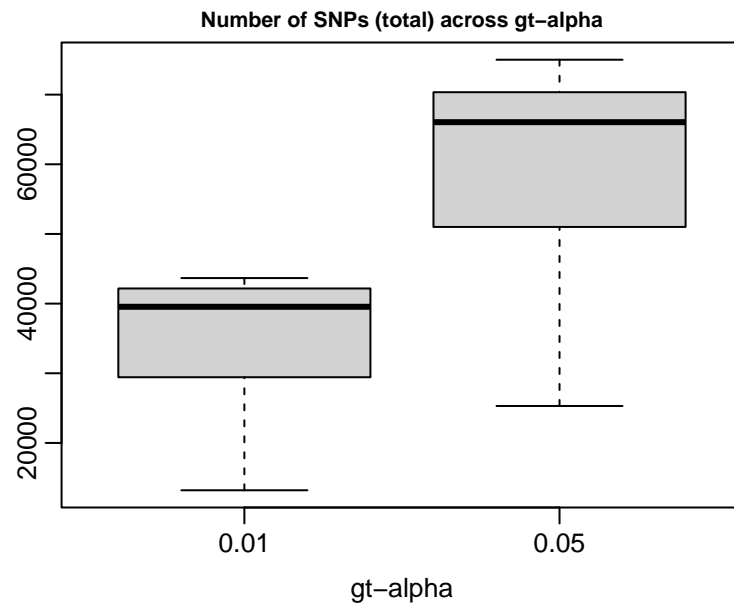

Supplement: Supplementary file 4 — Figure S4. [file EVA-17-e13650-s030.pdf]

# Q. boyntonii: assembly metrics across m values

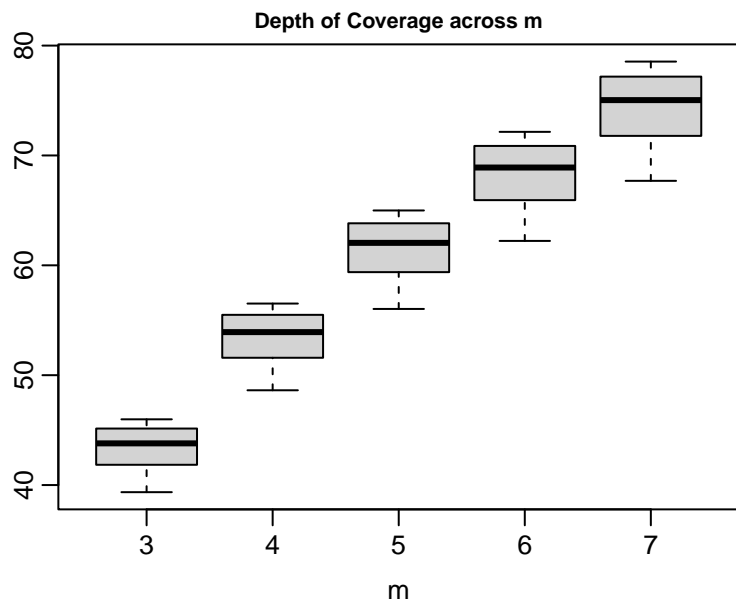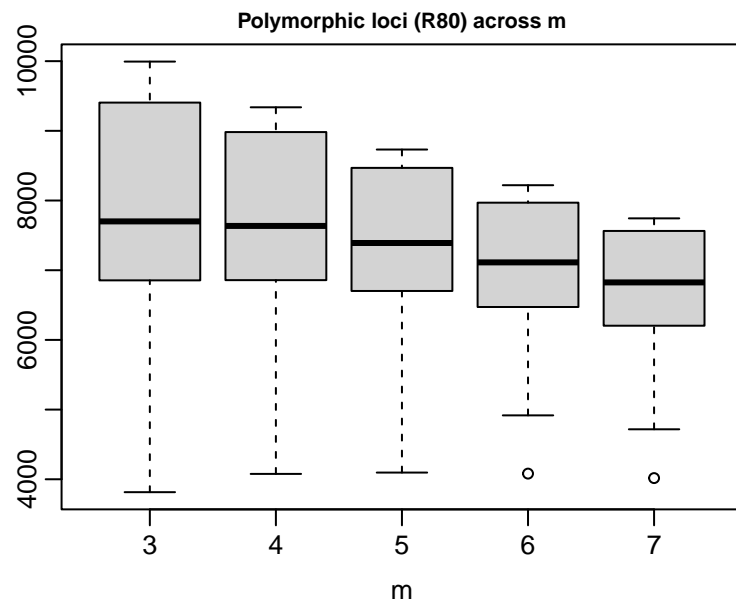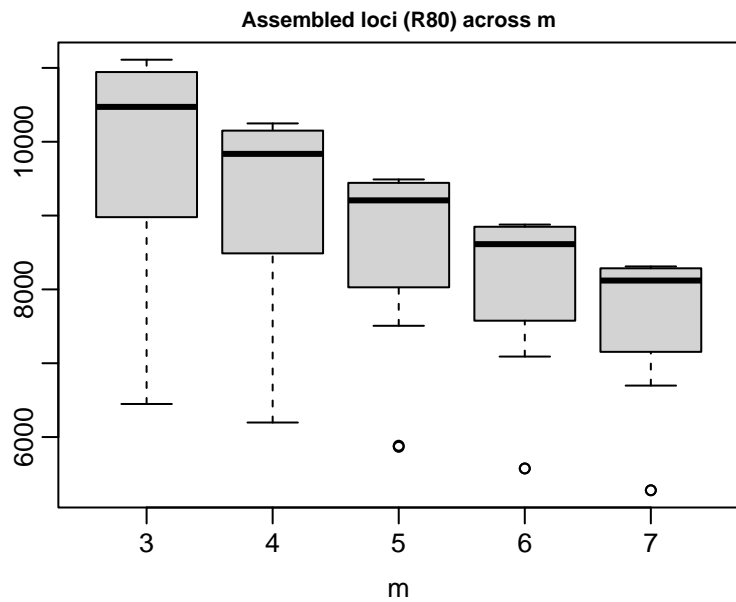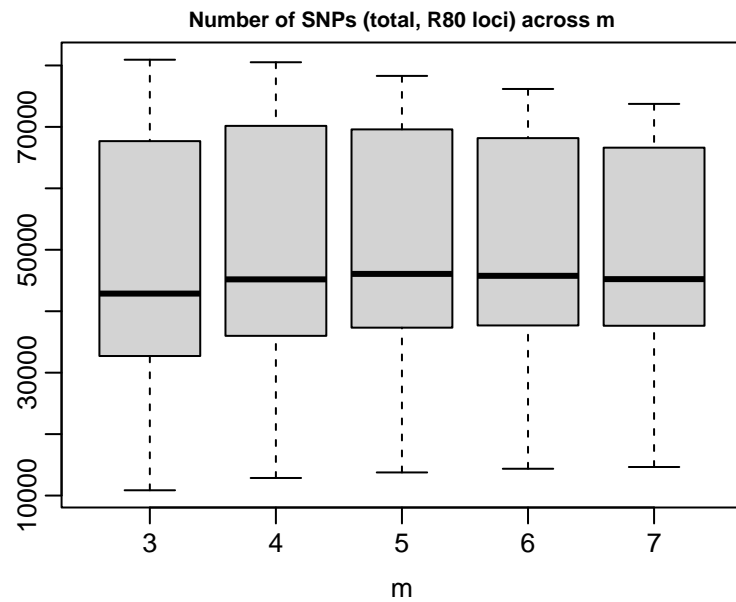

Supplement: Supplementary file 5 — Figure S5. [file EVA-17-e13650-s010.pdf]

# Q. boyntonii: assembly metrics across M/n values

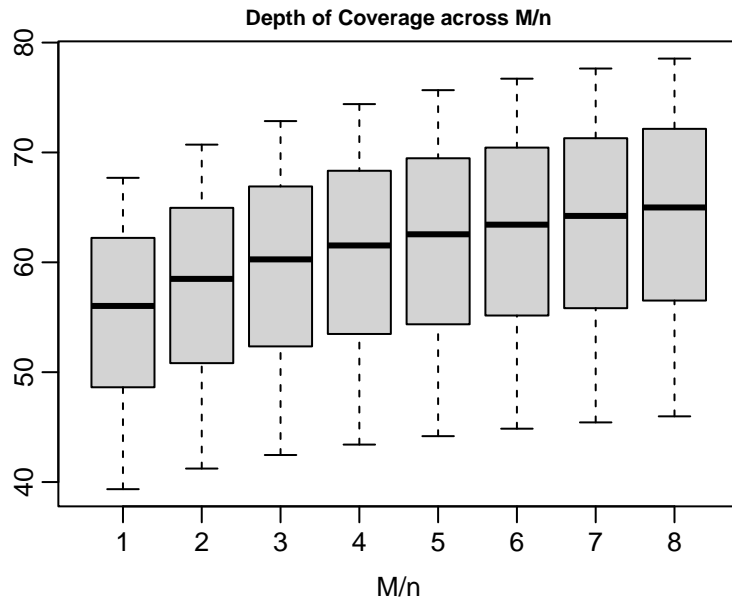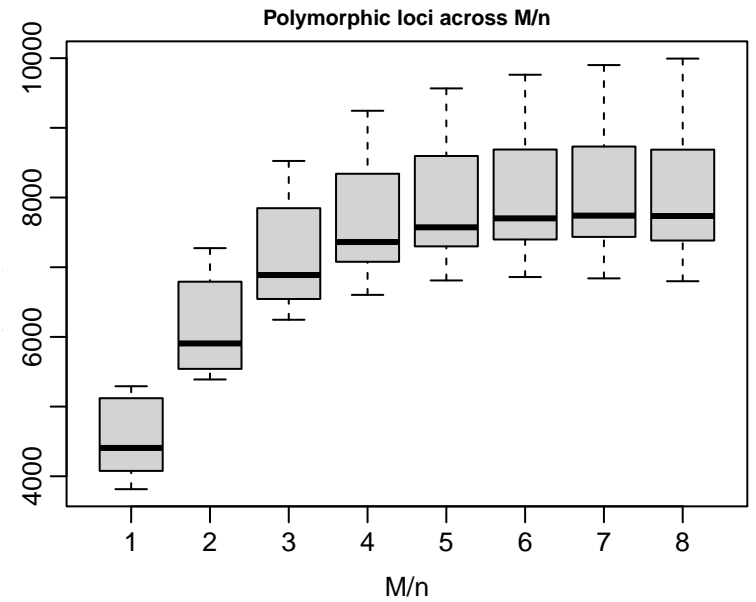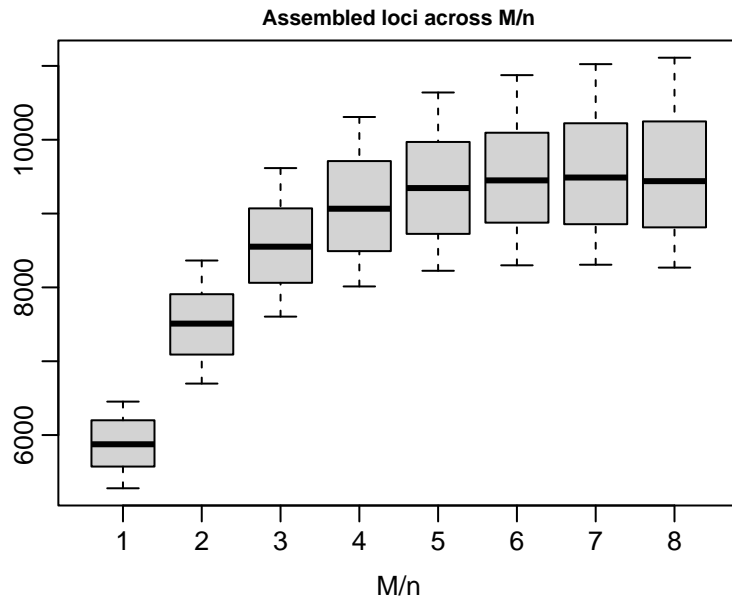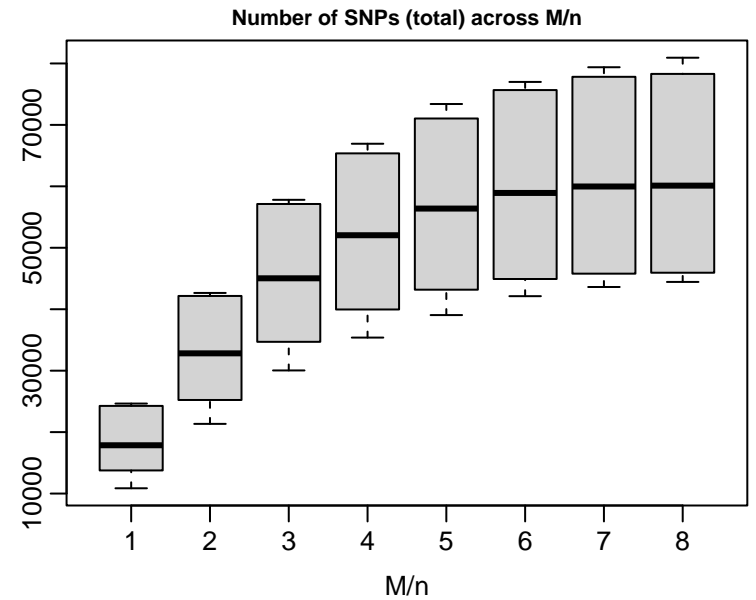

Supplement: Supplementary file 6 — Figure S6. [file EVA-17-e13650-s015.pdf]

# Q. boyntonii: assembly metrics across gt-alpha values

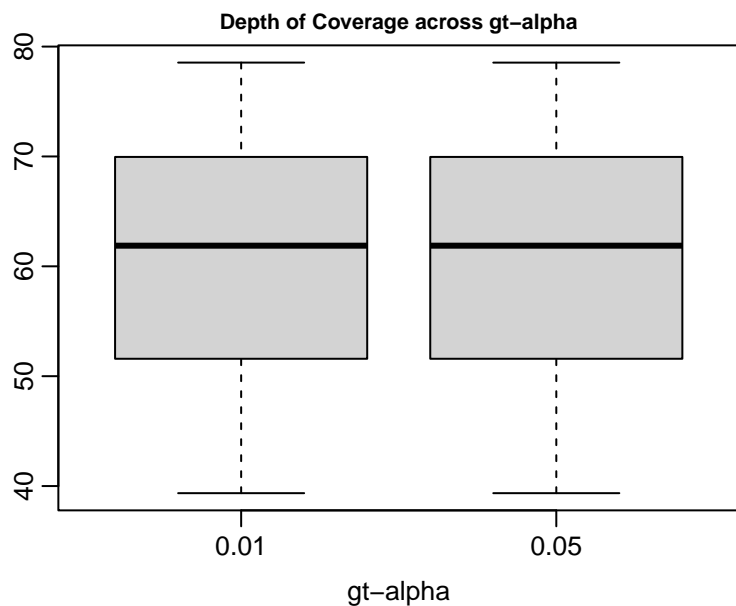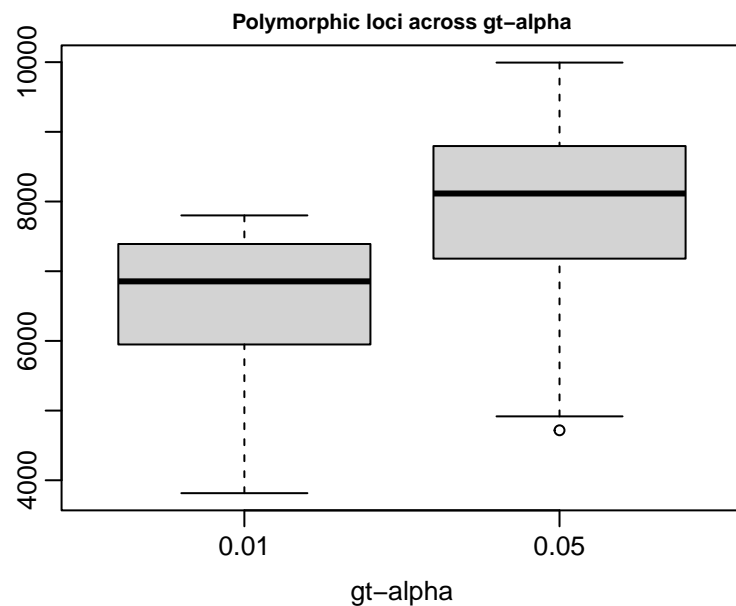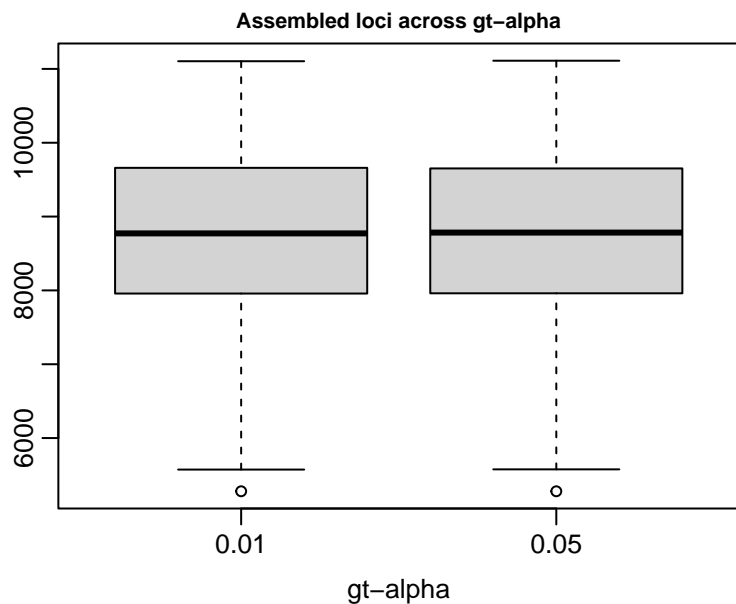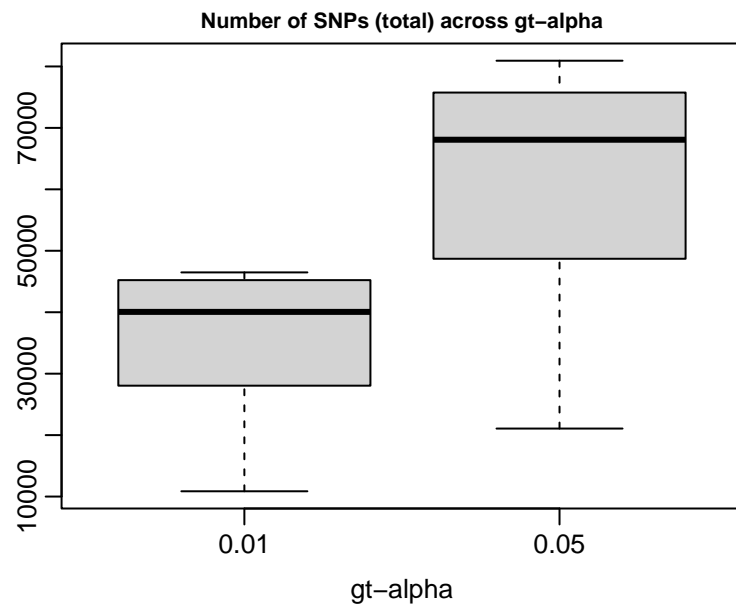

Supplement: Supplementary file 7 — Figure S7. [file EVA-17-e13650-s027.pdf]

# Quercus acerifolia: Wild allele frequency distributions

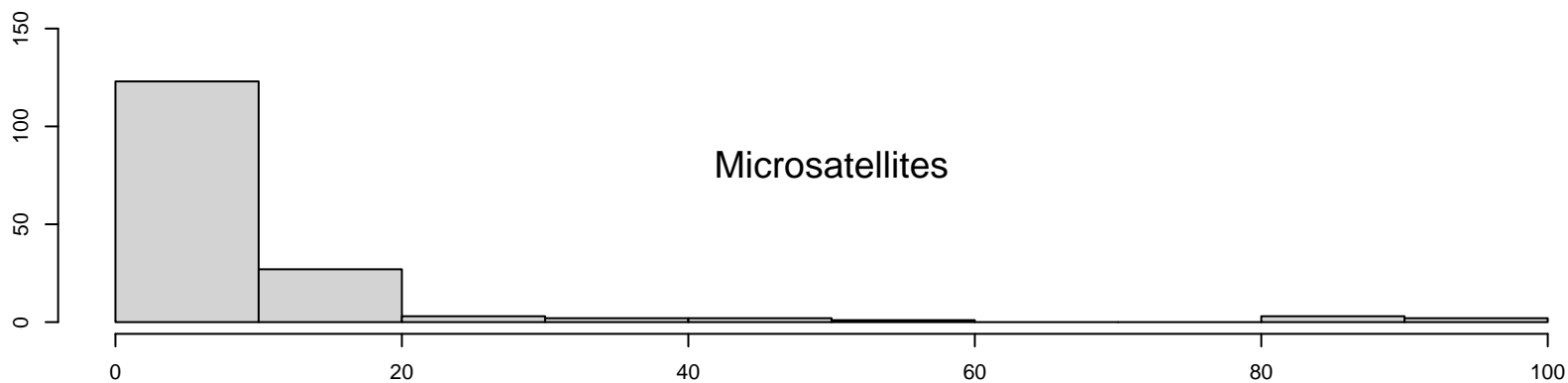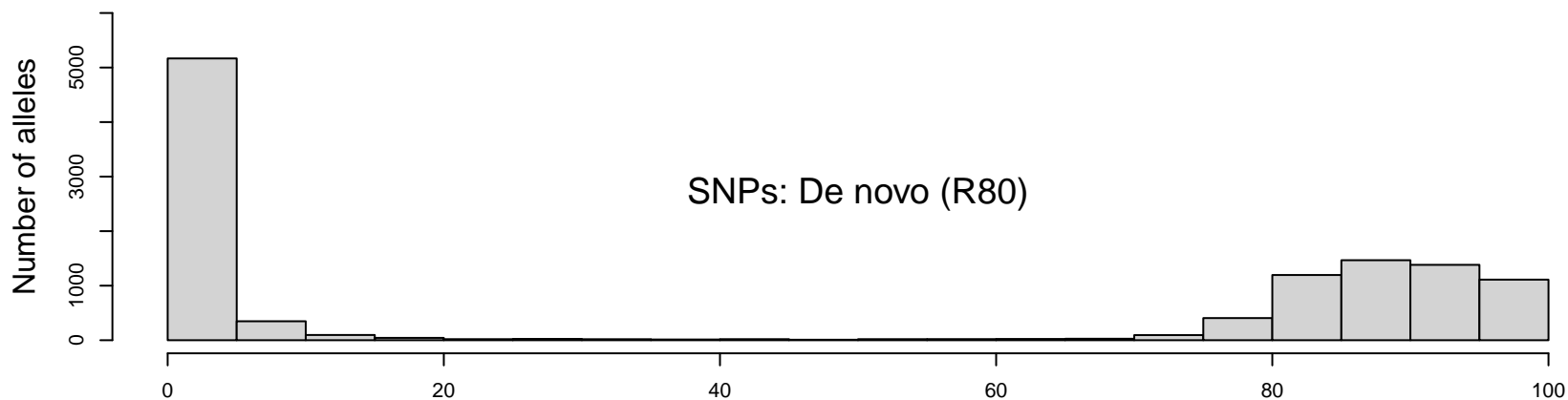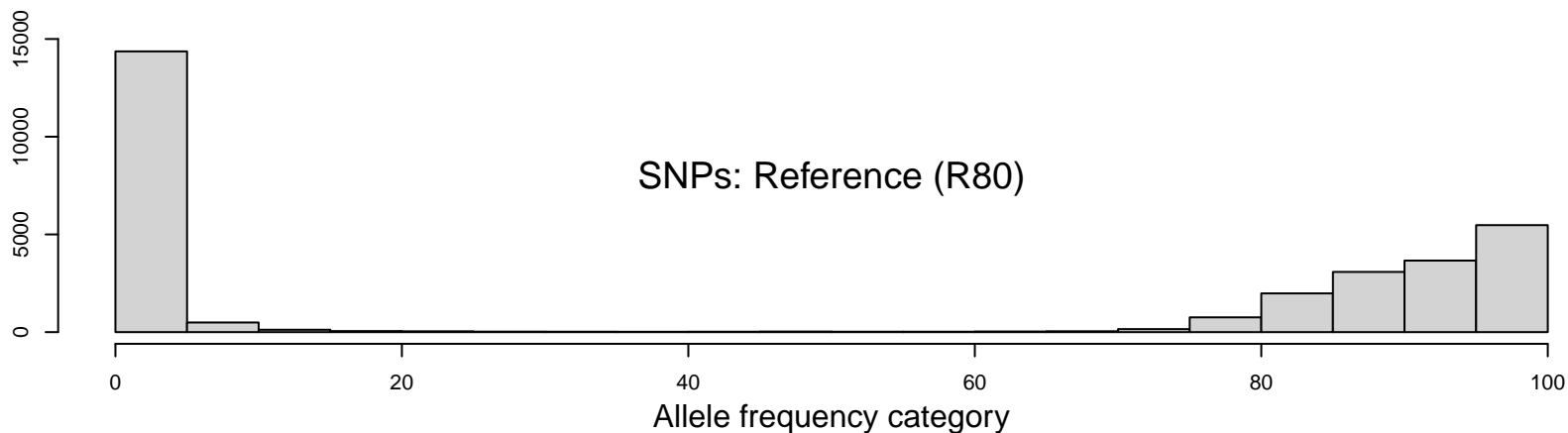

Supplement: Supplementary file 8 — Figure S8. [file EVA-17-e13650-s008.pdf]

# Quercus boyntonii: Wild allele frequency distributions

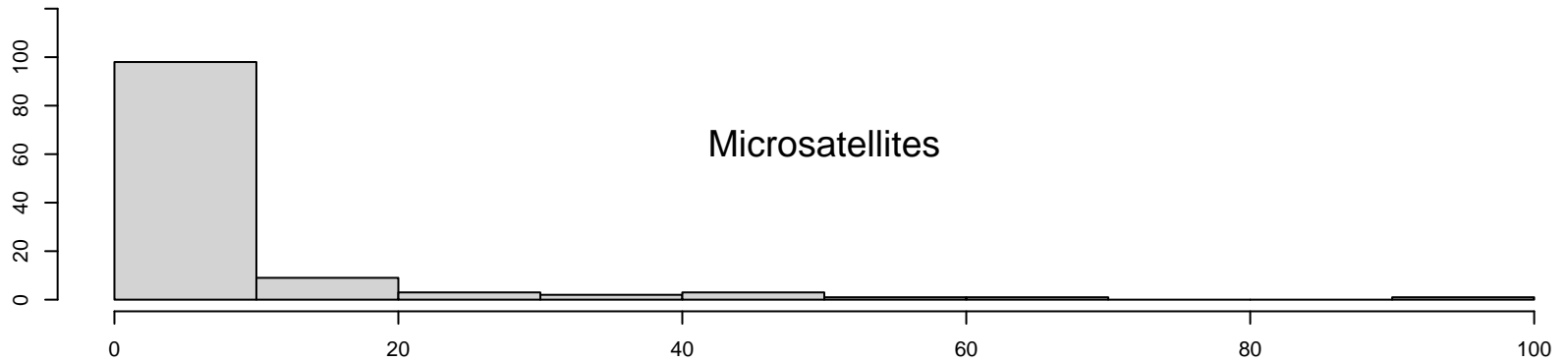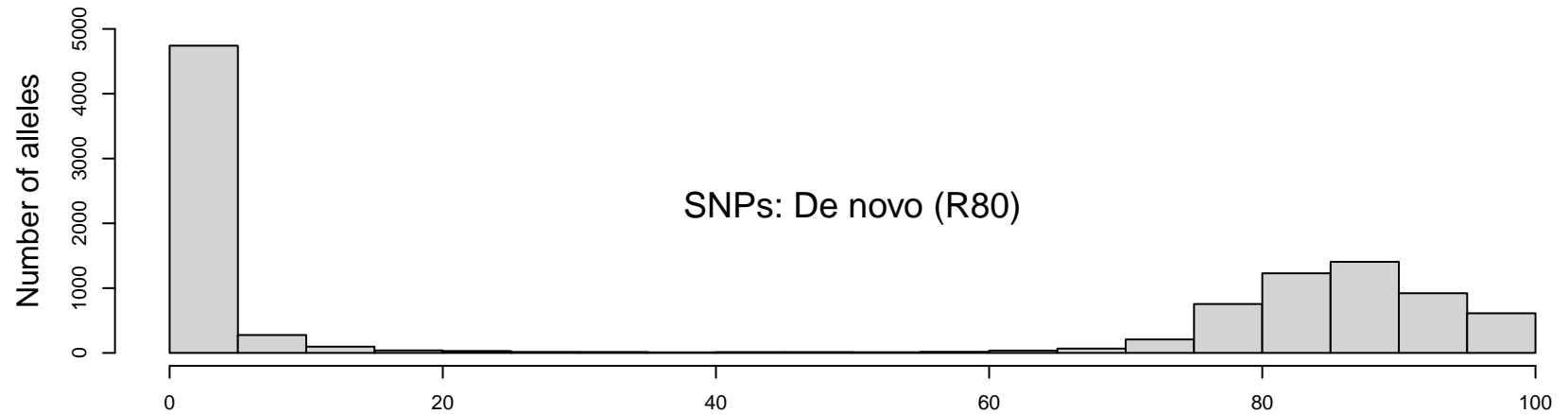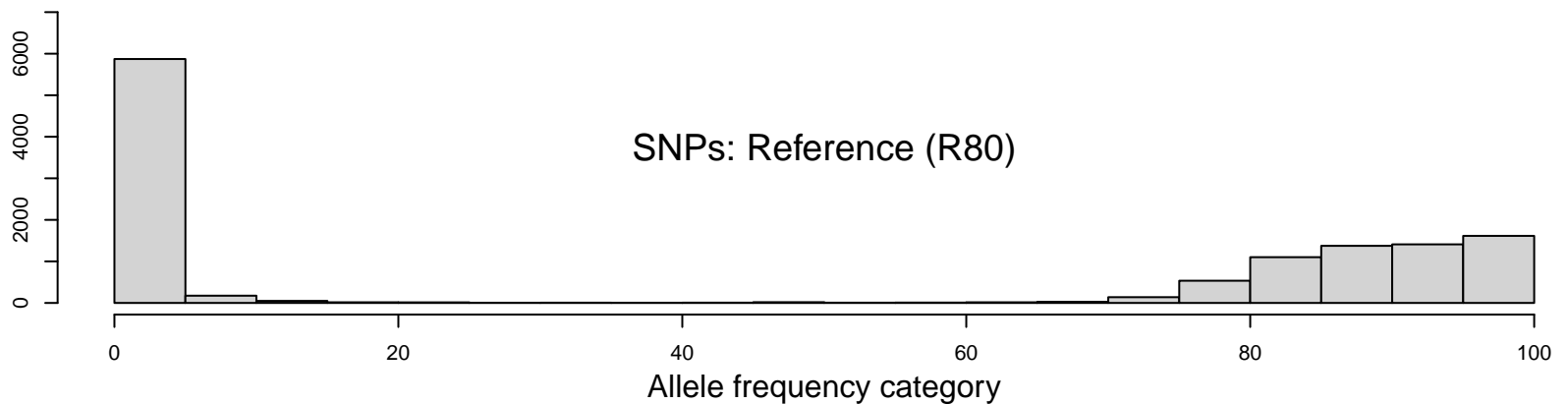

Supplement: Supplementary file 9 — Figure S9. [file EVA-17-e13650-s021.pdf]

# QUAC, Microsatellites (Complete: 164 samples)

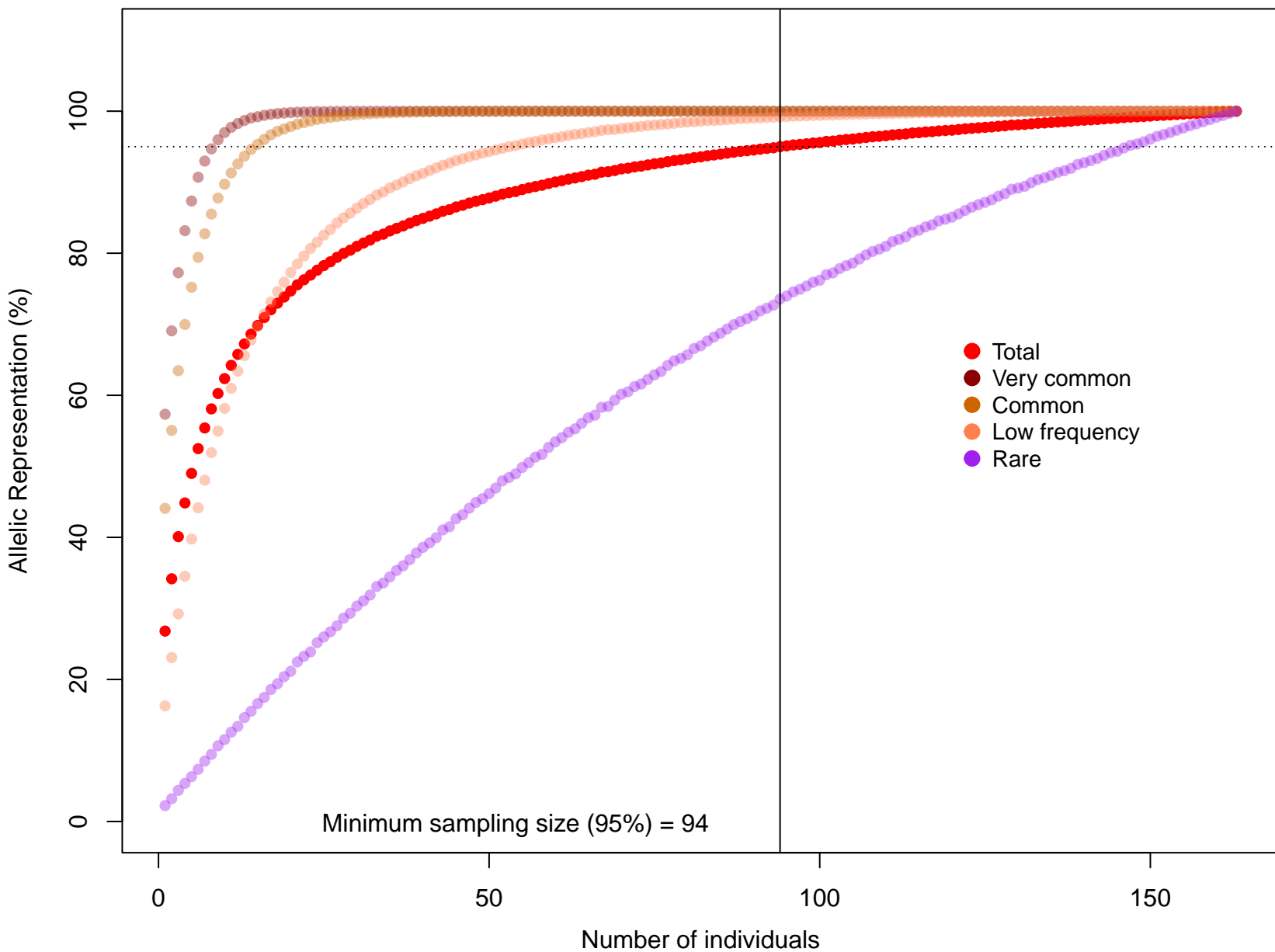

Supplement: Supplementary file 10 — Figure S10. [file EVA-17-e13650-s029.pdf]

# QUAC, Microsatellites (Subset: 91 samples)

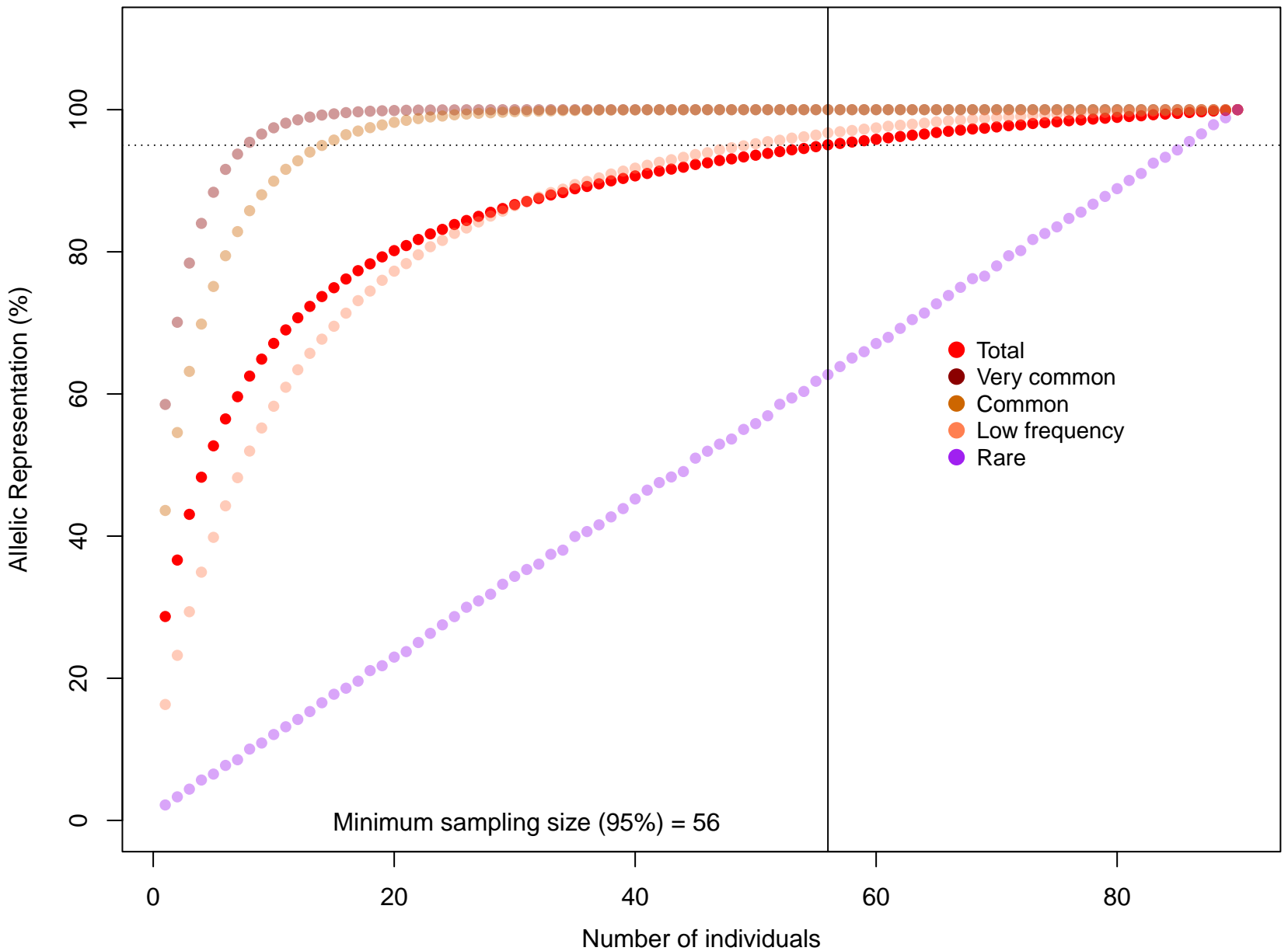

Supplement: Supplementary file 11 — Figure S11. [file EVA-17-e13650-s013.pdf]

**QUAC, SNPs: De novo, R0 (Complete: 91 samples)**

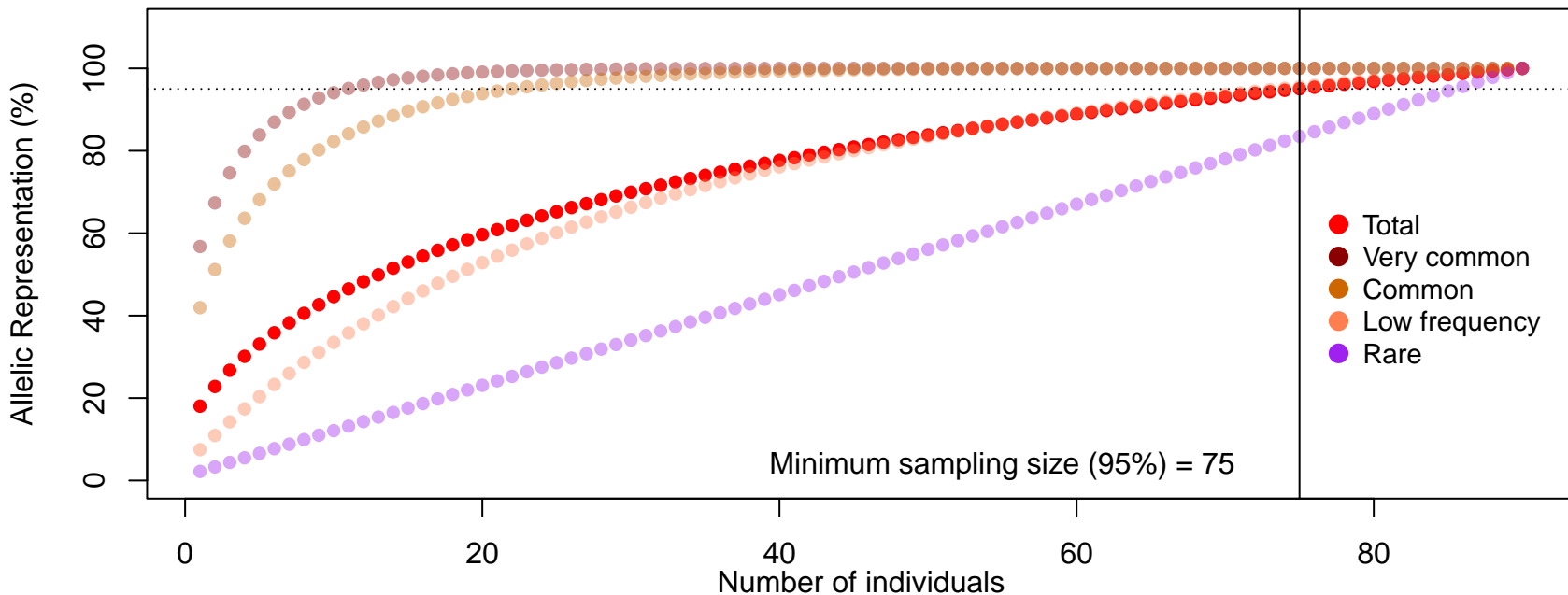

**QUAC, SNPs: De novo, R80 (Complete: 91 samples)**

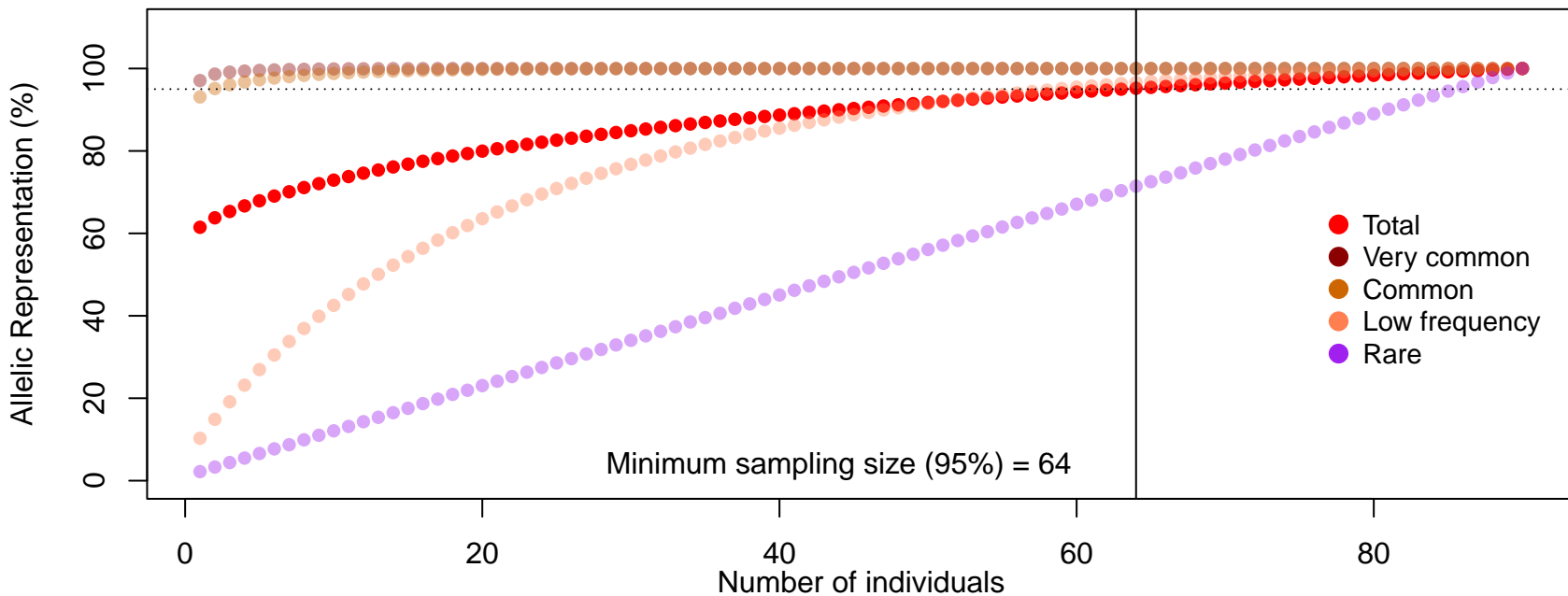

Supplement: Supplementary file 12 — Figure S12. [file EVA-17-e13650-s002.pdf]

**QUAC, SNPs: De novo, R0 (Subset: 91 samples)**

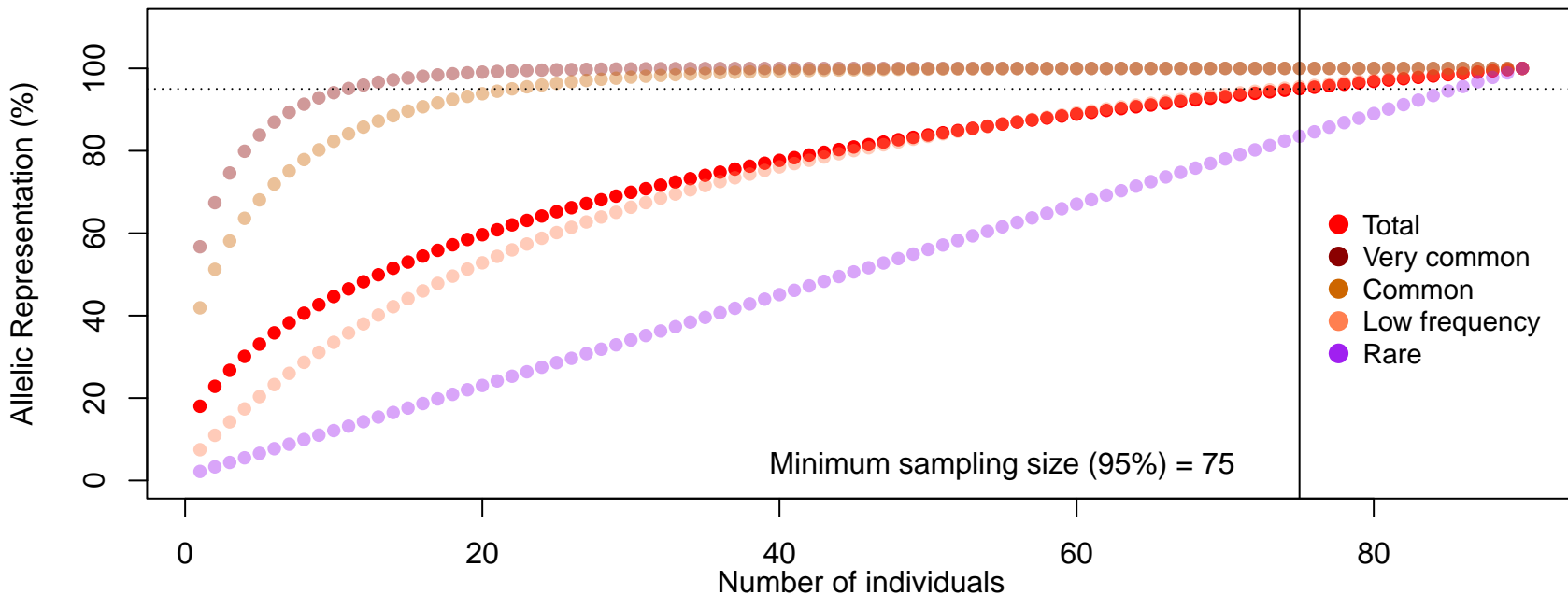

**QUAC, SNPs: De novo, R80 (Subset: 91 samples)**

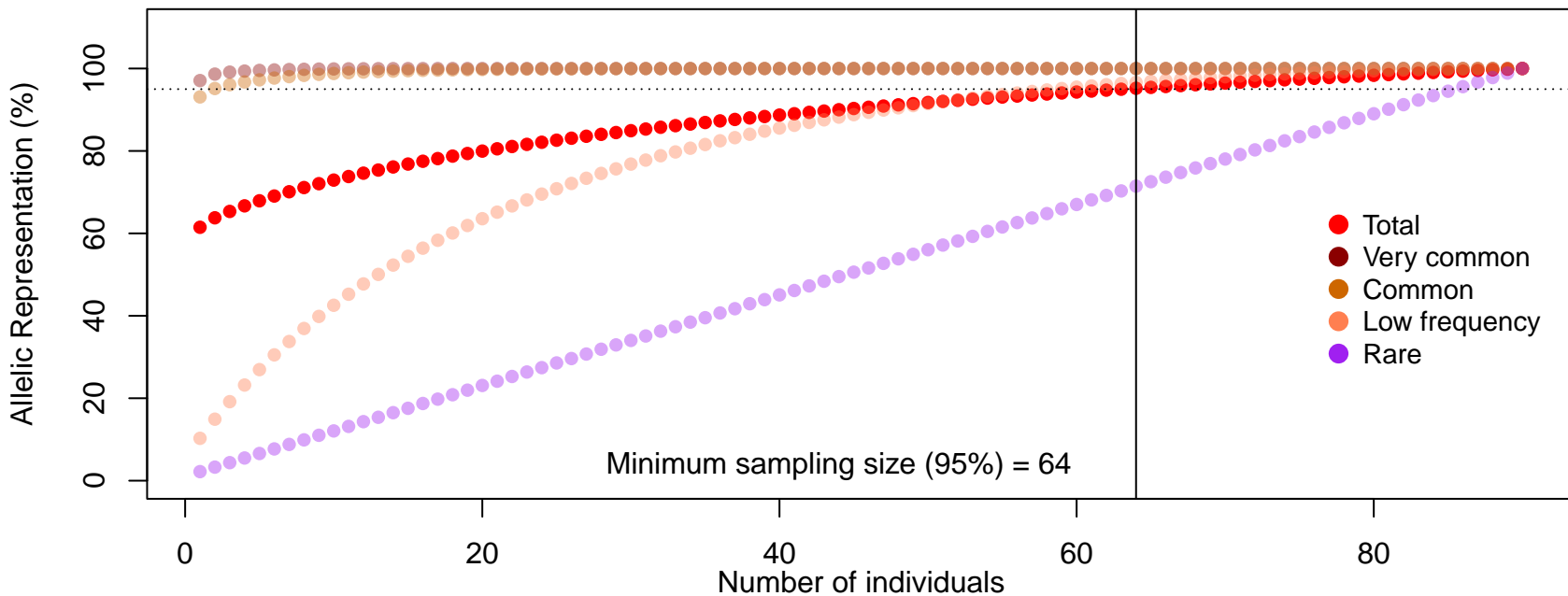

Supplement: Supplementary file 13 — Figure S13. [file EVA-17-e13650-s024.pdf]

**QUAC, SNPs: Reference, R0 (Complete: 91 samples)**

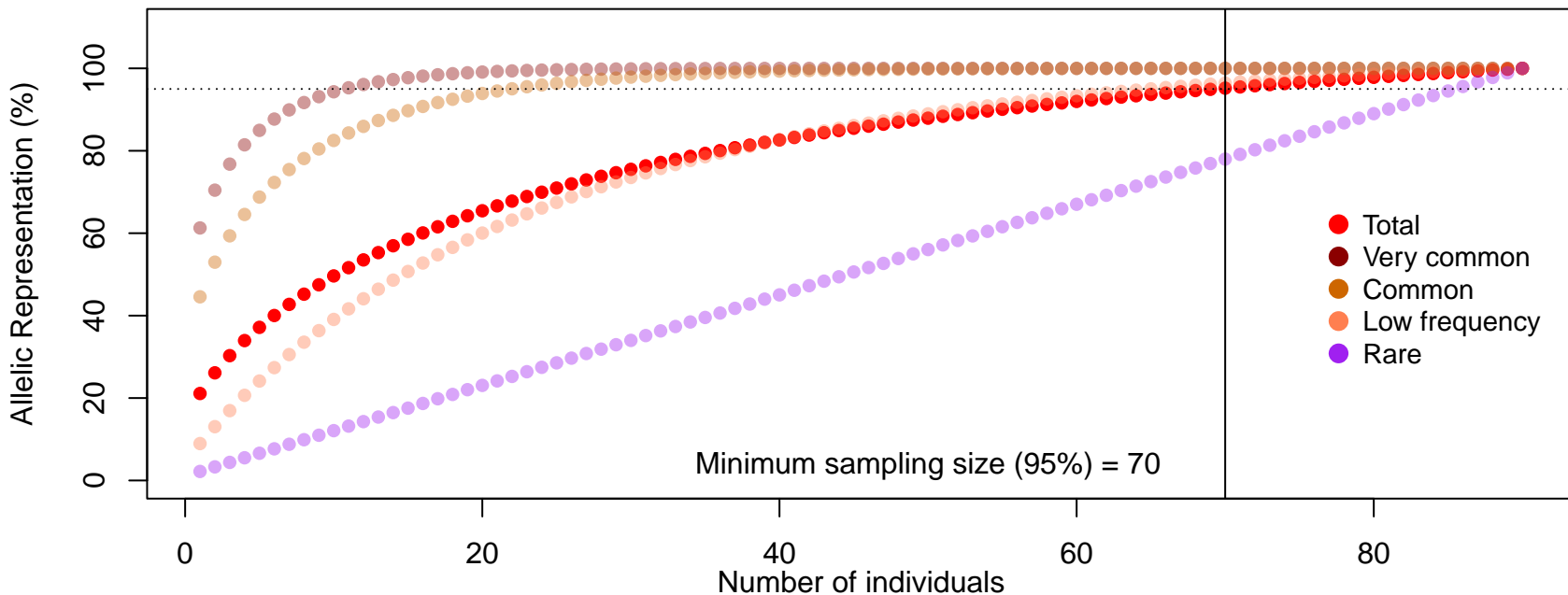

**QUAC, SNPs: Reference, R80 (Complete: 91 samples)**

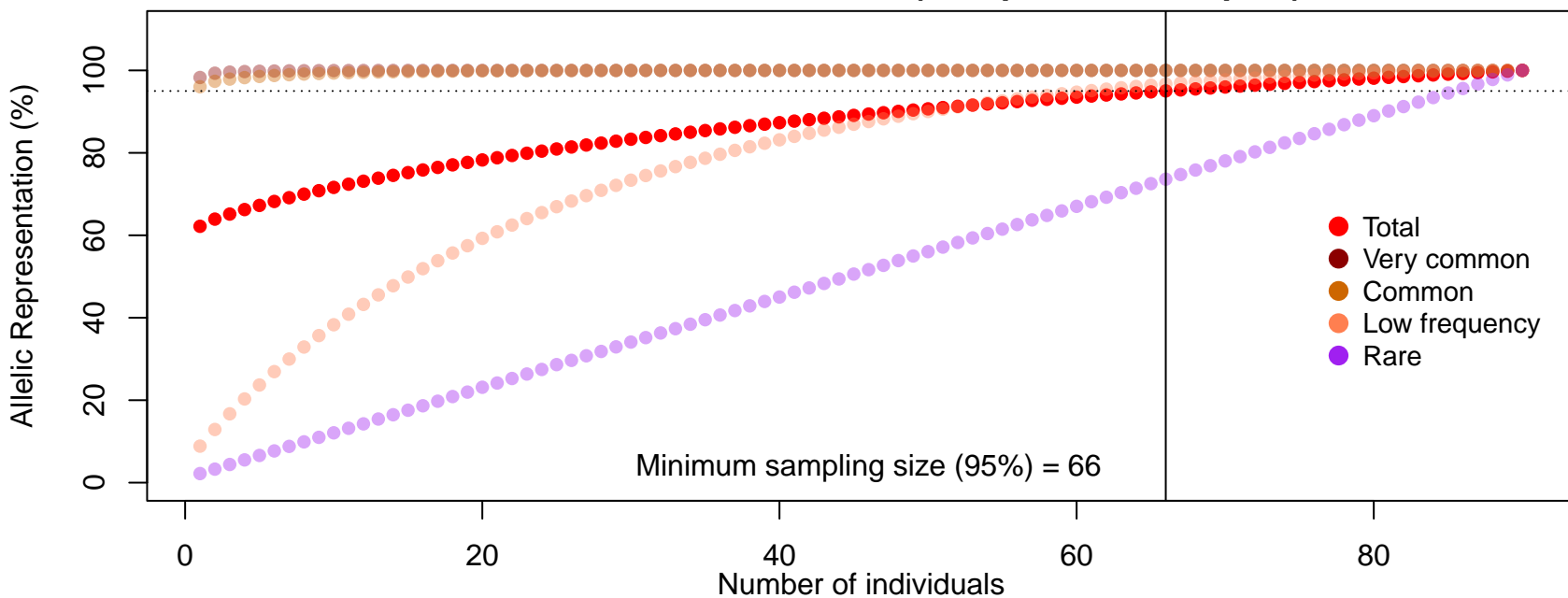

Supplement: Supplementary file 14 — Figure S14. [file EVA-17-e13650-s018.pdf]

**QUAC, SNPs: Reference, R0 (Subset: 91 samples)**

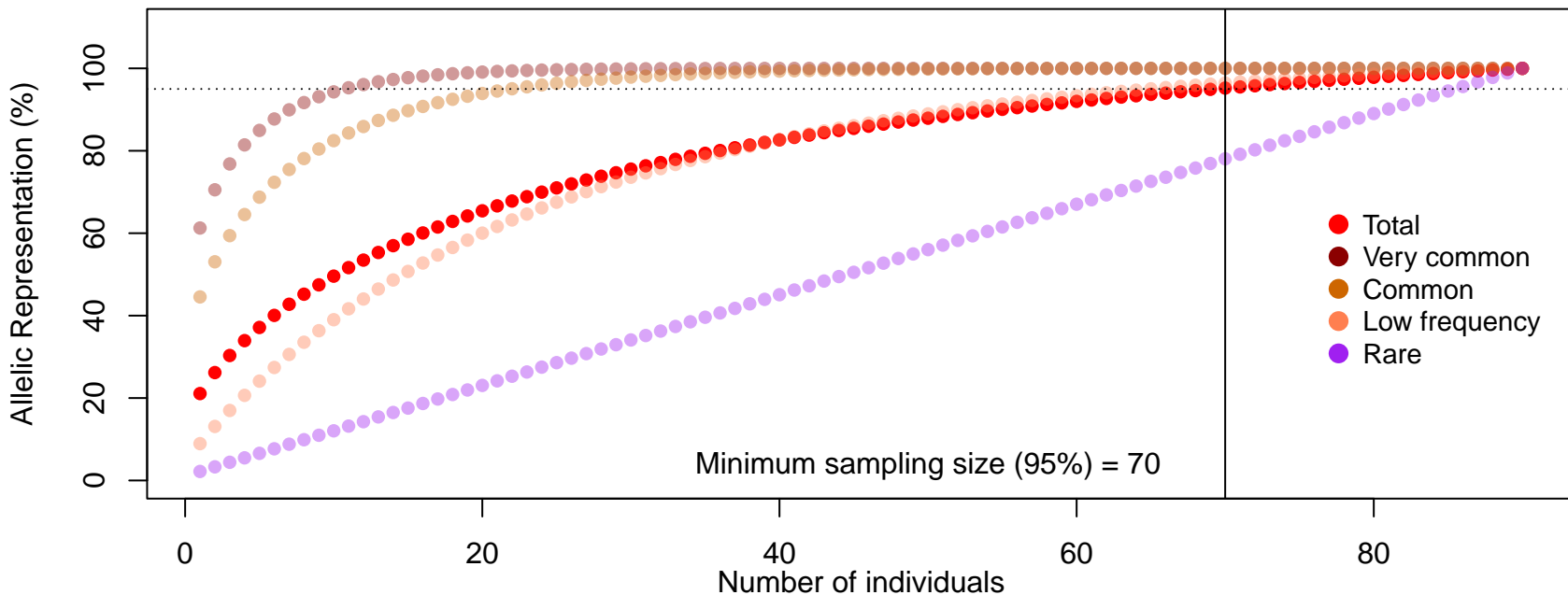

**QUAC, SNPs: Reference, R80 (Subset: 91 samples)**

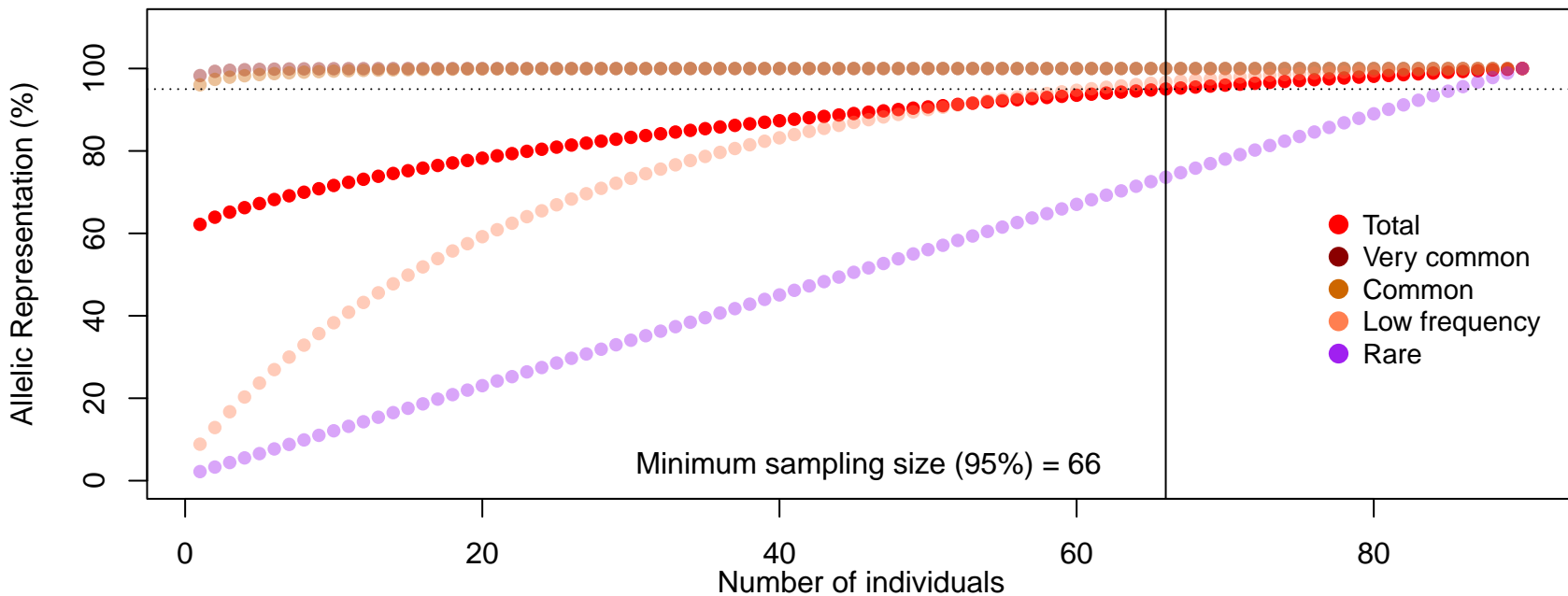

Supplement: Supplementary file 15 — Figure S15. [file EVA-17-e13650-s006.pdf]

# QUBO, Microsatellites (Complete: 245 samples)

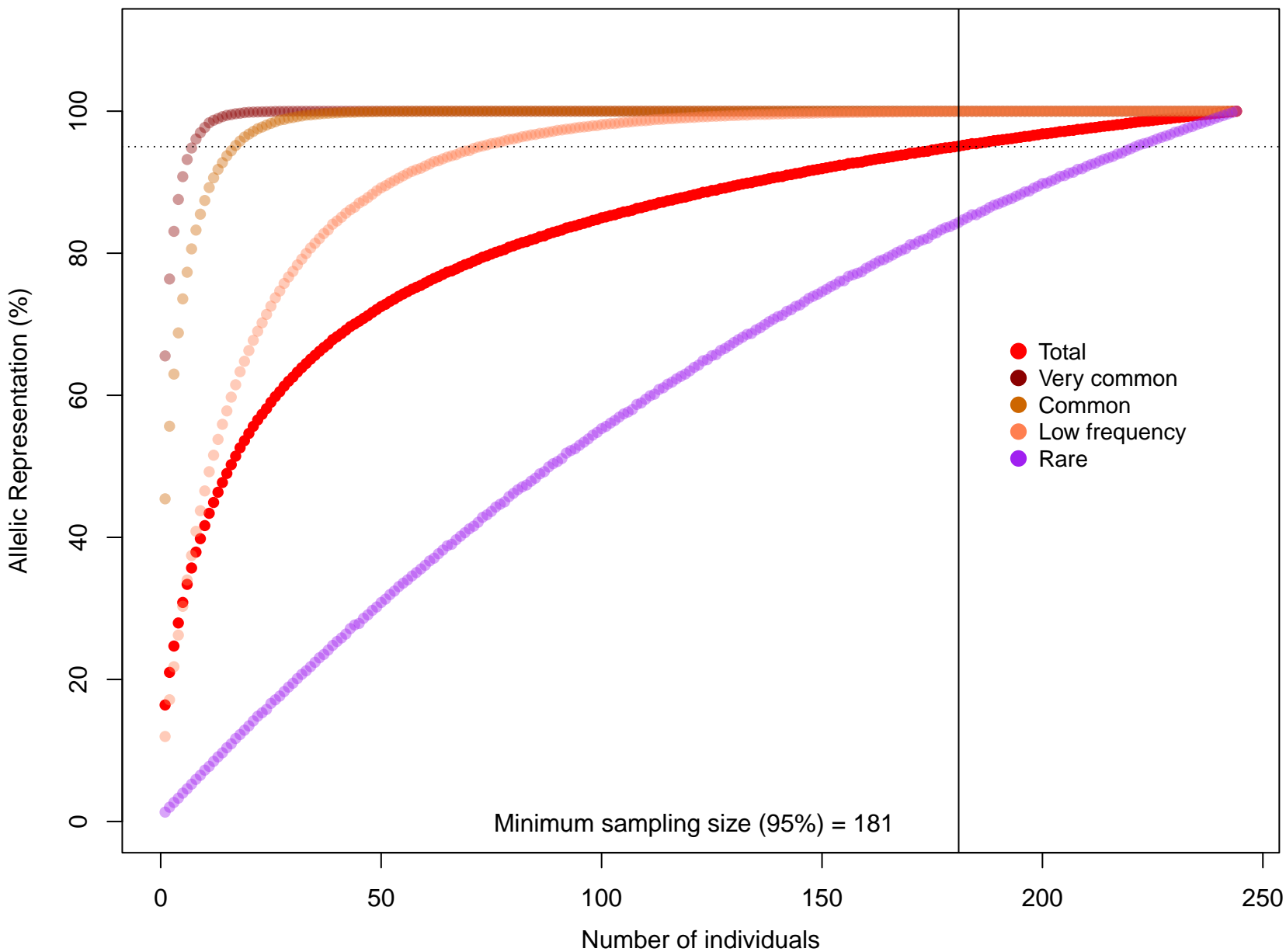

Supplement: Supplementary file 16 — Figure S16. [file EVA-17-e13650-s011.pdf]

# QUBO, Microsatellites (Subset: 94 samples)

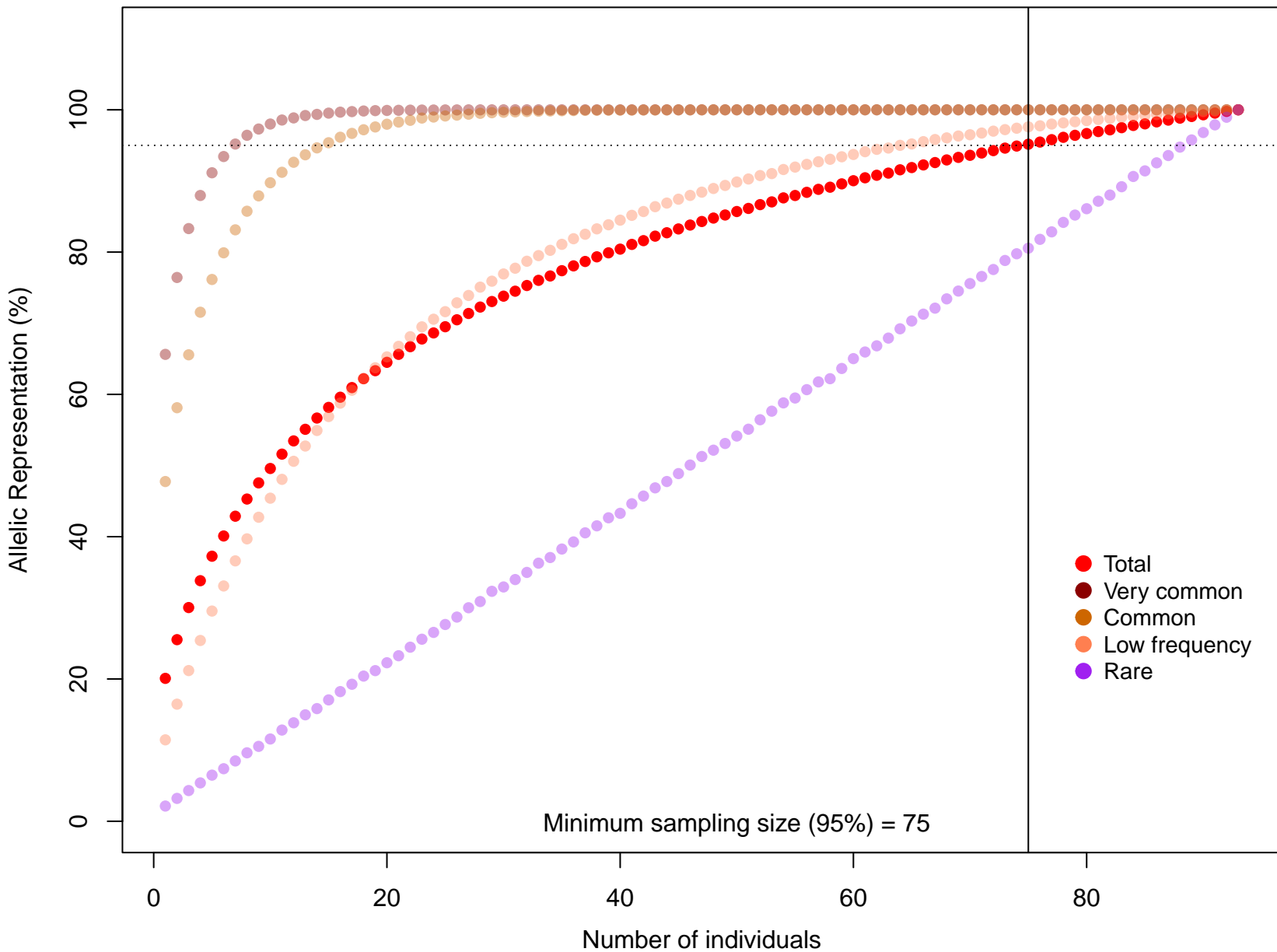

Supplement: Supplementary file 17 — Figure S17. [file EVA-17-e13650-s025.pdf]

**QUBO, SNPs: De novo, R0 (Complete: 95 samples)**

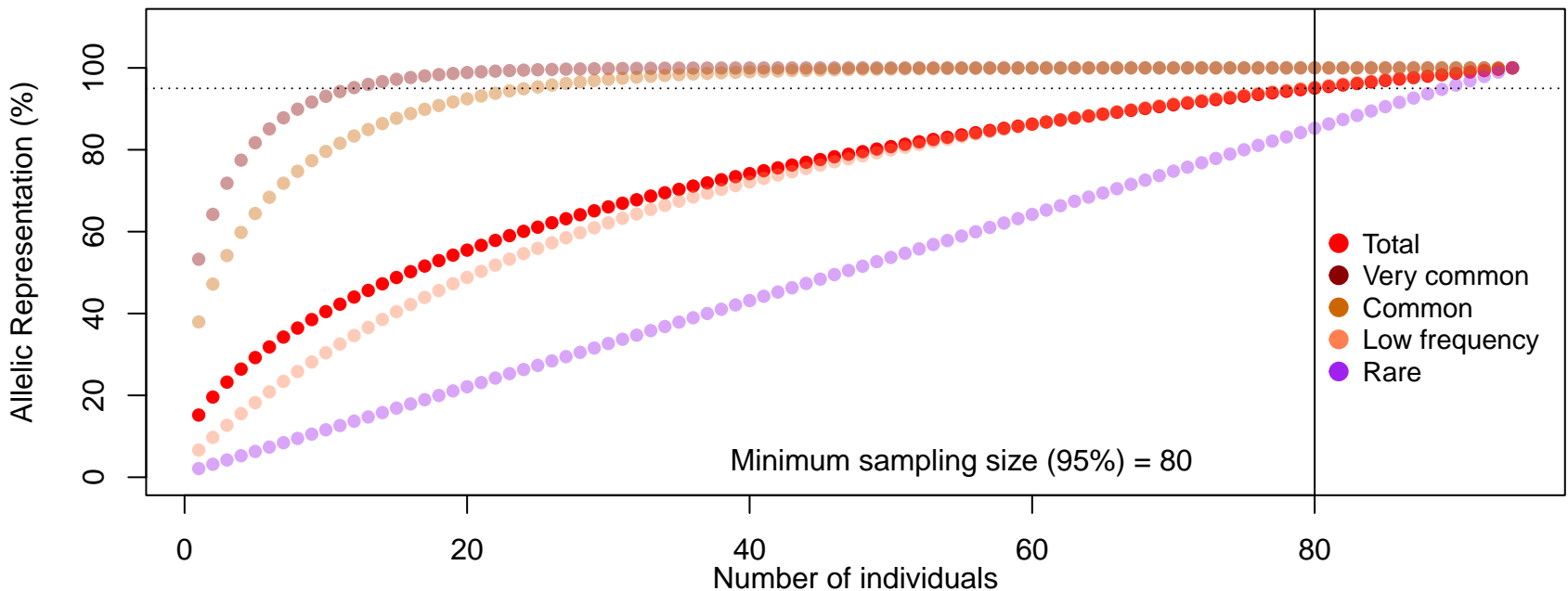

**QUBO, SNPs: De novo, R80 (Complete: 95 samples)**

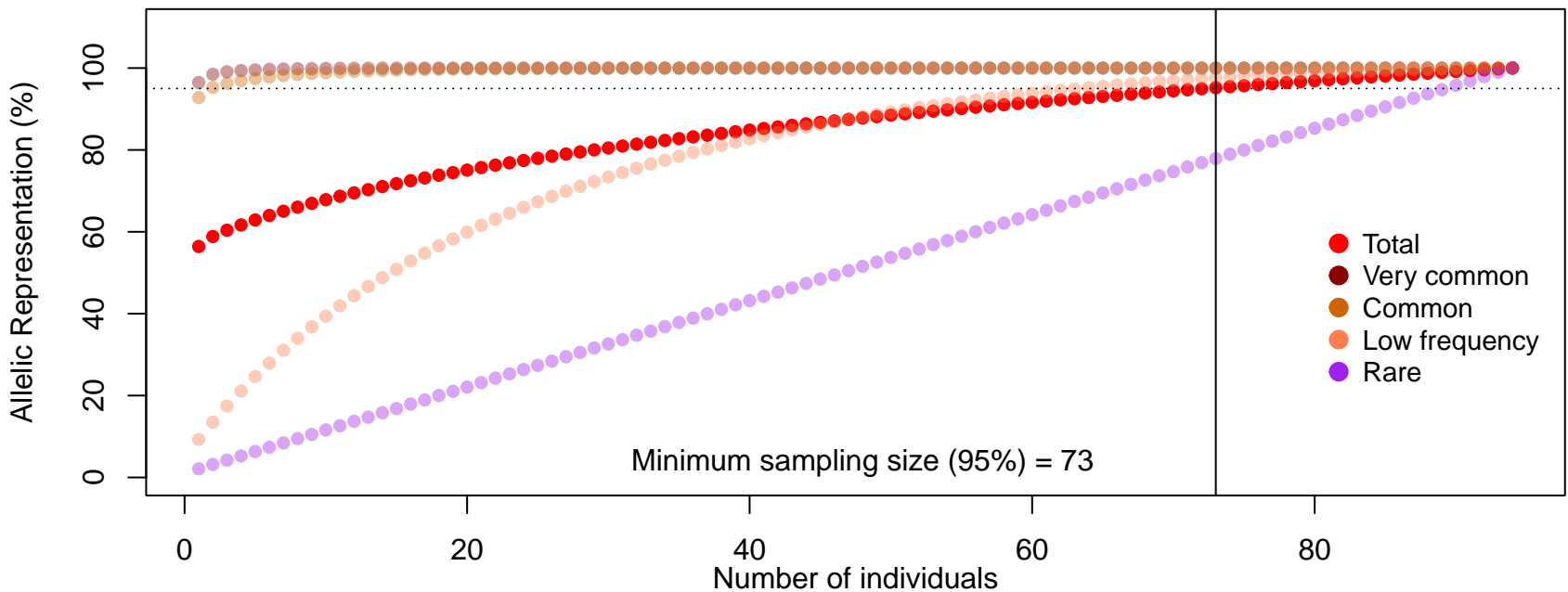

Supplement: Supplementary file 18 — Figure S18. [file EVA-17-e13650-s005.pdf]

**QUBO, SNPs: De novo, R0 (Subset: 94 samples)**

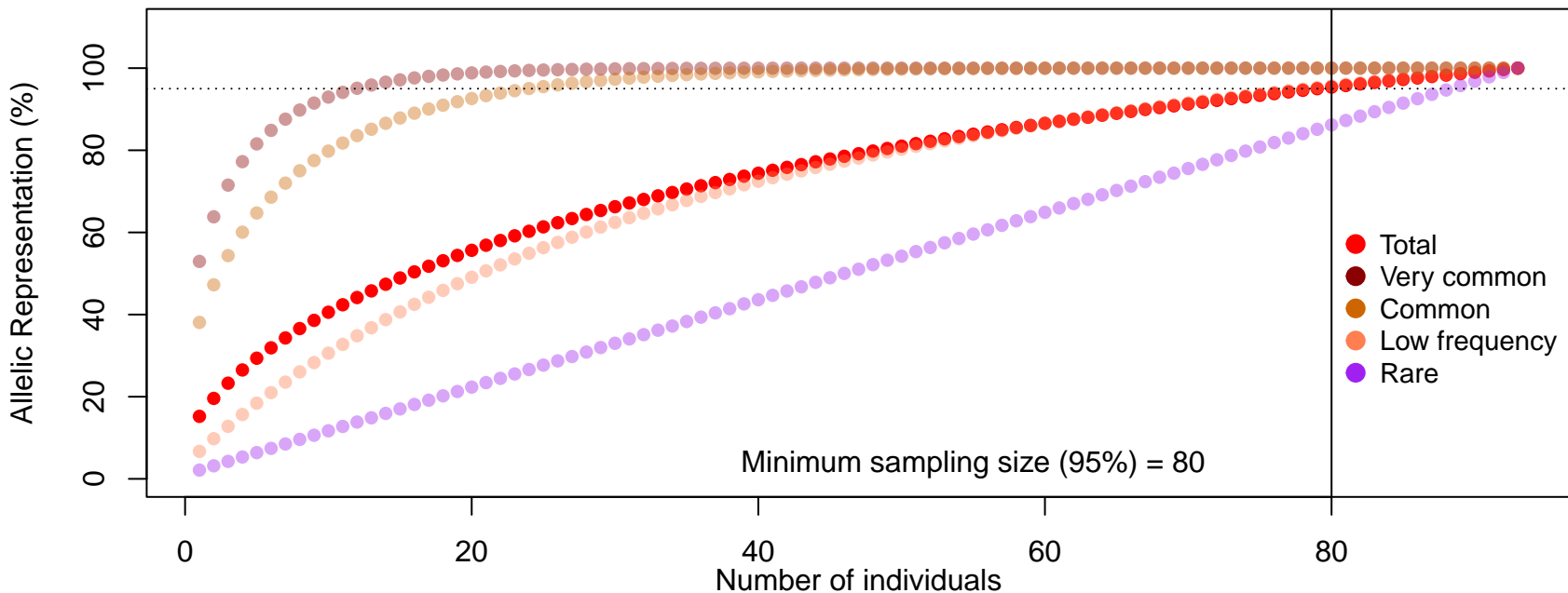

**QUBO, SNPs: De novo, R80 (Subset: 94 samples)**

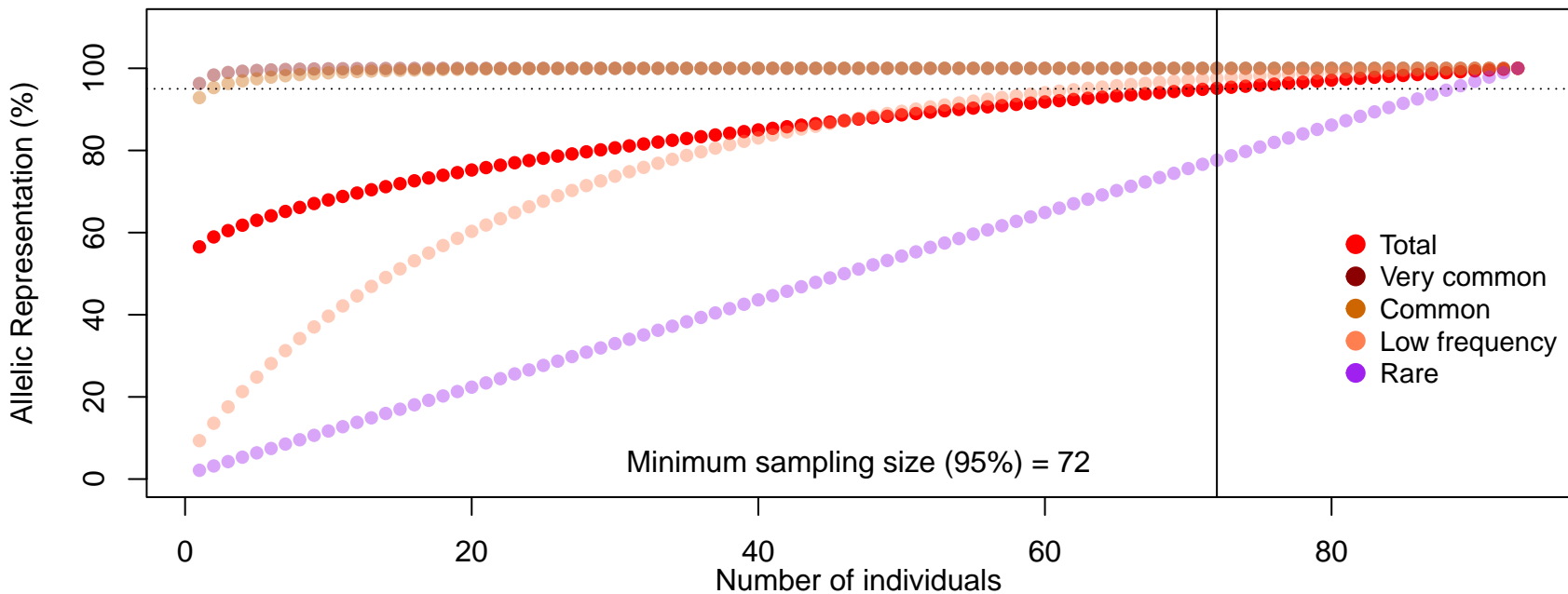

Supplement: Supplementary file 19 — Figure S19. [file EVA-17-e13650-s016.pdf]

**QUBO, SNPs: Reference, R0 (Complete: 95 samples)**

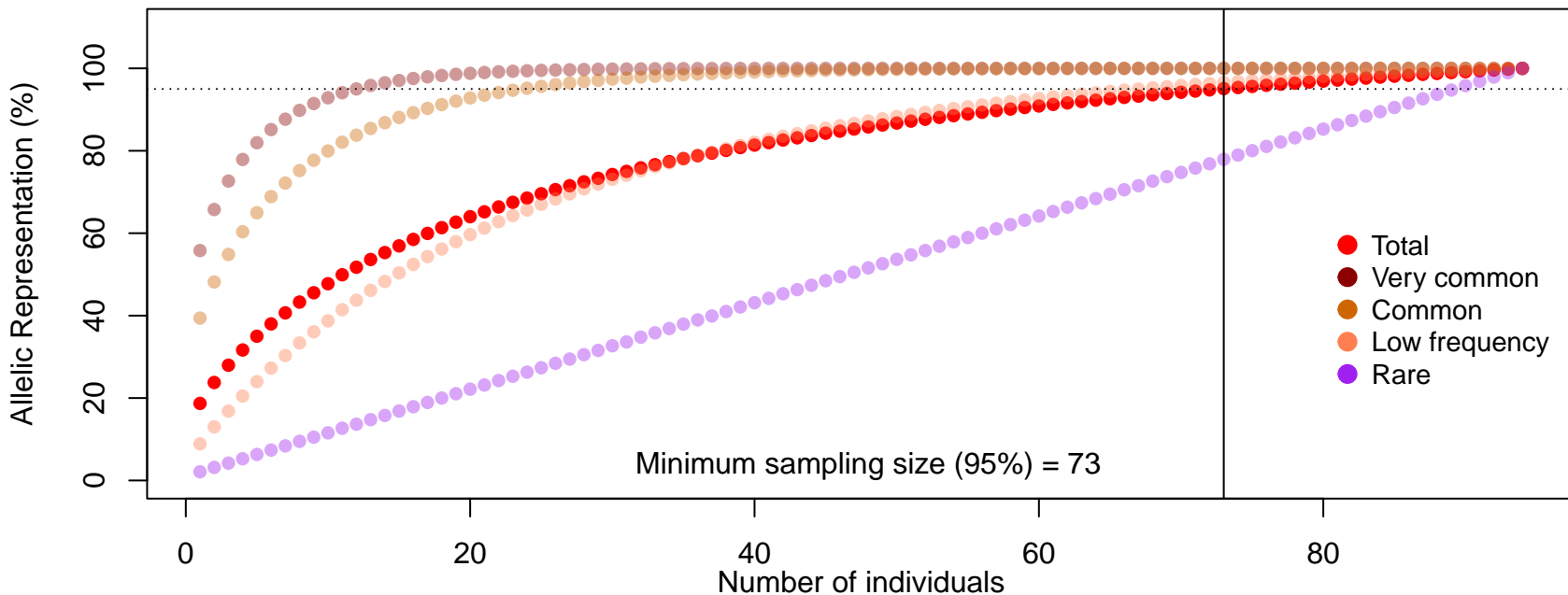

**QUBO, SNPs: Reference, R80 (Complete: 95 samples)**

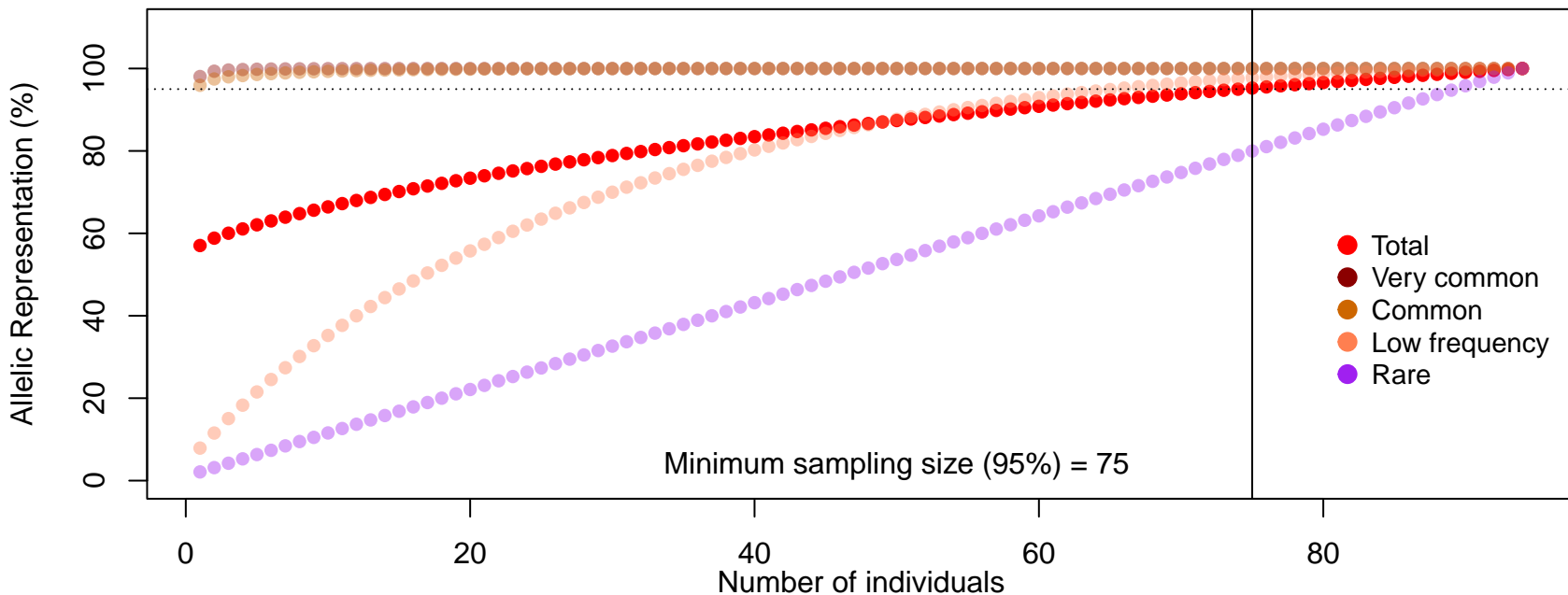

Supplement: Supplementary file 20 — Figure S20. [file EVA-17-e13650-s012.pdf]

**QUBO, SNPs: Reference, R0 (Subset: 94 samples)**

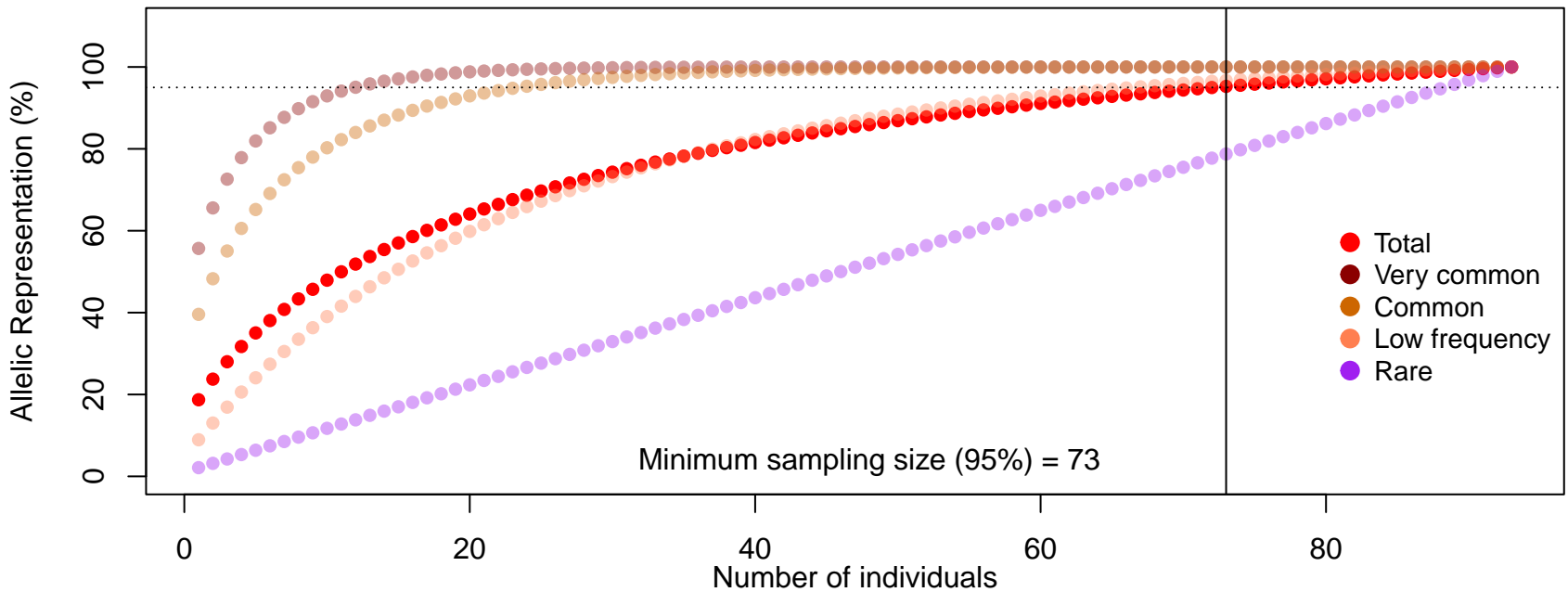

**QUBO, SNPs: Reference, R80 (Subset: 94 samples)**

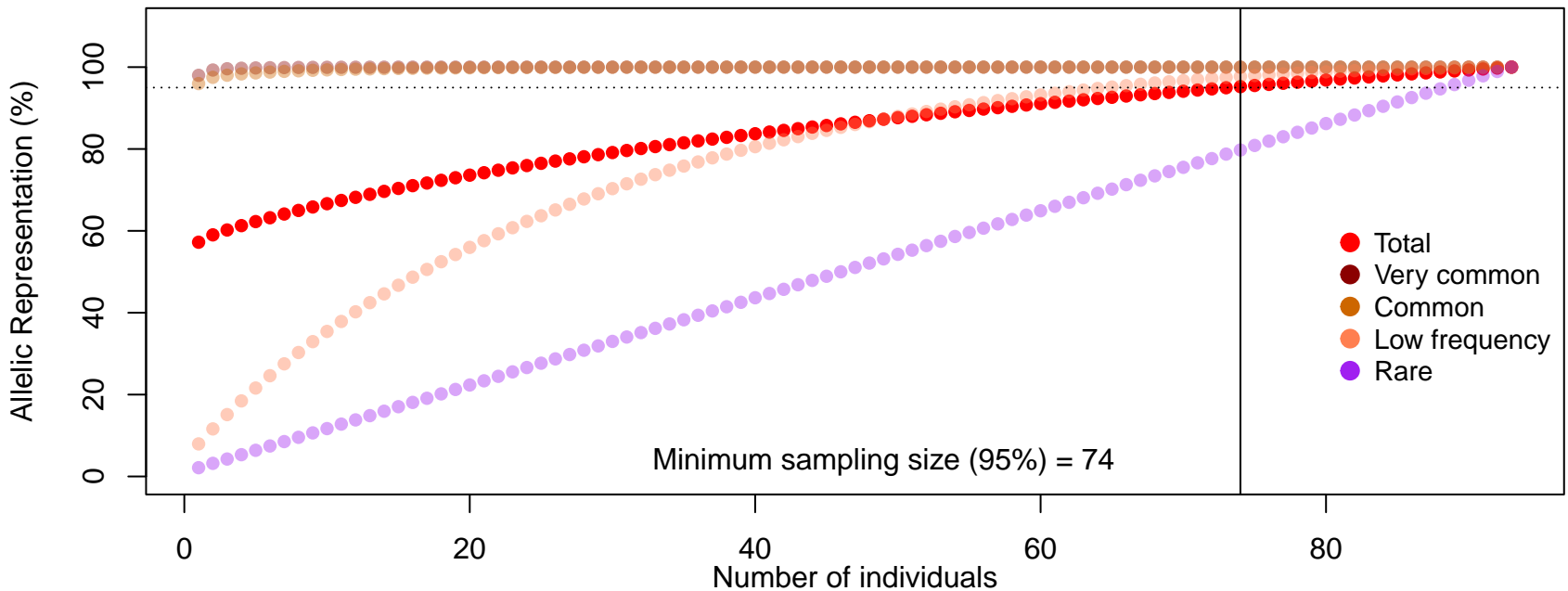

Supplement: Supplementary file 21 — Figure S21. [file EVA-17-e13650-s026.pdf]

QUAC: MSAT

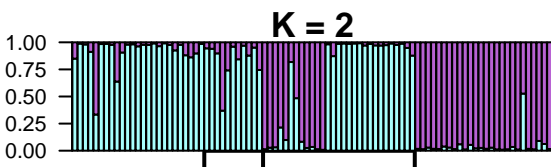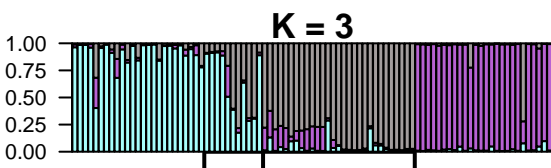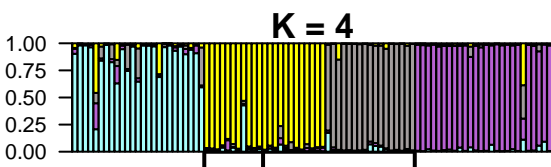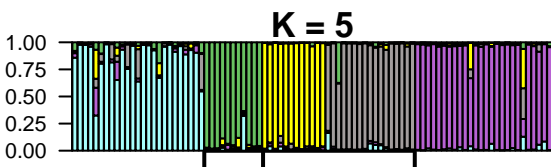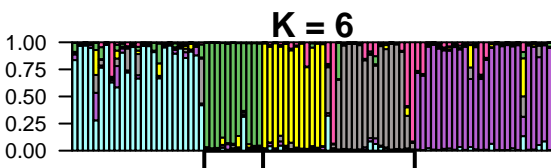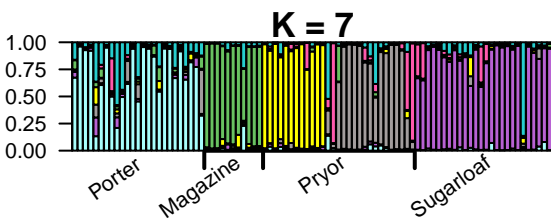

QUAC: SNP, De novo (R80)

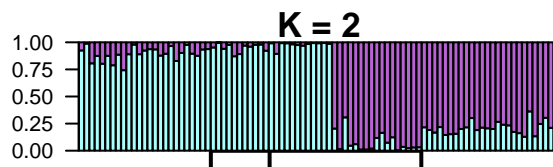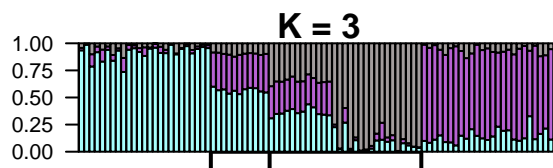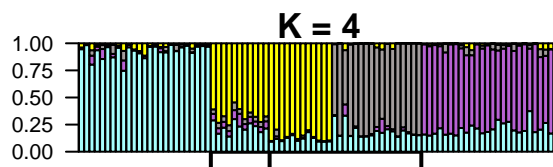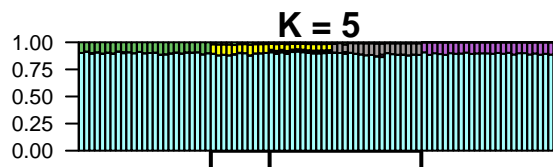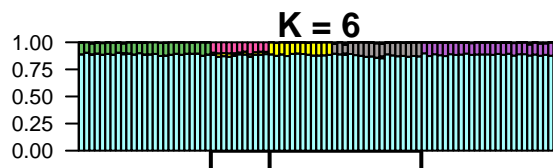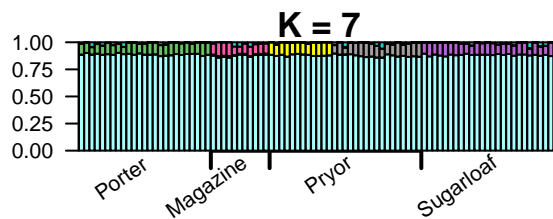

QUAC: SNP, Reference (R80)

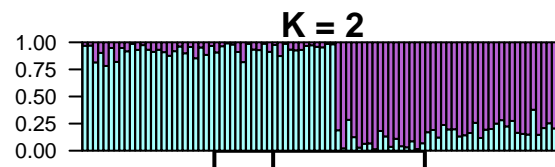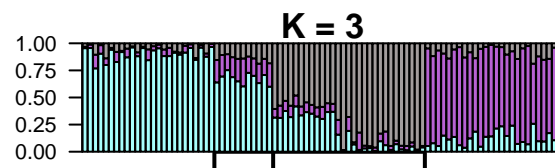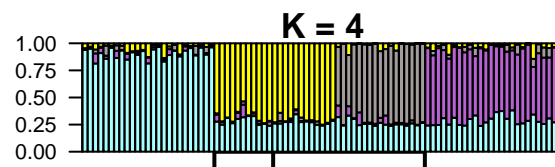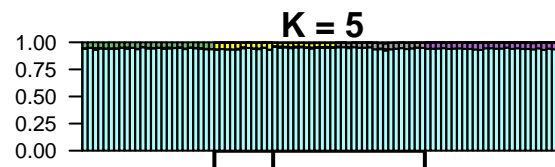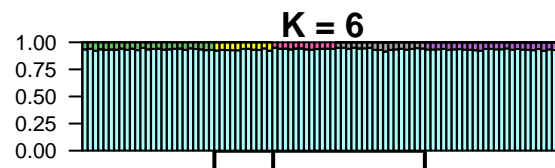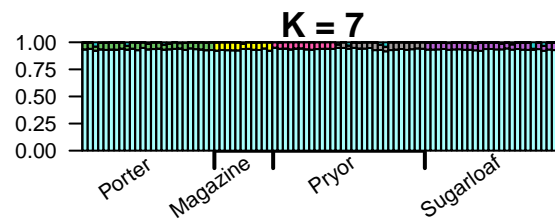

Supplement: Supplementary file 22 — Figure S22. [file EVA-17-e13650-s017.pdf]

QUBO: MSAT

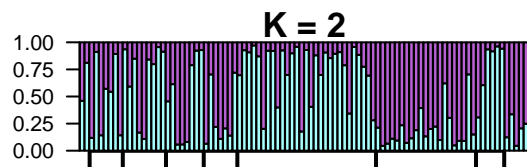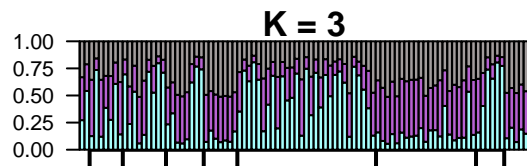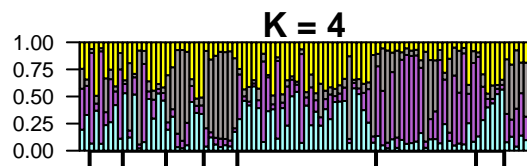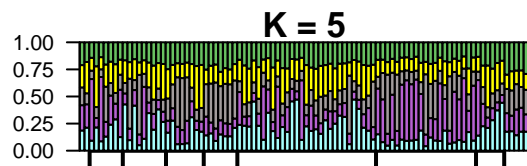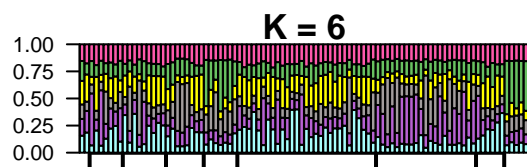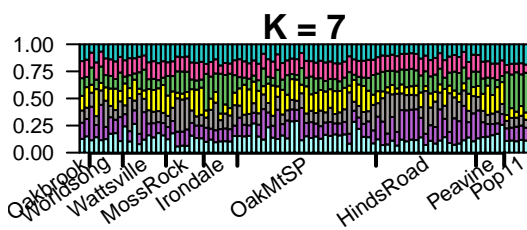

QUBO: SNP, De novo (R80)

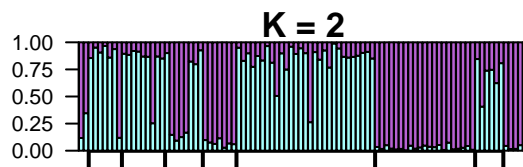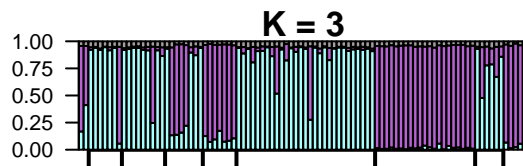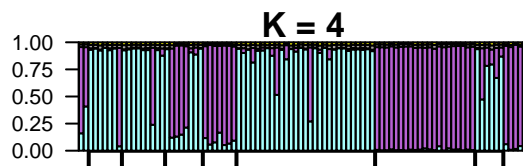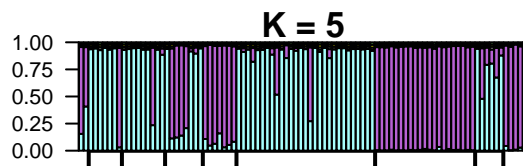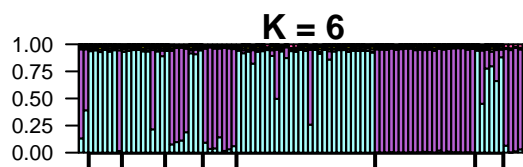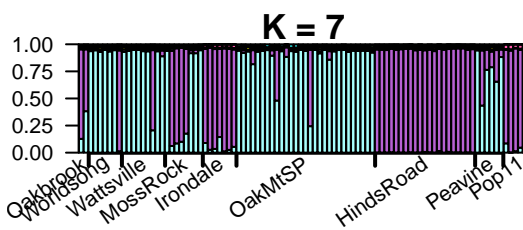

QUBO: SNP, Reference (R80)

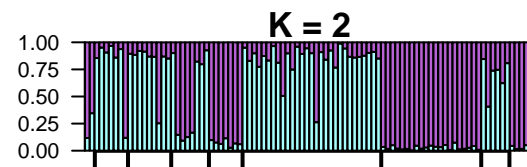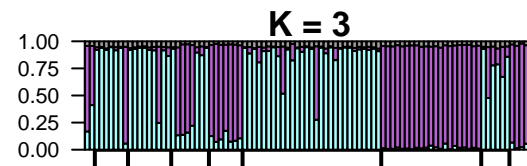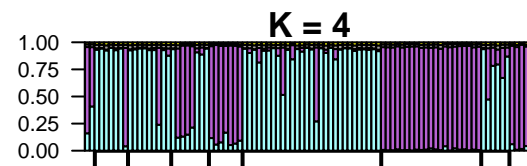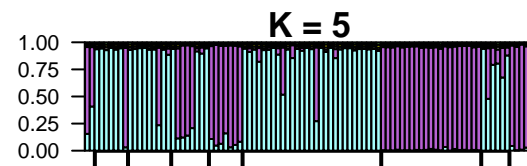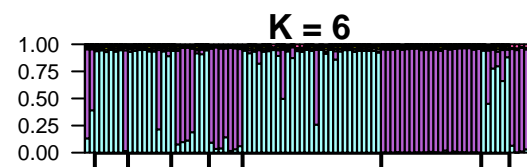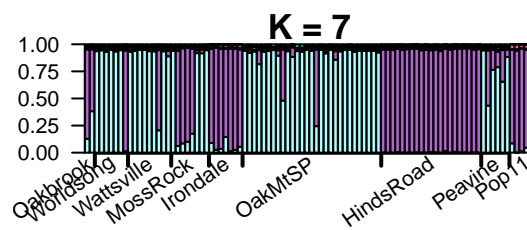

Supplement: Supplementary file 23 — Figure S23. [file EVA-17-e13650-s007.pdf]

**QUAC MSAT (Subset): K4**

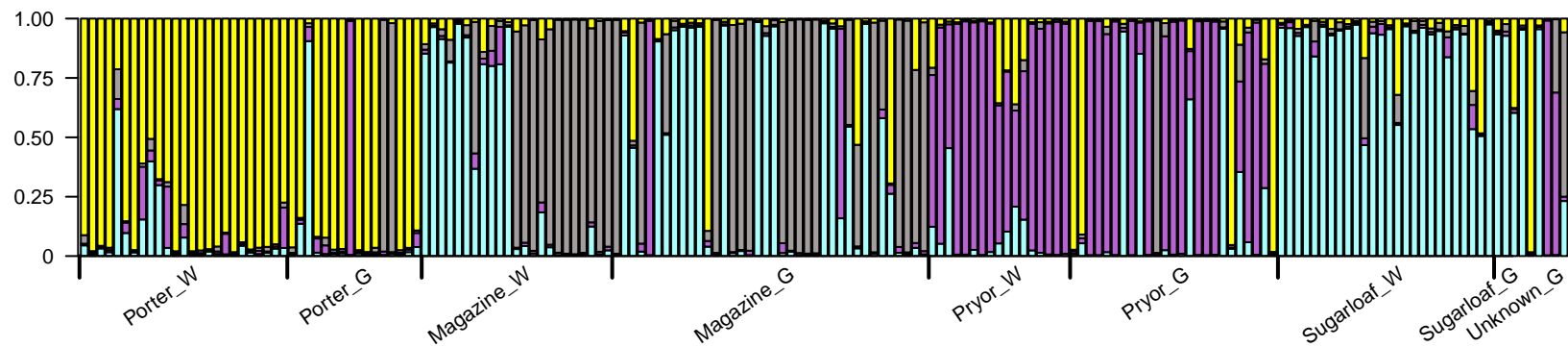

**QUAC SNP (Subset), De novo (R80): K4**

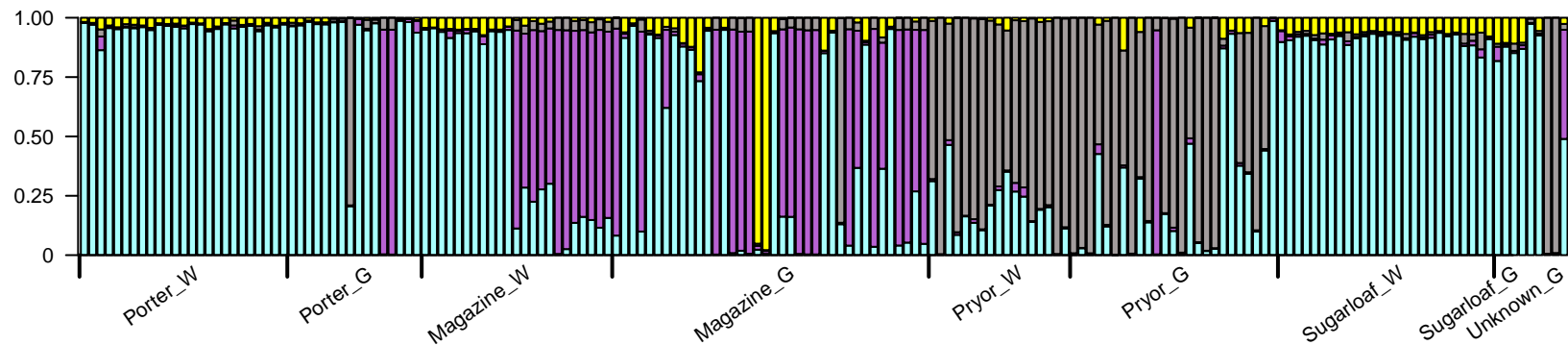

**QUAC SNP (Subset), Reference (R80): K4**

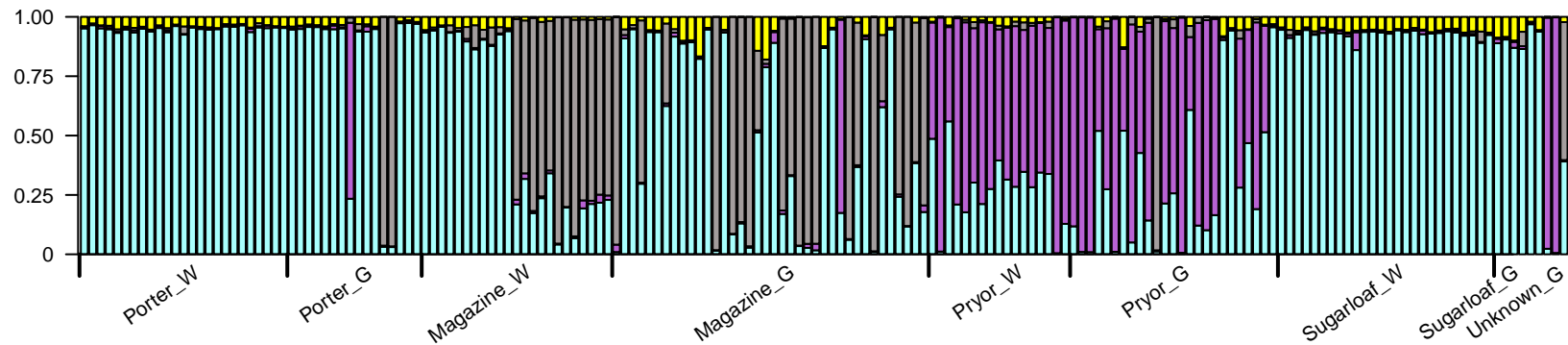

Supplement: Supplementary file 24 — Figure S24. [file EVA-17-e13650-s003.pdf]

QUAC MSAT: Subset Wild (K=4)

- Pryor
- Magazine
- Sugarloaf
- Porter

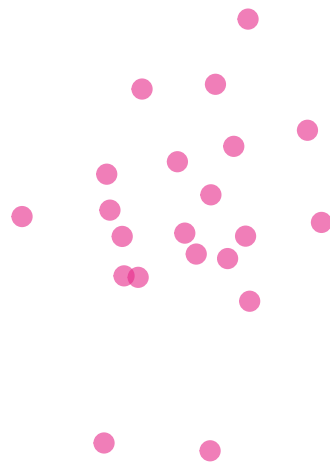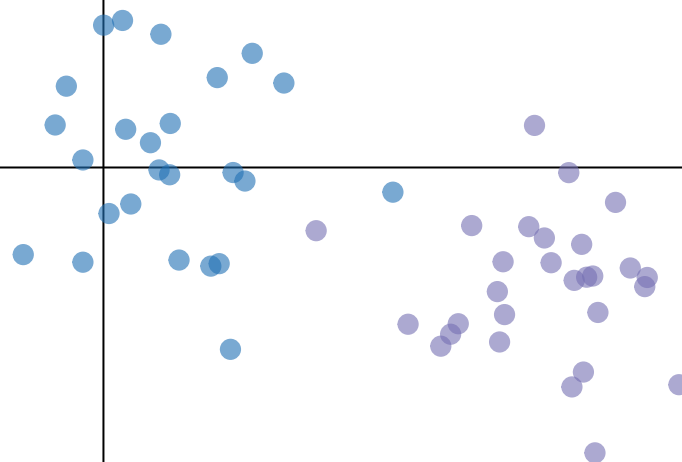

Supplement: Supplementary file 25 — Figure S25. [file EVA-17-e13650-s023.pdf]

QUAC SNP De novo (R80): Subset Wild (K=2)

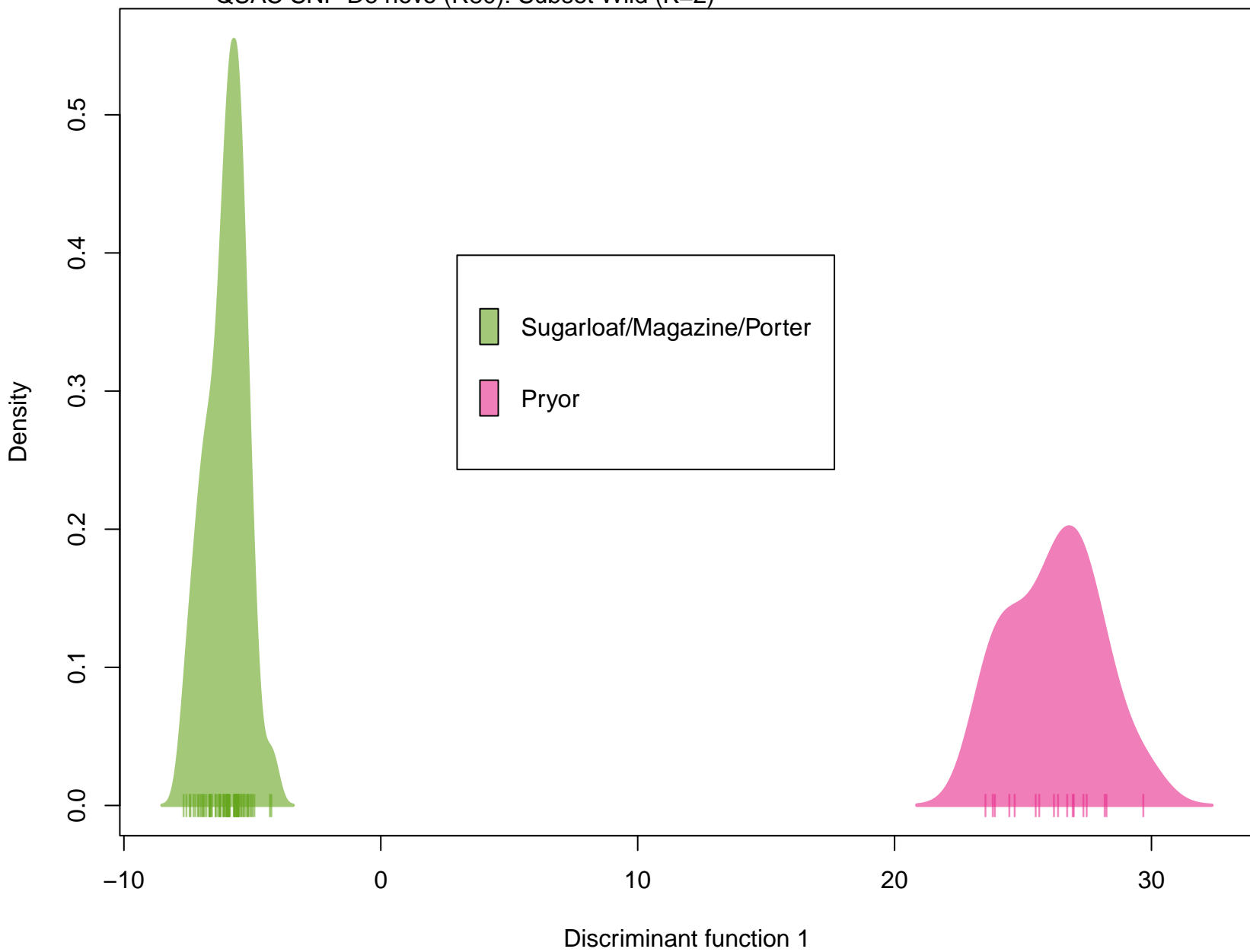

Supplement: Supplementary file 26 — Figure S26. [file EVA-17-e13650-s031.pdf]

QUAC SNP Reference (R80): Subset Wild (K=2)

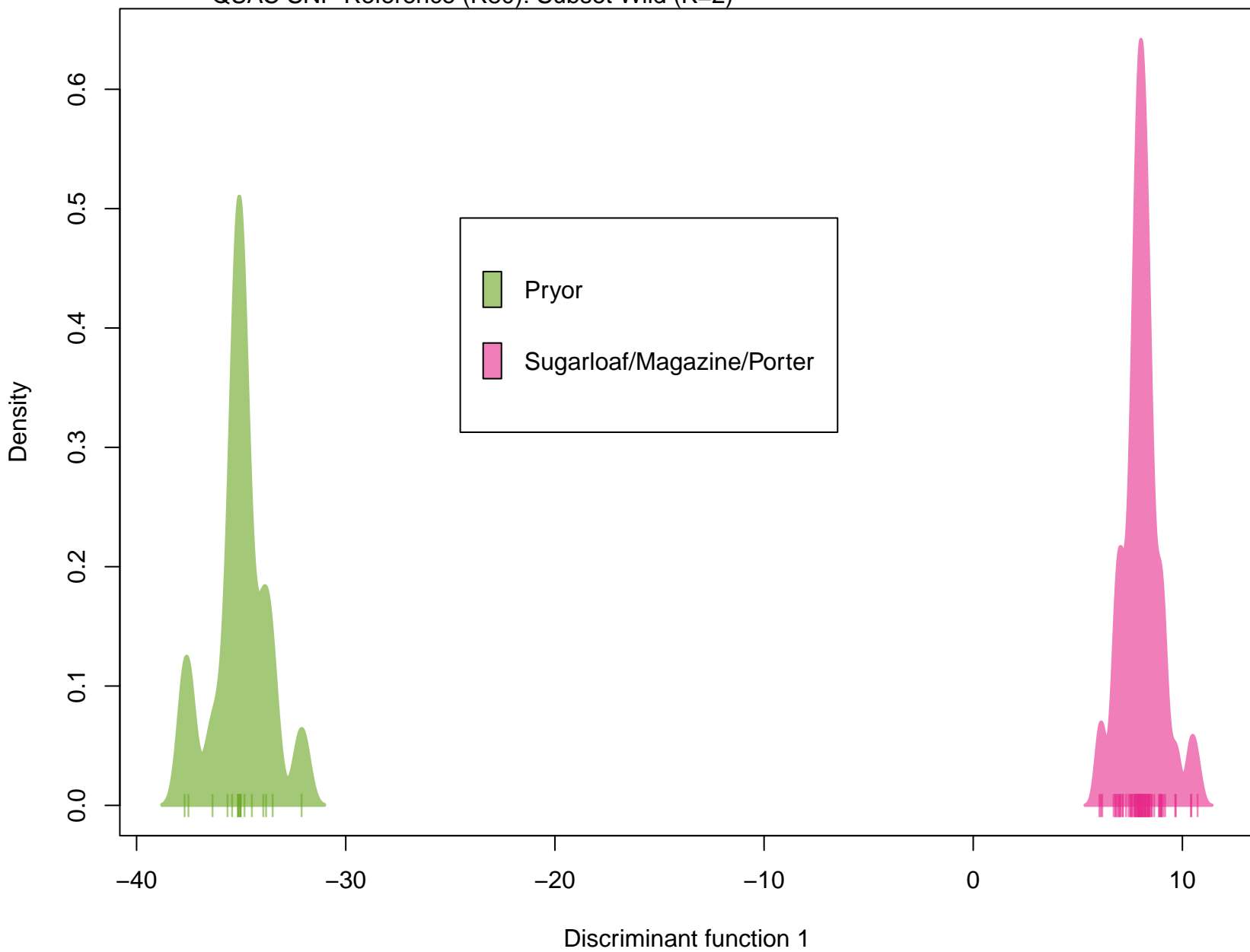

Supplement: Supplementary file 27 — Figure S27. [file EVA-17-e13650-s014.pdf]

QUBO SNP De novo (R80): Subset Wild (K=2)

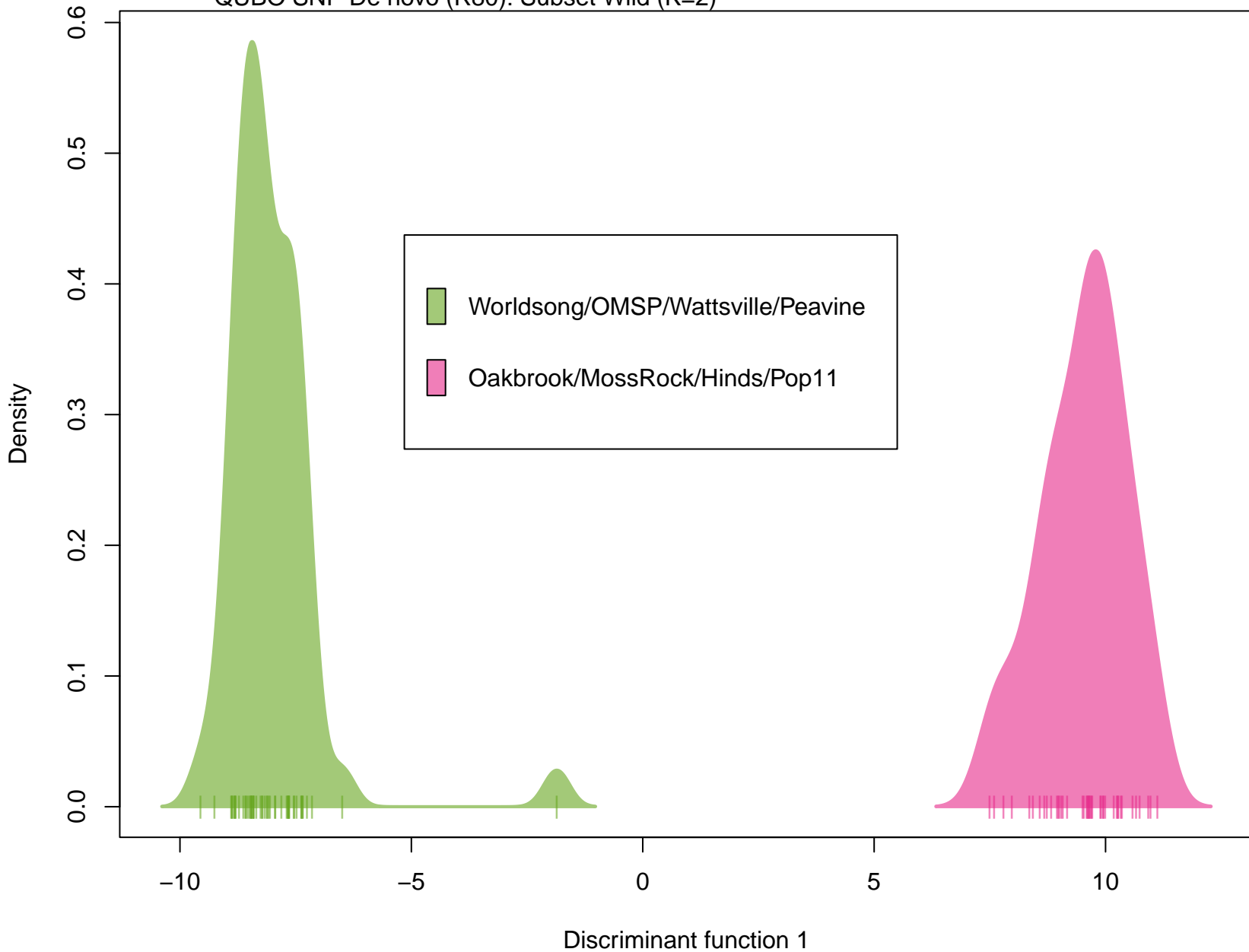

Supplement: Supplementary file 29 — Figure S29. [file EVA-17-e13650-s001.pdf]

QUBO SNP Reference (R80): Subset Wild (K=2)

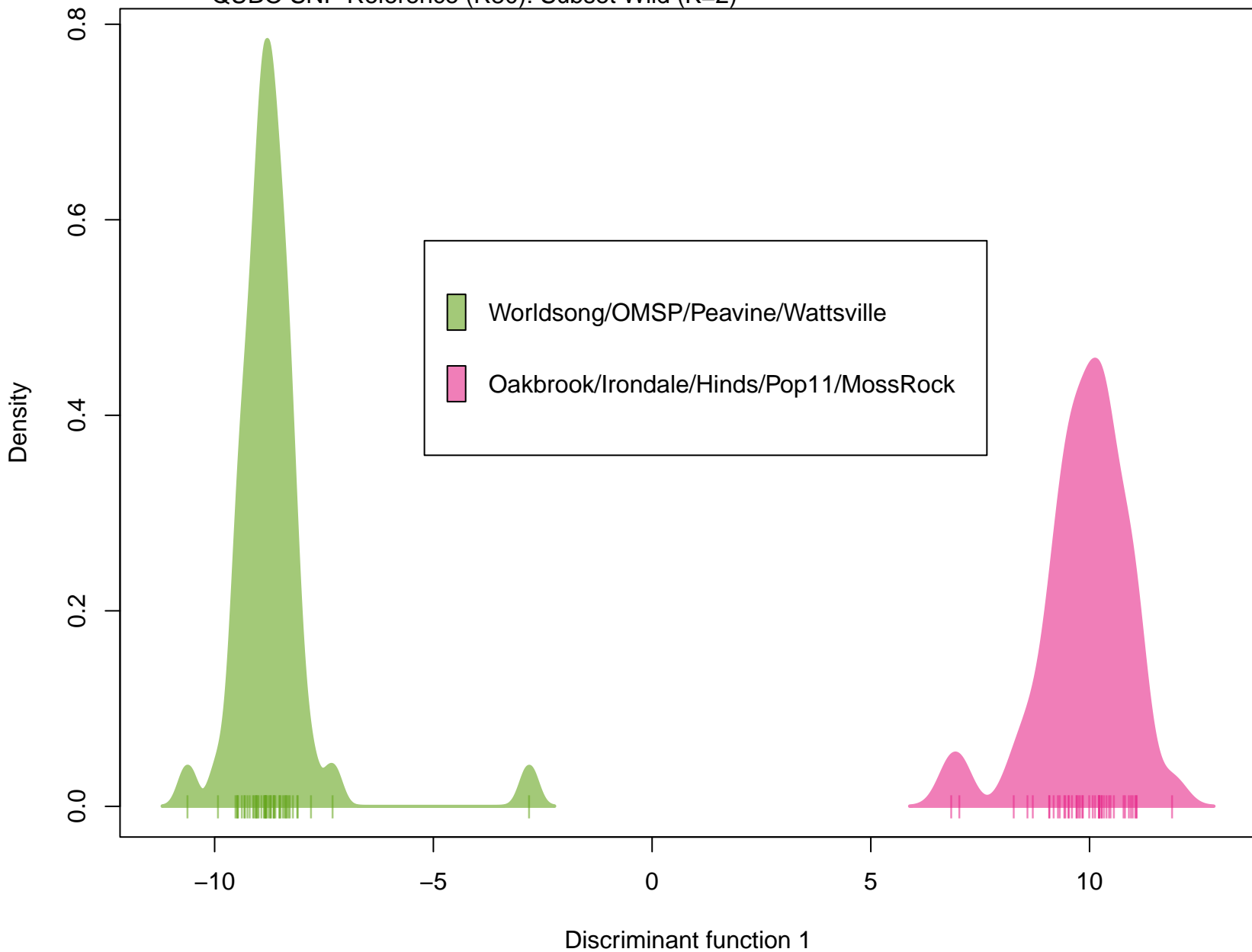

Supplement: Supplementary file 30 — Figure S30. [file EVA-17-e13650-s004.pdf]
