# Supplementary material for: Ex situ conservation of two rare oak species using microsatellite and SNP markers
Source: Evol Appl. 2024 Mar 22;17(3):e13650. doi: 10.1111/eva.13650 (PMC10960078; doi:10.1111/eva.13650)
Supplement: Supplementary file 3 — Figure S3. [file EVA-17-e13650-s009.pdf]

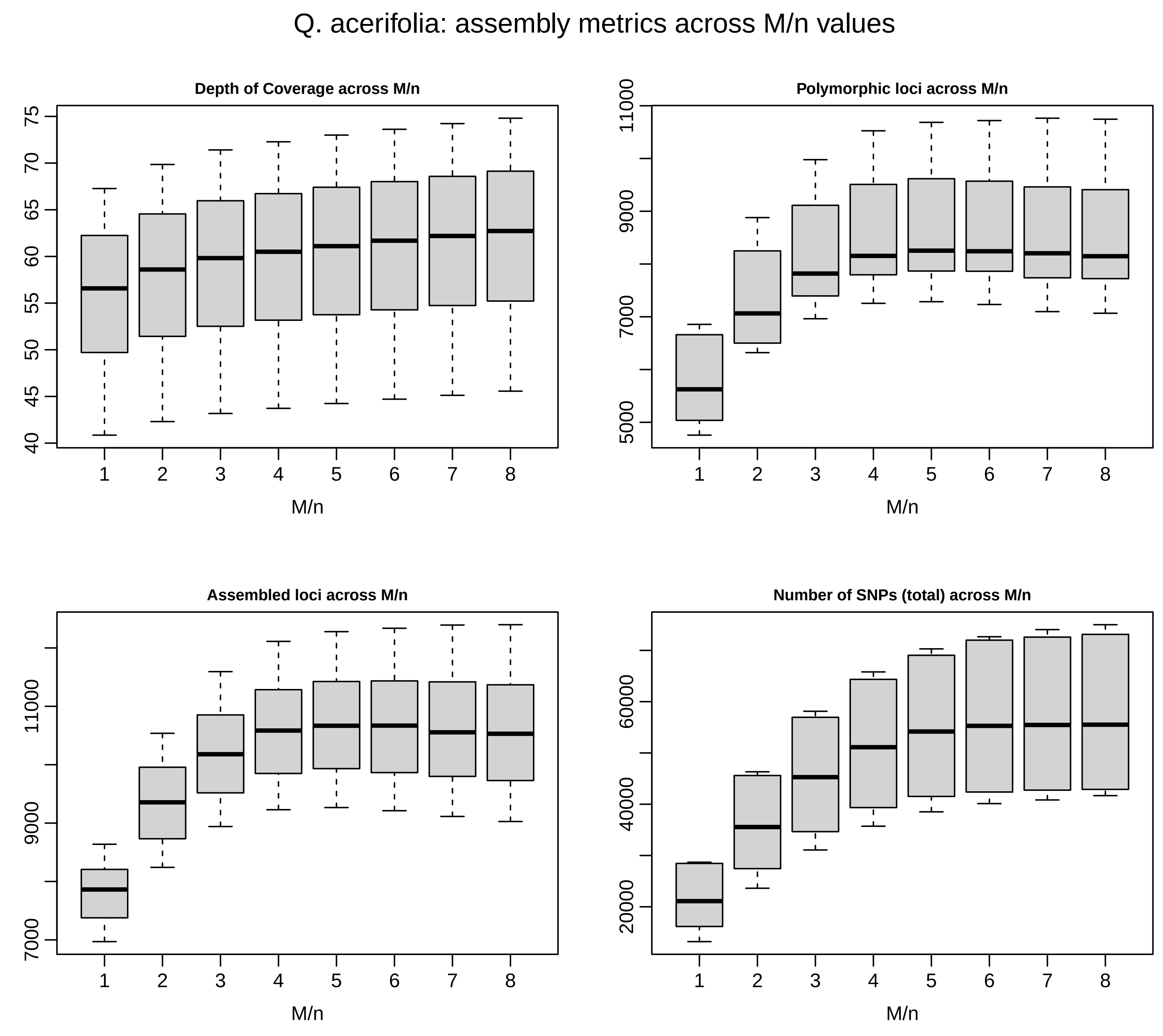
Q. acerifolia: assembly metrics across M/n values
Depth of Coverage across M/n
40
45
50
55
60
65
70
75
1 2 3 4 5 6 7 8
M/n
Polymorphic loci across M/n
5000
7000
9000
11000
1 2 3 4 5 6 7 8
M/n
Assembled loci across M/n
7000
9000
11000
1 2 3 4 5 6 7 8
M/n
Number of SNPs (total) across M/n
20000
40000
60000
1 2 3 4 5 6 7 8
M/n
