# Supplementary material for: Ex situ conservation of two rare oak species using microsatellite and SNP markers
Source: Evol Appl. 2024 Mar 22;17(3):e13650. doi: 10.1111/eva.13650 (PMC10960078; doi:10.1111/eva.13650)
Supplement: Supplementary file 28 — Figure S28. [file EVA-17-e13650-s022.pdf]

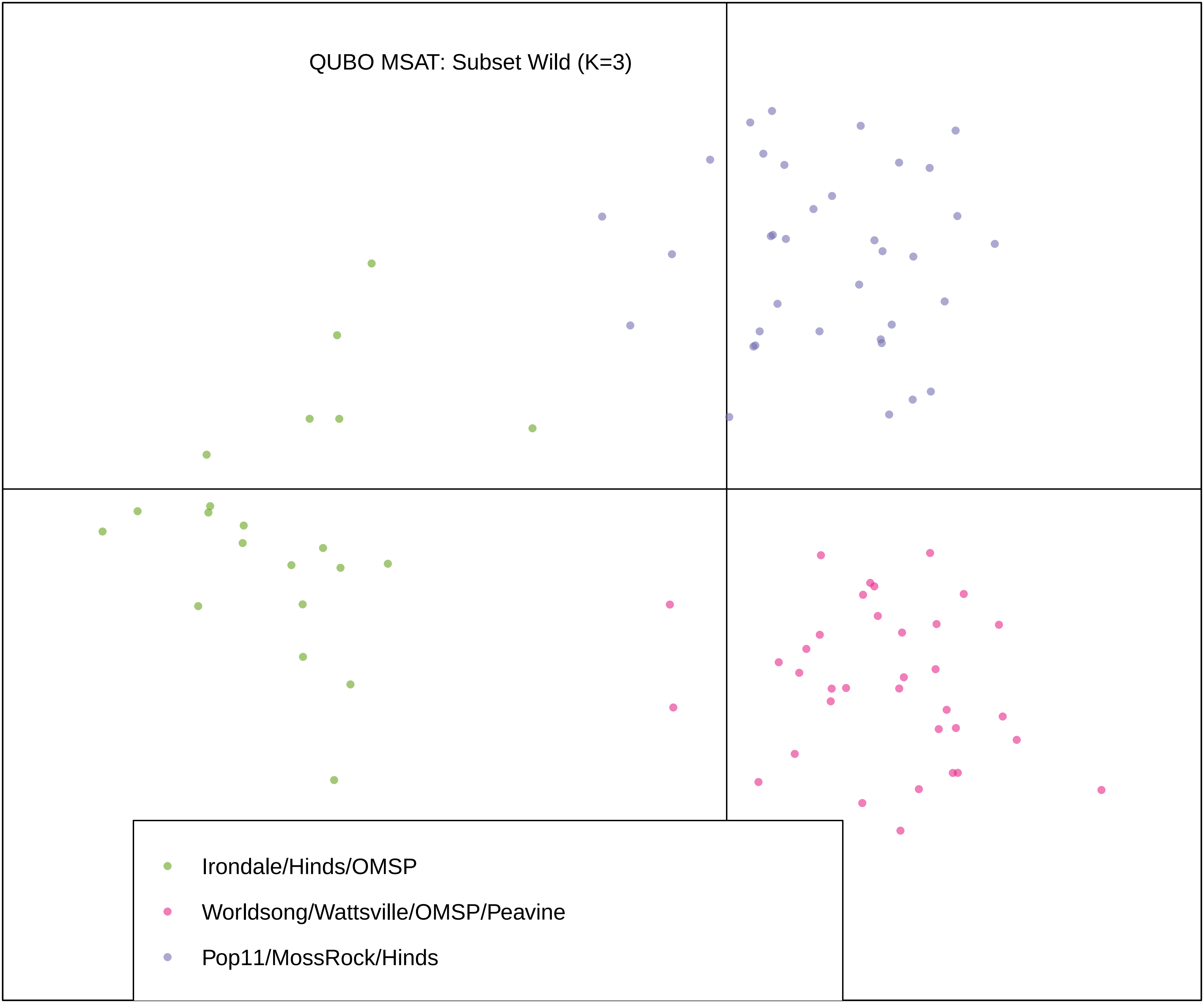
QUBO MSAT: Subset Wild (K=3)
Irondale/Hinds/OMSP
Worldsong/Wattsville/OMSP/Peavine
Pop11/MossRock/Hinds
