# Supplementary material for: Ex situ conservation of two rare oak species using microsatellite and SNP markers
Source: Evol Appl. 2024 Mar 22;17(3):e13650. doi: 10.1111/eva.13650 (PMC10960078; doi:10.1111/eva.13650)
Supplement: Supplementary file 31 — Data S1. [file EVA-17-e13650-s028.docx]

# Table of Contents - Supplement

Contents

[Table of Contents - Supplement 1](#_Toc154484703)

[Section 1. Methods 2](#_Toc154484704)

[1.1 Sampling 2](#_Toc154484705)

[1.2 Library preparation and sequencing 27](#_Toc154484706)

[1.3 Quality filtering 28](#_Toc154484707)

[1.4 Genotyping 28](#_Toc154484708)

[1.4.1 Microsatellites 28](#_Toc154484709)

[1.4.2 SNPs 32](#_Toc154484710)

[1.5 Filtering and population assignment 36](#_Toc154484711)

[1. 6 *Ex situ* conservation analyses 37](#_Toc154484712)

[1.7 Population clustering analyses 38](#_Toc154484713)

[1.7.1 STRUCTURE 38](#_Toc154484714)

[1.7.2 Discriminant Analysis of Principal Components 39](#_Toc154484715)

[Section 2. Results 41](#_Toc154484716)

[2.1](#_Toc154484718) *[Ex situ](#_Toc154484718)* [conservation 42](#_Toc154484718)

[2.1.2 Resampling 44](#_Toc154484719)

[2.2 Population clustering analyses 46](#_Toc154484720)

[2.2.1 STRUCTURE 46](#_Toc154484721)

[2.2.2 DAPC 48](#_Toc154484722)

[Section 3. References 49](#_Toc154484723)

# Section 1. Methods

## 1.1 Sampling

Tables S1 and S2 list *Q. acerifolia* (QUAC) and *Q. boyntonii* (QUBO) samples used in analyses, as well as which marker types were generated for each sample.

#####

| *Sample Name* | *Garden/Wild* | *Garden or Wild Population Name* | *Marker Study* |
| --- | --- | --- | --- |
| QUAC_G_SH-Q1485 | Garden | United States National Arboretum | SNP |
| QUAC_G_SH-Q1499 | Garden | Missouri Botanical Garden | MSAT/SNP |
| QUAC_G_SH-Q1500 | Garden | Missouri Botanical Garden | MSAT/SNP |
| QUAC_G_SH-Q1501 | Garden | Missouri Botanical Garden | MSAT/SNP |
| QUAC_G_SH-Q1502 | Garden | The Morton Arboretum | MSAT |
| QUAC_G_SH-Q1504 | Garden | Missouri Botanical Garden | SNP |
| QUAC_G_SH-Q1506 | Garden | The Morton Arboretum | MSAT/SNP |
| QUAC_G_SH-Q1507 | Garden | The Morton Arboretum | MSAT/SNP |
| QUAC_G_SH-Q1508 | Garden | The Morton Arboretum | SNP |
| QUAC_G_SH-Q1512 | Garden | Huntington Botanic Garden | MSAT |
| QUAC_G_SH-Q1513 | Garden | Huntington Botanic Garden | MSAT/SNP |
| QUAC_G_SH-Q1514 | Garden | Huntington Botanic Garden | MSAT/SNP |
| QUAC_G_SH-Q1515 | Garden | Huntington Botanic Garden | MSAT/SNP |
| QUAC_G_SH-Q1516 | Garden | Huntington Botanic Garden | MSAT/SNP |
| QUAC_G_SH-Q1517 | Garden | Huntington Botanic Garden | MSAT/SNP |
| QUAC_G_SH-Q1518 | Garden | Huntington Botanic Garden | MSAT/SNP |
| QUAC_G_SH-Q1519 | Garden | United States National Arboretum | MSAT/SNP |
| QUAC_G_SH-Q1520 | Garden | Huntington Botanic Garden | MSAT/SNP |
| QUAC_G_SH-Q1521 | Garden | Huntington Botanic Garden | MSAT |
| QUAC_G_SH-Q1523 | Garden | Arboretum Pouyouleix | MSAT/SNP |
| QUAC_G_SH-Q1525 | Garden | Arboretum Pouyouleix | MSAT/SNP |
| QUAC_G_SH-Q1526 | Garden | Denver Botanic Garden | MSAT/SNP |
| QUAC_G_SH-Q1527 | Garden | Denver Botanic Garden | MSAT |
| QUAC_G_SH-Q1528 | Garden | Denver Botanic Garden | MSAT/SNP |
| QUAC_G_SH-Q1529 | Garden | Denver Botanic Garden | MSAT |
| QUAC_G_SH-Q1530 | Garden | Denver Botanic Garden | MSAT |
| QUAC_G_SH-Q1531 | Garden | Denver Botanic Garden | MSAT/SNP |
| QUAC_G_SH-Q1532 | Garden | Denver Botanic Garden | MSAT |
| QUAC_G_SH-Q1533 | Garden | Denver Botanic Garden | MSAT |
| QUAC_G_SH-Q1534 | Garden | Denver Botanic Garden | MSAT |
| QUAC_G_SH-Q1535 | Garden | Denver Botanic Garden | MSAT |
| QUAC_G_SH-Q1536 | Garden | Denver Botanic Garden | MSAT/SNP |
| QUAC_G_SH-Q1537 | Garden | Denver Botanic Garden | MSAT |
| QUAC_G_SH-Q1538 | Garden | Denver Botanic Garden | MSAT |
| QUAC_G_SH-Q1539 | Garden | Denver Botanic Garden | MSAT |
| QUAC_G_SH-Q1540 | Garden | Denver Botanic Garden | MSAT |
| QUAC_G_SH-Q1541 | Garden | Denver Botanic Garden | MSAT |
| QUAC_G_SH-Q1542 | Garden | Denver Botanic Garden | MSAT |
| QUAC_G_SH-Q1543 | Garden | Denver Botanic Garden | MSAT |
| QUAC_G_SH-Q1544 | Garden | Denver Botanic Garden | MSAT |
| QUAC_G_SH-Q1545 | Garden | Denver Botanic Garden | MSAT |
| QUAC_G_SH-Q1546 | Garden | Denver Botanic Garden | MSAT |
| QUAC_G_SH-Q1547 | Garden | Denver Botanic Garden | MSAT |
| QUAC_G_SH-Q1548 | Garden | Denver Botanic Garden | MSAT |
| QUAC_G_SH-Q1549 | Garden | Denver Botanic Garden | MSAT |
| QUAC_G_SH-Q1550 | Garden | Denver Botanic Garden | MSAT/SNP |
| **QUAC_G_SH-Q1550_rep** | **Garden** | **Denver Botanic Gardens** | **SNP** |
| QUAC_G_SH-Q1551 | Garden | Denver Botanic Garden | MSAT/SNP |
| QUAC_G_SH-Q1552 | Garden | Denver Botanic Garden | MSAT |
| QUAC_G_SH-Q1553 | Garden | Denver Botanic Garden | MSAT/SNP |
| QUAC_G_SH-Q1554 | Garden | Denver Botanic Garden | MSAT |
| QUAC_G_SH-Q1555 | Garden | Arnold Arboretum | MSAT/SNP |
| QUAC_G_SH-Q1556 | Garden | Arnold Arboretum | MSAT/SNP |
| QUAC_G_SH-Q1557 | Garden | Arnold Arboretum | MSAT |
| QUAC_G_SH-Q1558 | Garden | Arnold Arboretum | MSAT |
| QUAC_G_SH-Q1559 | Garden | Arnold Arboretum | MSAT/SNP |
| QUAC_G_SH-Q1560 | Garden | Arnold Arboretum | MSAT |
| QUAC_G_SH-Q1561 | Garden | Arnold Arboretum | MSAT |
| QUAC_G_SH-Q1562 | Garden | Arnold Arboretum | MSAT |
| QUAC_G_SH-Q1563 | Garden | Arnold Arboretum | SNP |
| QUAC_G_SH-Q1564 | Garden | Arnold Arboretum | MSAT |
| QUAC_G_SH-Q1565 | Garden | Arnold Arboretum | MSAT/SNP |
| QUAC_G_SH-Q1566 | Garden | Arnold Arboretum | MSAT/SNP |
| QUAC_G_SH-Q1567 | Garden | Arnold Arboretum | MSAT |
| QUAC_G_SH-Q1568 | Garden | Arnold Arboretum | MSAT |
| QUAC_G_SH-Q1569 | Garden | Arnold Arboretum | MSAT/SNP |
| QUAC_G_SH-Q1570 | Garden | Arnold Arboretum | MSAT |
| QUAC_G_SH-Q1571 | Garden | Arnold Arboretum | MSAT |
| QUAC_G_SH-Q1572 | Garden | Arnold Arboretum | MSAT |
| QUAC_G_SH-Q1573 | Garden | Arnold Arboretum | MSAT |
| QUAC_G_SH-Q1574 | Garden | Arnold Arboretum | MSAT/SNP |
| QUAC_G_SH-Q1575 | Garden | Arnold Arboretum | MSAT |
| QUAC_G_SH-Q1576 | Garden | Arnold Arboretum | MSAT/SNP |
| QUAC_G_SH-Q1577 | Garden | Arnold Arboretum | MSAT/SNP |
| **QUAC_G_DUP_SH-Q1577** | **Garden** | **Arnold Arboretum** | **SNP** |
| QUAC_G_SH-Q1578 | Garden | The Morton Arboretum | MSAT |
| QUAC_G_SH-Q1579 | Garden | The Morton Arboretum | MSAT |
| QUAC_G_SH-Q1580 | Garden | The Morton Arboretum | MSAT |
| QUAC_G_SH-Q1581 | Garden | The Morton Arboretum | MSAT |
| QUAC_G_SH-Q1582 | Garden | The Morton Arboretum | MSAT |
| QUAC_G_SH-Q1583 | Garden | The Morton Arboretum | MSAT |
| QUAC_G_SH-Q1584 | Garden | The Morton Arboretum | MSAT |
| QUAC_G_SH-Q1585 | Garden | The Morton Arboretum | MSAT |
| QUAC_G_SH-Q1586 | Garden | The Morton Arboretum | MSAT |
| QUAC_G_SH-Q1587 | Garden | The Morton Arboretum | MSAT |
| QUAC_G_SH-Q1588 | Garden | The Morton Arboretum | MSAT/SNP |
| QUAC_G_SH-Q1589 | Garden | The Morton Arboretum | MSAT |
| QUAC_G_SH-Q1590 | Garden | The Morton Arboretum | MSAT/SNP |
| QUAC_G_SH-Q1591 | Garden | The Morton Arboretum | MSAT |
| QUAC_G_SH-Q1592 | Garden | The Morton Arboretum | MSAT/SNP |
| QUAC_G_SH-Q1593 | Garden | The Morton Arboretum | MSAT |
| QUAC_G_SH-Q1594 | Garden | The Morton Arboretum | MSAT |
| QUAC_G_SH-Q1595 | Garden | The Morton Arboretum | MSAT |
| QUAC_G_SH-Q1596 | Garden | The Morton Arboretum | MSAT |
| QUAC_G_SH-Q1597 | Garden | The Morton Arboretum | MSAT |
| QUAC_G_SH-Q1598 | Garden | The Morton Arboretum | MSAT |
| QUAC_G_SH-Q1599 | Garden | The Morton Arboretum | MSAT |
| QUAC_G_SH-Q1600 | Garden | The Morton Arboretum | MSAT |
| QUAC_G_SH-Q1601 | Garden | The Morton Arboretum | MSAT |
| QUAC_G_SH-Q1602 | Garden | The Morton Arboretum | MSAT |
| QUAC_G_SH-Q1603 | Garden | The Morton Arboretum | MSAT |
| QUAC_G_SH-Q1604 | Garden | The Morton Arboretum | MSAT |
| QUAC_G_SH-Q1605 | Garden | The Morton Arboretum | MSAT |
| QUAC_G_SH-Q1606 | Garden | The Morton Arboretum | MSAT |
| QUAC_G_SH-Q1607 | Garden | The Morton Arboretum | MSAT |
| QUAC_G_SH-Q1608 | Garden | The Morton Arboretum | MSAT |
| QUAC_G_SH-Q1609 | Garden | The Morton Arboretum | MSAT |
| QUAC_G_SH-Q1610 | Garden | The Morton Arboretum | MSAT |
| QUAC_G_SH-Q1611 | Garden | The Morton Arboretum | MSAT |
| QUAC_G_SH-Q1612 | Garden | The Morton Arboretum | MSAT |
| QUAC_G_SH-Q1613 | Garden | The Morton Arboretum | MSAT |
| QUAC_G_SH-Q1614 | Garden | The Morton Arboretum | MSAT |
| QUAC_G_SH-Q1615 | Garden | The Morton Arboretum | MSAT/SNP |
| QUAC_G_SH-Q1616 | Garden | The Morton Arboretum | MSAT |
| QUAC_G_SH-Q1617 | Garden | The Morton Arboretum | MSAT |
| QUAC_G_SH-Q1618 | Garden | The Morton Arboretum | MSAT/SNP |
| QUAC_G_SH-Q1619 | Garden | The Morton Arboretum | MSAT |
| QUAC_G_SH-Q1620 | Garden | The Morton Arboretum | MSAT |
| QUAC_G_SH-Q1621 | Garden | The Morton Arboretum | MSAT/SNP |
| QUAC_G_SH-Q1622 | Garden | The Morton Arboretum | MSAT |
| QUAC_G_SH-Q1623 | Garden | The Morton Arboretum | MSAT |
| QUAC_G_SH-Q1624 | Garden | The Morton Arboretum | MSAT |
| QUAC_G_SH-Q1625 | Garden | The Morton Arboretum | MSAT |
| QUAC_G_SH-Q1626 | Garden | The Morton Arboretum | MSAT |
| QUAC_G_SH-Q1627 | Garden | The Morton Arboretum | MSAT |
| QUAC_G_SH-Q1628 | Garden | The Morton Arboretum | MSAT/SNP |
| QUAC_G_SH-Q1629 | Garden | The Morton Arboretum | MSAT |
| QUAC_G_SH-Q1630 | Garden | The Morton Arboretum | MSAT |
| QUAC_G_SH-Q1631 | Garden | The Morton Arboretum | MSAT |
| QUAC_G_SH-Q1632 | Garden | The Morton Arboretum | MSAT/SNP |
| QUAC_G_SH-Q1633 | Garden | The Morton Arboretum | MSAT |
| QUAC_G_SH-Q1635 | Garden | The Morton Arboretum | MSAT |
| QUAC_G_SH-Q1636 | Garden | The Morton Arboretum | MSAT |
| QUAC_G_SH-Q1637 | Garden | The Morton Arboretum | MSAT |
| QUAC_G_SH-Q1638 | Garden | The Morton Arboretum | MSAT |
| QUAC_G_SH-Q1639 | Garden | The Morton Arboretum | MSAT |
| QUAC_G_SH-Q1640 | Garden | The Morton Arboretum | MSAT |
| QUAC_G_SH-Q1641 | Garden | The Morton Arboretum | MSAT |
| QUAC_G_SH-Q1642 | Garden | The Morton Arboretum | MSAT |
| QUAC_G_SH-Q1643 | Garden | The Morton Arboretum | MSAT |
| QUAC_G_SH-Q1644 | Garden | The Morton Arboretum | MSAT |
| QUAC_G_SH-Q1645 | Garden | The Morton Arboretum | MSAT |
| QUAC_G_SH-Q1646 | Garden | The Morton Arboretum | MSAT |
| QUAC_G_SH-Q1647 | Garden | The Morton Arboretum | MSAT |
| QUAC_G_SH-Q1648 | Garden | The Morton Arboretum | MSAT |
| QUAC_G_SH-Q1649 | Garden | The Morton Arboretum | MSAT |
| QUAC_G_SH-Q1650 | Garden | The Morton Arboretum | MSAT |
| QUAC_G_SH-Q1651 | Garden | The Morton Arboretum | MSAT |
| QUAC_G_SH-Q1652 | Garden | The Morton Arboretum | MSAT |
| QUAC_G_SH-Q1654 | Garden | The Morton Arboretum | MSAT |
| QUAC_G_SH-Q1655 | Garden | The Morton Arboretum | MSAT |
| QUAC_G_SH-Q1656 | Garden | The Morton Arboretum | MSAT |
| QUAC_G_SH-Q1657 | Garden | The Morton Arboretum | MSAT |
| QUAC_G_SH-Q1658 | Garden | The Morton Arboretum | MSAT |
| QUAC_G_SH-Q1659 | Garden | The Morton Arboretum | MSAT |
| QUAC_G_SH-Q1660 | Garden | The Morton Arboretum | MSAT |
| QUAC_G_SH-Q1661 | Garden | The Morton Arboretum | MSAT |
| QUAC_G_SH-Q1662 | Garden | The Morton Arboretum | MSAT |
| QUAC_G_SH-Q1663 | Garden | The Morton Arboretum | MSAT |
| QUAC_G_SH-Q1664 | Garden | The Morton Arboretum | MSAT |
| QUAC_G_SH-Q1665 | Garden | The Morton Arboretum | MSAT |
| QUAC_G_SH-Q1666 | Garden | The Morton Arboretum | MSAT |
| QUAC_G_SH-Q1667 | Garden | The Morton Arboretum | MSAT |
| QUAC_G_SH-Q1668 | Garden | The Morton Arboretum | MSAT/SNP |
| QUAC_G_SH-Q1669 | Garden | The Morton Arboretum | MSAT |
| QUAC_G_SH-Q1670 | Garden | The Morton Arboretum | MSAT |
| QUAC_G_SH-Q1671 | Garden | The Morton Arboretum | MSAT/SNP |
| QUAC_G_SH-Q1672 | Garden | The Morton Arboretum | MSAT |
| QUAC_G_SH-Q1673 | Garden | The Morton Arboretum | MSAT/SNP |
| QUAC_G_SH-Q1674 | Garden | The Morton Arboretum | MSAT |
| QUAC_G_SH-Q1675 | Garden | The Morton Arboretum | MSAT |
| QUAC_G_SH-Q1676 | Garden | The Morton Arboretum | MSAT |
| QUAC_G_SH-Q1677 | Garden | The Morton Arboretum | MSAT |
| QUAC_G_SH-Q1678 | Garden | The Morton Arboretum | MSAT |
| QUAC_G_SH-Q1679 | Garden | The Morton Arboretum | MSAT |
| QUAC_G_SH-Q1680 | Garden | The Morton Arboretum | MSAT |
| QUAC_G_SH-Q1681 | Garden | The Morton Arboretum | MSAT |
| QUAC_G_SH-Q1682 | Garden | The Morton Arboretum | MSAT |
| QUAC_G_SH-Q1683 | Garden | The Morton Arboretum | MSAT |
| QUAC_G_SH-Q1684 | Garden | The Morton Arboretum | MSAT |
| QUAC_G_SH-Q1685 | Garden | The Morton Arboretum | MSAT |
| QUAC_G_SH-Q1686 | Garden | The Morton Arboretum | MSAT |
| QUAC_G_SH-Q1687 | Garden | The Morton Arboretum | MSAT |
| QUAC_G_SH-Q1688 | Garden | The Morton Arboretum | MSAT |
| QUAC_G_SH-Q1689 | Garden | The Morton Arboretum | MSAT |
| QUAC_G_SH-Q1690 | Garden | The Morton Arboretum | MSAT/SNP |
| QUAC_G_SH-Q1691 | Garden | The Morton Arboretum | MSAT |
| QUAC_G_SH-Q1692 | Garden | The Morton Arboretum | MSAT/SNP |
| QUAC_G_SH-Q1693 | Garden | The Morton Arboretum | MSAT |
| QUAC_G_SH-Q1696 | Garden | The Morton Arboretum | MSAT |
| QUAC_G_SH-Q1697 | Garden | The Morton Arboretum | MSAT |
| QUAC_G_SH-Q1698 | Garden | The Morton Arboretum | MSAT |
| QUAC_G_SH-Q1699 | Garden | The Morton Arboretum | MSAT/SNP |
| QUAC_G_SH-Q1700 | Garden | The Morton Arboretum | MSAT |
| QUAC_G_SH-Q1701 | Garden | The Morton Arboretum | MSAT |
| QUAC_G_SH-Q1702 | Garden | The Morton Arboretum | MSAT |
| QUAC_G_SH-Q1705 | Garden | The Morton Arboretum | MSAT |
| QUAC_G_SH-Q1706 | Garden | The Morton Arboretum | MSAT |
| QUAC_G_SH-Q1707 | Garden | The Morton Arboretum | MSAT |
| QUAC_G_SH-Q1708 | Garden | The Morton Arboretum | MSAT |
| QUAC_G_SH-Q1709 | Garden | The Morton Arboretum | MSAT |
| QUAC_G_SH-Q1710 | Garden | The Morton Arboretum | MSAT |
| QUAC_G_SH-Q1711 | Garden | The Morton Arboretum | MSAT |
| QUAC_G_SH-Q1712 | Garden | The Morton Arboretum | MSAT |
| QUAC_G_SH-Q1713 | Garden | The Morton Arboretum | MSAT |
| QUAC_G_SH-Q1714 | Garden | The Morton Arboretum | MSAT |
| QUAC_G_SH-Q1716 | Garden | The Morton Arboretum | MSAT/SNP |
| QUAC_G_SH-Q1717 | Garden | Trompenburg Tuinen & Arboretum | MSAT |
| QUAC_G_SH-Q1719 | Garden | Peckerwood | MSAT/SNP |
| QUAC_G_SH-Q1720 | Garden | Peckerwood | MSAT |
| QUAC_G_SH-Q1721 | Garden | Peckerwood | MSAT |
| QUAC_G_SH-Q1722 | Garden | Peckerwood | MSAT/SNP |
| QUAC_G_SH-Q1727 | Garden | Peckerwood | MSAT/SNP |
| QUAC_G_SH-Q1728 | Garden | Peckerwood | MSAT/SNP |
| QUAC_G_SH-Q1730 | Garden | Peckerwood | MSAT/SNP |
| QUAC_G_SH-Q1828 | Garden | Morris Arboretum | MSAT |
| QUAC_G_SH-Q1831 | Garden | Morris Arboretum | MSAT |
| QUAC_G_SH-Q1832 | Garden | Morris Arboretum | MSAT/SNP |
| QUAC_G_SH-Q1833 | Garden | Morris Arboretum | MSAT |
| QUAC_G_SH-Q1834 | Garden | Morris Arboretum | MSAT |
| QUAC_G_SH-Q1847 | Garden | Chicago Botanic Gardens | MSAT |
| QUAC_G_SH-Q1848 | Garden | Chicago Botanic Gardens | MSAT |
| QUAC_G_SH-Q2020 | Garden | Chicago Botanic Gardens | MSAT |
| QUAC_G_SH-Q2021 | Garden | Chicago Botanic Gardens | MSAT/SNP |
| QUAC_G_SH-Q2022 | Garden | Chicago Botanic Gardens | MSAT |
| QUAC_G_SH-Q2023 | Garden | Chicago Botanic Gardens | MSAT |
| QUAC_G_SH-Q2024 | Garden | Chicago Botanic Gardens | MSAT |
| QUAC_G_SH-Q2025 | Garden | Chicago Botanic Gardens | MSAT/SNP |
| QUAC_G_SH-Q2026 | Garden | Chicago Botanic Gardens | MSAT/SNP |
| QUAC_G_SH-Q2029 | Garden | Chicago Botanic Gardens | MSAT |
| QUAC_G_SH-Q2030 | Garden | Chicago Botanic Gardens | MSAT |
| QUAC_G_SH-Q2031 | Garden | Chicago Botanic Gardens | MSAT |
| QUAC_G_SH-Q2032 | Garden | Chicago Botanic Gardens | MSAT |
| QUAC_G_SH-Q2033 | Garden | Chicago Botanic Gardens | MSAT/SNP |
| QUAC_G_SH-Q2034 | Garden | Chicago Botanic Gardens | MSAT/SNP |
| QUAC_G_SH-Q2037 | Garden | Chicago Botanic Gardens | MSAT/SNP |
| QUAC_G_SH-Q2038 | Garden | Chicago Botanic Gardens | MSAT |
| QUAC_G_SH-Q2039 | Garden | Chicago Botanic Gardens | MSAT/SNP |
| QUAC_G_SH-Q2040 | Garden | Chicago Botanic Gardens | MSAT |
| QUAC_G_SH-Q2041 | Garden | Chicago Botanic Gardens | MSAT |
| QUAC_G_SH-Q2044 | Garden | Chicago Botanic Gardens | MSAT |
| QUAC_G_SH-Q2045 | Garden | Chicago Botanic Gardens | MSAT/SNP |
| QUAC_G_SH-Q2046 | Garden | Chicago Botanic Gardens | MSAT/SNP |
| QUAC_G_SH-Q2047 | Garden | Chicago Botanic Gardens | MSAT/SNP |
| QUAC_G_SH-Q2048 | Garden | Chicago Botanic Gardens | MSAT/SNP |
| QUAC_G_SH-Q2049 | Garden | Chicago Botanic Gardens | MSAT |
| QUAC_G_SH-Q2175 | Garden | The Morton Arboretum | MSAT |
| QUAC_G_SH-Q2187 | Garden | Chicago Botanic Gardens | MSAT |
| QUAC_G_SH-Q2188 | Garden | Peckerwood | MSAT/SNP |
| QUAC_G_SH-Q2189 | Garden | Peckerwood | MSAT |
| QUAC_G_SH-Q2190 | Garden | Chicago Botanic Gardens | MSAT/SNP |
| QUAC_G_SH-Q2191 | Garden | Peckerwood | MSAT/SNP |
| QUAC_G_SH-Q2192 | Garden | Peckerwood | MSAT |
| QUAC_G_SH-Q2193 | Garden | Chicago Botanic Gardens | MSAT |
| QUAC_G_SH-Q2194 | Garden | The Morton Arboretum | MSAT |
| QUAC_G_SH-Q2196 | Garden | Chicago Botanic Gardens | MSAT |
| QUAC_G_SH-Q2197 | Garden | Chicago Botanic Gardens | MSAT |
| QUAC_G_SH-Q2198 | Garden | Chicago Botanic Gardens | MSAT |
| QUAC_G_SH-Q2199 | Garden | Arboretum Pouyouleix | MSAT/SNP |
| QUAC_G_SH-Q2200 | Garden | Bartlett Tree Research Laboratories | MSAT/SNP |
| QUAC_G_SH-Q2201 | Garden | Bartlett Tree Research Laboratories | MSAT/SNP |
| QUAC_G_SH-Q2202 | Garden | Forstbotanischer Garten Tharandt | MSAT/SNP |
| QUAC_G_SH-Q2203 | Garden | Forstbotanischer Garten Tharandt | MSAT/SNP |
| QUAC_G_SH-Q2204 | Garden | Forstbotanischer Garten Tharandt | MSAT/SNP |
| **QUAC_G_DUP_SH-Q2204** | **Garden** | **Forstbotanischer Garten Tharandt** | **SNP** |
| QUAC_G_SH-Q2205 | Garden | Forstbotanischer Garten Tharandt | SNP |
| QUAC_G_SH-Q2206 | Garden | Forstbotanischer Garten Tharandt | MSAT/SNP |
| QUAC_G_SH-Q2207 | Garden | Meise Botanic Garden | MSAT/SNP |
| QUAC_G_SH-Q2208 | Garden | Missouri Botanical Garden | SNP |
| QUAC_G_SH-Q2209 | Garden | Moore Farms Botanical Garden | MSAT/SNP |
| QUAC_G_SH-Q2210 | Garden | Morris Arboretum | MSAT/SNP |
| QUAC_G_SH-Q2211 | Garden | Morris Arboretum | MSAT/SNP |
| QUAC_G_SH-Q2212 | Garden | Morris Arboretum | MSAT/SNP |
| QUAC_G_SH-Q2213 | Garden | Morris Arboretum | MSAT/SNP |
| QUAC_G_SH-Q2214 | Garden | Morris Arboretum | MSAT/SNP |
| QUAC_G_SH-Q2215 | Garden | Peckerwood | MSAT/SNP |
| QUAC_G_SH-Q2216 | Garden | The Morton Arboretum | MSAT/SNP |
| QUAC_G_SH-Q2217 | Garden | The Morton Arboretum | MSAT/SNP |
| QUAC_G_SH-Q2218 | Garden | The Morton Arboretum | MSAT |
| QUAC_G_SH-Q2219 | Garden | The Morton Arboretum | MSAT/SNP |
| QUAC_G_SH-Q2220 | Garden | The Morton Arboretum | MSAT |
| QUAC_G_SH-Q2221 | Garden | The Morton Arboretum | MSAT |
| QUAC_G_SH-Q2222 | Garden | The Morton Arboretum | MSAT/SNP |
| QUAC_G_SH-Q2223 | Garden | United States National Arboretum | MSAT/SNP |
| **QUAC_G_DUP_SH-Q2223** | **Garden** | **United States National Arboretum** | **SNP** |
| QUAC_G_SH-Q2224 | Garden | United States National Arboretum | SNP |
| QUAC_G_SH-Q2225 | Garden | University of Washington Botanic Gardens | MSAT/SNP |
| QUAC_G_SH-Q2226 | Garden | Zoo and BG Plzen | SNP |
| QUAC_G_SH-Q2227 | Garden | Zoo and BG Plzeň | MSAT/SNP |
| QUAC_W_SH-Q1849 | Wild | Porter Mountain | MSAT |
| QUAC_W_SH-Q1850 | Wild | Porter Mountain | MSAT/SNP |
| QUAC_W_SH-Q1851 | Wild | Porter Mountain | MSAT |
| QUAC_W_SH-Q1852 | Wild | Porter Mountain | MSAT |
| QUAC_W_SH-Q1853 | Wild | Porter Mountain | MSAT |
| QUAC_W_SH-Q1854 | Wild | Porter Mountain | MSAT/SNP |
| QUAC_W_SH-Q1855 | Wild | Porter Mountain | MSAT |
| QUAC_W_SH-Q1856 | Wild | Porter Mountain | MSAT/SNP |
| QUAC_W_SH-Q1857 | Wild | Porter Mountain | MSAT |
| QUAC_W_SH-Q1858 | Wild | Porter Mountain | MSAT |
| QUAC_W_SH-Q1859 | Wild | Porter Mountain | MSAT |
| QUAC_W_SH-Q1860 | Wild | Porter Mountain | MSAT |
| QUAC_W_SH-Q1861 | Wild | Porter Mountain | MSAT |
| QUAC_W_SH-Q1862 | Wild | Porter Mountain | MSAT/SNP |
| QUAC_W_SH-Q1863 | Wild | Porter Mountain | MSAT |
| QUAC_W_SH-Q1864 | Wild | Porter Mountain | MSAT |
| QUAC_W_SH-Q1865 | Wild | Porter Mountain | MSAT/SNP |
| QUAC_W_SH-Q1866 | Wild | Porter Mountain | MSAT/SNP |
| QUAC_W_SH-Q1867 | Wild | Porter Mountain | MSAT |
| QUAC_W_SH-Q1868 | Wild | Porter Mountain | MSAT/SNP |
| QUAC_W_SH-Q1869 | Wild | Porter Mountain | MSAT/SNP |
| QUAC_W_SH-Q1870 | Wild | Porter Mountain | MSAT/SNP |
| QUAC_W_SH-Q1871 | Wild | Porter Mountain | MSAT/SNP |
| QUAC_W_SH-Q1872 | Wild | Porter Mountain | MSAT/SNP |
| QUAC_W_SH-Q1873 | Wild | Porter Mountain | MSAT/SNP |
| QUAC_W_SH-Q1874 | Wild | Porter Mountain | MSAT/SNP |
| QUAC_W_SH-Q1875 | Wild | Porter Mountain | MSAT/SNP |
| QUAC_W_SH-Q1876 | Wild | Porter Mountain | MSAT/SNP |
| QUAC_W_SH-Q1877 | Wild | Porter Mountain | MSAT/SNP |
| QUAC_W_SH-Q1878 | Wild | Porter Mountain | MSAT/SNP |
| QUAC_W_SH-Q1879 | Wild | Porter Mountain | MSAT/SNP |
| QUAC_W_SH-Q1880 | Wild | Porter Mountain | MSAT/SNP |
| QUAC_W_SH-Q1881 | Wild | Porter Mountain | MSAT/SNP |
| QUAC_W_SH-Q1882 | Wild | Porter Mountain | MSAT/SNP |
| QUAC_W_SH-Q1883 | Wild | Porter Mountain | MSAT |
| QUAC_W_SH-Q1884 | Wild | Porter Mountain | MSAT |
| QUAC_W_SH-Q1885 | Wild | Porter Mountain | MSAT/SNP |
| QUAC_W_SH-Q1886 | Wild | Porter Mountain | MSAT/SNP |
| QUAC_W_SH-Q2000 | Wild | Porter Mountain | MSAT |
| QUAC_W_SH-Q2001 | Wild | Porter Mountain | MSAT/SNP |
| QUAC_W_SH-Q2002 | Wild | Porter Mountain | MSAT/SNP |
| QUAC_W_SH-Q2003 | Wild | Magazine Mountain | MSAT/SNP |
| QUAC_W_SH-Q2004 | Wild | Magazine Mountain | MSAT |
| QUAC_W_SH-Q2005 | Wild | Magazine Mountain | MSAT |
| QUAC_W_SH-Q2006 | Wild | Magazine Mountain | MSAT/SNP |
| QUAC_W_SH-Q2007 | Wild | Magazine Mountain | MSAT/SNP |
| QUAC_W_SH-Q2008 | Wild | Magazine Mountain | MSAT/SNP |
| QUAC_W_SH-Q2009 | Wild | Magazine Mountain | MSAT |
| QUAC_W_SH-Q2010 | Wild | Magazine Mountain | MSAT |
| QUAC_W_SH-Q2050 | Wild | Magazine Mountain | MSAT/SNP |
| QUAC_W_SH-Q2051 | Wild | Magazine Mountain | MSAT/SNP |
| QUAC_W_SH-Q2052 | Wild | Magazine Mountain | MSAT |
| QUAC_W_SH-Q2053 | Wild | Magazine Mountain | MSAT |
| QUAC_W_SH-Q2054 | Wild | Magazine Mountain | MSAT |
| QUAC_W_SH-Q2055 | Wild | Magazine Mountain | MSAT/SNP |
| QUAC_W_SH-Q2056 | Wild | Magazine Mountain | MSAT/SNP |
| QUAC_W_SH-Q2057 | Wild | Magazine Mountain | MSAT/SNP |
| QUAC_W_SH-Q2058 | Wild | Magazine Mountain | MSAT/SNP |
| QUAC_W_SH-Q2059 | Wild | Magazine Mountain | MSAT/SNP |
| QUAC_W_SH-Q2060 | Wild | Magazine Mountain | MSAT/SNP |
| QUAC_W_SH-Q2061 | Wild | Magazine Mountain | MSAT/SNP |
| QUAC_W_SH-Q2062 | Wild | Magazine Mountain | MSAT/SNP |
| QUAC_W_SH-Q2063 | Wild | Magazine Mountain | MSAT/SNP |
| QUAC_W_SH-Q2064 | Wild | Magazine Mountain | MSAT/SNP |
| QUAC_W_SH-Q2065 | Wild | Magazine Mountain | MSAT |
| QUAC_W_SH-Q2066 | Wild | Magazine Mountain | MSAT/SNP |
| QUAC_W_SH-Q2067 | Wild | Magazine Mountain | MSAT |
| QUAC_W_SH-Q2068 | Wild | Magazine Mountain | MSAT/SNP |
| QUAC_W_SH-Q2069 | Wild | Magazine Mountain | MSAT |
| QUAC_W_SH-Q2070 | Wild | Magazine Mountain | MSAT/SNP |
| QUAC_W_SH-Q2071 | Wild | Magazine Mountain | MSAT/SNP |
| QUAC_W_SH-Q2072 | Wild | Magazine Mountain | MSAT |
| QUAC_W_SH-Q2073 | Wild | Magazine Mountain | MSAT/SNP |
| QUAC_W_SH-Q2074 | Wild | Magazine Mountain | MSAT/SNP |
| QUAC_W_SH-Q2075 | Wild | Magazine Mountain | MSAT |
| QUAC_W_SH-Q2076 | Wild | Magazine Mountain | MSAT/SNP |
| QUAC_W_SH-Q2077 | Wild | Magazine Mountain | MSAT |
| QUAC_W_SH-Q2078 | Wild | Pryor Mountain | MSAT/SNP |
| QUAC_W_SH-Q2079 | Wild | Pryor Mountain | MSAT |
| QUAC_W_SH-Q2080 | Wild | Pryor Mountain | MSAT/SNP |
| QUAC_W_SH-Q2081 | Wild | Pryor Mountain | MSAT |
| QUAC_W_SH-Q2082 | Wild | Pryor Mountain | MSAT/SNP |
| QUAC_W_SH-Q2083 | Wild | Pryor Mountain | MSAT |
| QUAC_W_SH-Q2084 | Wild | Pryor Mountain | MSAT/SNP |
| QUAC_W_SH-Q2085 | Wild | Pryor Mountain | MSAT |
| QUAC_W_SH-Q2086 | Wild | Pryor Mountain | MSAT/SNP |
| QUAC_W_SH-Q2087 | Wild | Pryor Mountain | MSAT |
| QUAC_W_SH-Q2088 | Wild | Pryor Mountain | MSAT/SNP |
| QUAC_W_SH-Q2089 | Wild | Pryor Mountain | MSAT |
| QUAC_W_SH-Q2090 | Wild | Pryor Mountain | MSAT/SNP |
| QUAC_W_SH-Q2091 | Wild | Pryor Mountain | MSAT/SNP |
| QUAC_W_SH-Q2092 | Wild | Pryor Mountain | MSAT/SNP |
| QUAC_W_SH-Q2093 | Wild | Pryor Mountain | MSAT/SNP |
| QUAC_W_SH-Q2094 | Wild | Pryor Mountain | MSAT/SNP |
| QUAC_W_SH-Q2095 | Wild | Pryor Mountain | MSAT |
| QUAC_W_SH-Q2096 | Wild | Pryor Mountain | MSAT/SNP |
| QUAC_W_SH-Q2097 | Wild | Pryor Mountain | MSAT |
| QUAC_W_SH-Q2098 | Wild | Pryor Mountain | MSAT |
| QUAC_W_SH-Q2099 | Wild | Pryor Mountain | MSAT |
| QUAC_W_SH-Q2100 | Wild | Pryor Mountain | MSAT/SNP |
| QUAC_W_SH-Q2101 | Wild | Pryor Mountain | MSAT/SNP |
| QUAC_W_SH-Q2104 | Wild | Pryor Mountain | MSAT |
| QUAC_W_SH-Q2105 | Wild | Pryor Mountain | MSAT/SNP |
| QUAC_W_SH-Q2106 | Wild | Pryor Mountain | MSAT |
| QUAC_W_SH-Q2107 | Wild | Pryor Mountain | MSAT |
| QUAC_W_SH-Q2108 | Wild | Pryor Mountain | MSAT/SNP |
| QUAC_W_SH-Q2109 | Wild | Pryor Mountain | MSAT |
| QUAC_W_SH-Q2110 | Wild | Pryor Mountain | MSAT |
| QUAC_W_SH-Q2111 | Wild | Pryor Mountain | MSAT/SNP |
| QUAC_W_SH-Q2112 | Wild | Pryor Mountain | MSAT |
| QUAC_W_SH-Q2113 | Wild | Sugar Loaf Mountains - Midland Peak | MSAT/SNP |
| QUAC_W_SH-Q2114 | Wild | Sugar Loaf Mountains - Midland Peak | MSAT/SNP |
| QUAC_W_SH-Q2115 | Wild | Sugar Loaf Mountains - Midland Peak | MSAT/SNP |
| QUAC_W_SH-Q2116 | Wild | Sugar Loaf Mountains - Midland Peak | MSAT |
| QUAC_W_SH-Q2117 | Wild | Sugar Loaf Mountains - Midland Peak | MSAT/SNP |
| QUAC_W_SH-Q2118 | Wild | Sugar Loaf Mountains - Midland Peak | MSAT/SNP |
| QUAC_W_SH-Q2119 | Wild | Sugar Loaf Mountains - Midland Peak | MSAT/SNP |
| QUAC_W_SH-Q2120 | Wild | Sugar Loaf Mountains - Midland Peak | MSAT/SNP |
| QUAC_W_SH-Q2121 | Wild | Sugar Loaf Mountains - Midland Peak | MSAT/SNP |
| **QUAC_W_DUP_SH-Q2121** | **Wild** | **Sugar Loaf Mountains - Midland Peak** | **SNP** |
| QUAC_W_SH-Q2122 | Wild | Sugar Loaf Mountains - Midland Peak | MSAT/SNP |
| QUAC_W_SH-Q2123 | Wild | Sugar Loaf Mountains - Midland Peak | MSAT/SNP |
| QUAC_W_SH-Q2124 | Wild | Sugar Loaf Mountains - Midland Peak | MSAT/SNP |
| QUAC_W_SH-Q2125 | Wild | Sugar Loaf Mountains - Midland Peak | MSAT/SNP |
| QUAC_W_SH-Q2126 | Wild | Sugar Loaf Mountains - Midland Peak | MSAT/SNP |
| QUAC_W_SH-Q2127 | Wild | Sugar Loaf Mountains - Midland Peak | MSAT/SNP |
| QUAC_W_SH-Q2128 | Wild | Sugar Loaf Mountains - Midland Peak | MSAT |
| QUAC_W_SH-Q2129 | Wild | Sugar Loaf Mountains - Midland Peak | MSAT |
| QUAC_W_SH-Q2130 | Wild | Sugar Loaf Mountains - Midland Peak | MSAT |
| QUAC_W_SH-Q2131 | Wild | Sugar Loaf Mountains - Midland Peak | MSAT |
| QUAC_W_SH-Q2132 | Wild | Sugar Loaf Mountains - Midland Peak | MSAT |
| QUAC_W_SH-Q2133 | Wild | Sugar Loaf Mountains - Midland Peak | MSAT |
| QUAC_W_SH-Q2134 | Wild | Sugar Loaf Mountains - Midland Peak | MSAT |
| QUAC_W_SH-Q2135 | Wild | Sugar Loaf Mountains - Midland Peak | MSAT/SNP |
| QUAC_W_SH-Q2136 | Wild | Sugar Loaf Mountains - Midland Peak | MSAT/SNP |
| QUAC_W_SH-Q2137 | Wild | Sugar Loaf Mountains - Midland Peak | MSAT |
| QUAC_W_SH-Q2138 | Wild | Sugar Loaf Mountains - Midland Peak | MSAT |
| QUAC_W_SH-Q2139 | Wild | Sugar Loaf Mountains - Midland Peak | MSAT |
| QUAC_W_SH-Q2140 | Wild | Sugar Loaf Mountains - Midland Peak | MSAT |
| QUAC_W_SH-Q2141 | Wild | Sugar Loaf Mountains - Midland Peak | MSAT |
| QUAC_W_SH-Q2142 | Wild | Sugar Loaf Mountains - Midland Peak | MSAT/SNP |
| QUAC_W_SH-Q2143 | Wild | Sugar Loaf Mountains - Midland Peak | MSAT |
| QUAC_W_SH-Q2144 | Wild | Sugar Loaf Mountains - Midland Peak | MSAT |
| QUAC_W_SH-Q2145 | Wild | Sugar Loaf Mountains - Midland Peak | MSAT |
| QUAC_W_SH-Q2146 | Wild | Sugar Loaf Mountains - Midland Peak | MSAT |
| QUAC_W_SH-Q2147 | Wild | Sugar Loaf Mountains - Midland Peak | MSAT/SNP |
| QUAC_W_SH-Q2148 | Wild | Sugar Loaf Mountains - Midland Peak | MSAT |
| QUAC_W_SH-Q2149 | Wild | Sugar Loaf Mountains - Midland Peak | MSAT |
| QUAC_W_SH-Q2150 | Wild | Sugar Loaf Mountains - Midland Peak | MSAT |
| QUAC_W_SH-Q2151 | Wild | Sugar Loaf Mountains - Midland Peak | MSAT/SNP |
| QUAC_W_SH-Q2152 | Wild | Sugar Loaf Mountains - Midland Peak | MSAT/SNP |
| QUAC_W_SH-Q2153 | Wild | Sugar Loaf Mountains - Midland Peak | MSAT/SNP |
| QUAC_W_SH-Q2154 | Wild | Sugar Loaf Mountains - Midland Peak | MSAT |
| QUAC_W_SH-Q2155 | Wild | Sugar Loaf Mountains - Midland Peak | MSAT/SNP |
| QUAC_W_SH-Q2156 | Wild | Sugar Loaf Mountains - Midland Peak | MSAT |
| QUAC_W_SH-Q2157 | Wild | Sugar Loaf Mountains - Midland Peak | MSAT/SNP |
| QUAC_W_SH-Q2158 | Wild | Sugar Loaf Mountains - Midland Peak | MSAT/SNP |
| QUAC_W_SH-Q2159 | Wild | Sugar Loaf Mountains - Midland Peak | MSAT |
| QUAC_W_SH-Q2160 | Wild | Sugar Loaf Mountains - Midland Peak | MSAT |
| QUAC_W_SH-Q2161 | Wild | Sugar Loaf Mountains - Midland Peak | MSAT/SNP |
| QUAC_W_SH-Q2162 | Wild | Sugar Loaf Mountains - Midland Peak | MSAT |
| QUAC_W_SH-Q2163 | Wild | Sugar Loaf Mountains - Midland Peak | MSAT/SNP |
| QUAC_W_SH-Q2164 | Wild | Sugar Loaf Mountains - Midland Peak | MSAT |
| QUAC_W_SH-Q2165 | Wild | Sugar Loaf Mountains - Midland Peak | MSAT |
| QUAC_W_SH-Q2166 | Wild | Sugar Loaf Mountains - Midland Peak | MSAT |
| **QUAC_W_SH-Q2168** | **Wild** | **Kessler Mountain Regional Park - Shale Barren ridge** | **SNP** |
| **QUAC_W_SH-Q2170** | **Wild** | **Kessler Mountain Regional Park - Shale Barren ridge** | **SNP** |
| **QUAC_W_SH-Q2171** | **Wild** | **Kessler Mountain Regional Park - Shale Barren ridge** | **SNP** |
| **QUAC_W_SH-Q2172** | **Wild** | **Kessler Mountain Regional Park - Shale Barren ridge** | **SNP** |
| **QUAC_W_SH-Q2173** | **Wild** | **Kessler Mountain Regional Park - Shale Barren ridge** | **SNP** |
| **QUAC_W_SH-Q2174** | **Wild** | **Kessler Mountain Regional Park - Shale Barren ridge** | **SNP** |

##### Table S1: Microsatellite and NextRAD samples of *Q. acerifolia*. Samples in bold were excluded from final analyses, either because they are duplicates or because they are individuals sourced from the Kessler population, which was removed from analyses due to strong signals of hybridization with *Q. shumardii* within the population.

| *Sample Name* | *Garden/Wild* | *Garden or Wild Population Name* | *Marker Study* |
| --- | --- | --- | --- |
| QUBO_G_IMLS078 | Garden | Lady Bird Johnson | SNP |
| QUBO_G_IMLS079 | Garden | Lady Bird Johnson | SNP |
| QUBO_G_IMLS080 | Garden | Lady Bird Johnson | SNP |
| QUBO_G_IMLS081 | Garden | Lady Bird Johnson | SNP |
| QUBO_G_IMLS082 | Garden | Lady Bird Johnson | SNP |
| QUBO_G_IMLS083 | Garden | Lady Bird Johnson | SNP |
| QUBO_G_IMLS084 | Garden | Lady Bird Johnson | SNP |
| QUBO_G_IMLS085 | Garden | Lady Bird Johnson | SNP |
| QUBO_G_IMLS086 | Garden | Lady Bird Johnson | SNP |
| QUBO_G_IMLS087 | Garden | Lady Bird Johnson | SNP |
| QUBO_G_IMLS313 | Garden | Atlanta Botanical Garden | MSAT/SNP |
| QUBO_G_IMLS314 | Garden | Bartlett Tree Research Laboratories Arboretum | MSAT/SNP |
| QUBO_G_IMLS315 | Garden | Bartlett Tree Research Laboratories Arboretum | MSAT/SNP |
| QUBO_G_IMLS316 | Garden | National Arboretum/GRIN | MSAT/SNP |
| QUBO_G_IMLS317 | Garden | Mt. Cuba Center | MSAT/SNP |
| QUBO_G_IMLS318 | Garden | Moore Farms Botanic Garden | MSAT/SNP |
| QUBO_G_IMLS319 | Garden | Moore Farms Botanic Garden | MSAT/SNP |
| **QUBO_G_IMLS320** | **Garden** | **Moore Farms Botanic Garden** | **MSAT/SNP** |
| QUBO_G_IMLS321 | Garden | Moore Farms Botanic Garden | MSAT/SNP |
| QUBO_G_IMLS322 | Garden | Moore Farms Botanic Garden | MSAT/SNP |
| QUBO_G_IMLS323 | Garden | Moore Farms Botanic Garden | MSAT/SNP |
| QUBO_G_IMLS324 | Garden | The Keith Arboretum | MSAT/SNP |
| QUBO_G_IMLS325 | Garden | The Keith Arboretum | MSAT/SNP |
| QUBO_G_IMLS326 | Garden | Starhill Forest Arboretum | MSAT/SNP |
| QUBO_G_IMLS327 | Garden | Starhill Forest Arboretum | MSAT/SNP |
| QUBO_G_IMLS328 | Garden | The Morris Arboretum | MSAT/SNP |
| QUBO_G_IMLS329 | Garden | The Morris Arboretum | MSAT/SNP |
| QUBO_G_IMLS330 | Garden | The Donald E. Davis Arboretum | MSAT/SNP |
| QUBO_G_IMLS331 | Garden | The Donald E. Davis Arboretum | MSAT/SNP |
| QUBO_G_IMLS332 | Garden | University of Washington Botanic Garden | MSAT/SNP |
| QUBO_G_IMLS333 | Garden | University of Washington Botanic Garden | MSAT/SNP |
| QUBO_G_IMLS334 | Garden | University of Washington Botanic Garden | MSAT/SNP |
| QUBO_G_IMLS335 | Garden | University of Washington Botanic Garden | MSAT/SNP |
| QUBO_G_IMLS336 | Garden | University of Washington Botanic Garden | MSAT/SNP |
| QUBO_G_IMLS337 | Garden | The Donald E. Davis Arboretum | MSAT/SNP |
| QUBO_G_IMLS338 | Garden | The Donald E. Davis Arboretum | MSAT/SNP |
| QUBO_G_IMLS339 | Garden | The Donald E. Davis Arboretum | MSAT/SNP |
| QUBO_G_IMLS340 | Garden | The Donald E. Davis Arboretum | MSAT/SNP |
| QUBO_G_IMLS341 | Garden | The Donald E. Davis Arboretum | MSAT/SNP |
| QUBO_G_IMLS342 | Garden | The Donald E. Davis Arboretum | MSAT/SNP |
| QUBO_G_IMLS343 | Garden | The Donald E. Davis Arboretum | MSAT/SNP |
| QUBO_G_IMLS344 | Garden | The Donald E. Davis Arboretum | MSAT/SNP |
| QUBO_G_IMLS345 | Garden | Gainesway Farm | MSAT/SNP |
| QUBO_G_IMLS346 | Garden | The Morton Arboretum | MSAT/SNP |
| QUBO_G_IMLS347 | Garden | The Morton Arboretum | MSAT/SNP |
| QUBO_G_IMLS348 | Garden | The Morton Arboretum | MSAT/SNP |
| QUBO_G_IMLS349 | Garden | The Morton Arboretum | MSAT/SNP |
| QUBO_G_IMLS350 | Garden | The Morton Arboretum | MSAT/SNP |
| QUBO_G_IMLS351 | Garden | The Morton Arboretum | MSAT/SNP |
| QUBO_G_IMLS352 | Garden | Missouri Botanical Garden | MSAT/SNP |
| QUBO_G_IMLS353 | Garden | Missouri Botanical Garden | MSAT/SNP |
| QUBO_G_IMLS354 | Garden | The Morton Arboretum | MSAT/SNP |
| QUBO_G_IMLS355 | Garden | Chicago Botanic Garden | MSAT/SNP |
| QUBO_G_IMLS356 | Garden | Chicago Botanic Garden | MSAT/SNP |
| QUBO_G_IMLS357 | Garden | Chicago Botanic Garden | MSAT/SNP |
| QUBO_G_IMLS358 | Garden | Chicago Botanic Garden | MSAT/SNP |
| QUBO_G_IMLS359 | Garden | Chicago Botanic Garden | MSAT/SNP |
| QUBO_G_IMLS360 | Garden | Chicago Botanic Garden | MSAT |
| QUBO_G_IMLS361 | Garden | Chicago Botanic Garden | MSAT/SNP |
| QUBO_G_IMLS362 | Garden | Chicago Botanic Garden | MSAT/SNP |
| QUBO_G_IMLS363 | Garden | Chicago Botanic Garden | MSAT/SNP |
| QUBO_G_IMLS364 | Garden | Chicago Botanic Garden | MSAT/SNP |
| QUBO_G_IMLS365 | Garden | Chicago Botanic Garden | MSAT/SNP |
| QUBO_G_IMLS366 | Garden | Chicago Botanic Garden | MSAT/SNP |
| QUBO_G_IMLS367 | Garden | Chicago Botanic Garden | MSAT/SNP |
| QUBO_G_IMLS368 | Garden | Chicago Botanic Garden | MSAT/SNP |
| QUBO_G_IMLS369 | Garden | Chicago Botanic Garden | MSAT/SNP |
| QUBO_G_IMLS370 | Garden | The Morton Arboretum | MSAT/SNP |
| QUBO_G_IMLS371 | Garden | The Morton Arboretum | MSAT/SNP |
| QUBO_G_IMLS372 | Garden | The Morton Arboretum | MSAT/SNP |
| QUBO_G_IMLS373 | Garden | The Morton Arboretum | MSAT/SNP |
| QUBO_G_IMLS374 | Garden | The Morton Arboretum | MSAT/SNP |
| QUBO_G_IMLS375 | Garden | The Morton Arboretum | MSAT/SNP |
| QUBO_G_IMLS376 | Garden | The Morton Arboretum | MSAT/SNP |
| QUBO_G_IMLS377 | Garden | The Morton Arboretum | MSAT/SNP |
| QUBO_G_IMLS378 | Garden | The Morton Arboretum | MSAT/SNP |
| QUBO_G_IMLS379 | Garden | The Morton Arboretum | MSAT/SNP |
| QUBO_G_IMLS380 | Garden | The Morton Arboretum | MSAT/SNP |
| QUBO_G_IMLS381 | Garden | The Morton Arboretum | MSAT/SNP |
| QUBO_G_IMLS382 | Garden | The Morton Arboretum | MSAT/SNP |
| QUBO_G_IMLS383 | Garden | The Morton Arboretum | MSAT/SNP |
| QUBO_G_IMLS384 | Garden | The Morton Arboretum | MSAT/SNP |
| QUBO_G_IMLS385 | Garden | The Morton Arboretum | MSAT/SNP |
| QUBO_G_IMLS386 | Garden | The Morton Arboretum | MSAT/SNP |
| QUBO_G_IMLS387 | Garden | The Morton Arboretum | MSAT/SNP |
| QUBO_G_IMLS388 | Garden | The Morton Arboretum | MSAT/SNP |
| QUBO_G_IMLS389 | Garden | The Morton Arboretum | MSAT/SNP |
| **QUBO_W_DUP_IMLS139** | **Wild** | **Oak Mountain State Park** | **SNP** |
| QUBO_W_IMLS001 | Wild | Oakbrook | MSAT/SNP |
| QUBO_W_IMLS002 | Wild | Oakbrook | MSAT/SNP |
| QUBO_W_IMLS003 | Wild | Worldsong | MSAT |
| QUBO_W_IMLS004 | Wild | Worldsong | MSAT/SNP |
| QUBO_W_IMLS005 | Wild | Wattsville | MSAT/SNP |
| QUBO_W_IMLS006 | Wild | Wattsville | MSAT |
| QUBO_W_IMLS007 | Wild | Moss Rock Preserve | MSAT |
| QUBO_W_IMLS008 | Wild | Moss Rock Preserve | MSAT |
| QUBO_W_IMLS009 | Wild | Irondale | MSAT/SNP |
| QUBO_W_IMLS010 | Wild | Irondale | MSAT/SNP |
| QUBO_W_IMLS011 | Wild | Oak Mountain State Park | MSAT/SNP |
| QUBO_W_IMLS012 | Wild | Oak Mountain State Park | MSAT |
| QUBO_W_IMLS013 | Wild | Hinds Road | MSAT |
| QUBO_W_IMLS014 | Wild | Hinds Road | MSAT |
| QUBO_W_IMLS015 | Wild | Oak Mountain State Park | MSAT/SNP |
| QUBO_W_IMLS016 | Wild | Oak Mountain State Park | MSAT/SNP |
| QUBO_W_IMLS017 | Wild | Oak Mountain State Park | MSAT/SNP |
| QUBO_W_IMLS018 | Wild | Oak Mountain State Park | MSAT/SNP |
| QUBO_W_IMLS019 | Wild | Peavine Falls | MSAT |
| QUBO_W_IMLS020 | Wild | Peavine Falls | MSAT/SNP |
| QUBO_W_IMLS021 | Wild | Worldsong | MSAT/SNP |
| QUBO_W_IMLS022 | Wild | Worldsong | MSAT |
| QUBO_W_IMLS023 | Wild | Worldsong | MSAT |
| QUBO_W_IMLS024 | Wild | Worldsong | MSAT/SNP |
| QUBO_W_IMLS025 | Wild | Worldsong | MSAT |
| QUBO_W_IMLS026 | Wild | Worldsong | MSAT/SNP |
| QUBO_W_IMLS027 | Wild | Worldsong | MSAT/SNP |
| QUBO_W_IMLS028 | Wild | Worldsong | MSAT |
| QUBO_W_IMLS029 | Wild | Worldsong | MSAT/SNP |
| QUBO_W_IMLS030 | Wild | Worldsong | MSAT |
| QUBO_W_IMLS031 | Wild | Worldsong | MSAT/SNP |
| QUBO_W_IMLS032 | Wild | Worldsong | MSAT |
| QUBO_W_IMLS033 | Wild | Wattsville | MSAT |
| QUBO_W_IMLS034 | Wild | Wattsville | MSAT/SNP |
| QUBO_W_IMLS035 | Wild | Wattsville | MSAT |
| QUBO_W_IMLS036 | Wild | Wattsville | MSAT |
| QUBO_W_IMLS037 | Wild | Wattsville | MSAT/SNP |
| QUBO_W_IMLS038 | Wild | Wattsville | MSAT/SNP |
| QUBO_W_IMLS039 | Wild | Wattsville | MSAT |
| QUBO_W_IMLS040 | Wild | Wattsville | SNP |
| QUBO_W_IMLS041 | Wild | Wattsville | MSAT |
| QUBO_W_IMLS042 | Wild | Wattsville | MSAT |
| QUBO_W_IMLS043 | Wild | Wattsville | MSAT/SNP |
| QUBO_W_IMLS044 | Wild | Wattsville | MSAT |
| QUBO_W_IMLS045 | Wild | Wattsville | MSAT/SNP |
| QUBO_W_IMLS046 | Wild | Wattsville | MSAT/SNP |
| QUBO_W_IMLS047 | Wild | Wattsville | MSAT/SNP |
| QUBO_W_IMLS048 | Wild | Wattsville | MSAT/SNP |
| QUBO_W_IMLS049 | Wild | Moss Rock Preserve | MSAT |
| QUBO_W_IMLS050 | Wild | Moss Rock Preserve | MSAT |
| QUBO_W_IMLS051 | Wild | Moss Rock Preserve | MSAT/SNP |
| QUBO_W_IMLS052 | Wild | Moss Rock Preserve | MSAT/SNP |
| QUBO_W_IMLS053 | Wild | Moss Rock Preserve | MSAT |
| QUBO_W_IMLS054 | Wild | Moss Rock Preserve | MSAT |
| QUBO_W_IMLS055 | Wild | Moss Rock Preserve | MSAT |
| QUBO_W_IMLS056 | Wild | Moss Rock Preserve | MSAT |
| QUBO_W_IMLS057 | Wild | Moss Rock Preserve | MSAT |
| QUBO_W_IMLS058 | Wild | Moss Rock Preserve | MSAT/SNP |
| QUBO_W_IMLS059 | Wild | Moss Rock Preserve | MSAT |
| QUBO_W_IMLS060 | Wild | Moss Rock Preserve | MSAT |
| QUBO_W_IMLS061 | Wild | Moss Rock Preserve | MSAT |
| QUBO_W_IMLS062 | Wild | Moss Rock Preserve | MSAT/SNP |
| QUBO_W_IMLS063 | Wild | Moss Rock Preserve | MSAT/SNP |
| QUBO_W_IMLS064 | Wild | Moss Rock Preserve | MSAT |
| QUBO_W_IMLS065 | Wild | Moss Rock Preserve | MSAT/SNP |
| QUBO_W_IMLS066 | Wild | Moss Rock Preserve | MSAT |
| QUBO_W_IMLS067 | Wild | Moss Rock Preserve | MSAT/SNP |
| QUBO_W_IMLS068 | Wild | Moss Rock Preserve | MSAT/SNP |
| QUBO_W_IMLS069 | Wild | Irondale | MSAT |
| QUBO_W_IMLS070 | Wild | Irondale | MSAT/SNP |
| QUBO_W_IMLS131 | Wild | Irondale | MSAT |
| QUBO_W_IMLS132 | Wild | Irondale | MSAT/SNP |
| QUBO_W_IMLS133 | Wild | Irondale | MSAT/SNP |
| QUBO_W_IMLS134 | Wild | Irondale | MSAT |
| QUBO_W_IMLS135 | Wild | Irondale | MSAT/SNP |
| QUBO_W_IMLS136 | Wild | Irondale | MSAT |
| QUBO_W_IMLS137 | Wild | Irondale | MSAT |
| QUBO_W_IMLS138 | Wild | Irondale | MSAT/SNP |
| QUBO_W_IMLS139 | Wild | Oak Mountain State Park | MSAT/SNP |
| QUBO_W_IMLS140 | Wild | Oak Mountain State Park | MSAT |
| QUBO_W_IMLS141 | Wild | Oak Mountain State Park | MSAT/SNP |
| QUBO_W_IMLS142 | Wild | Oak Mountain State Park | MSAT |
| QUBO_W_IMLS143 | Wild | Oak Mountain State Park | MSAT |
| QUBO_W_IMLS144 | Wild | Oak Mountain State Park | MSAT/SNP |
| QUBO_W_IMLS145 | Wild | Oak Mountain State Park | MSAT |
| QUBO_W_IMLS146 | Wild | Oak Mountain State Park | MSAT |
| QUBO_W_IMLS147 | Wild | Oak Mountain State Park | MSAT |
| QUBO_W_IMLS148 | Wild | Oak Mountain State Park | MSAT |
| QUBO_W_IMLS149 | Wild | Oak Mountain State Park | MSAT |
| QUBO_W_IMLS150 | Wild | Oak Mountain State Park | MSAT/SNP |
| QUBO_W_IMLS151 | Wild | Oak Mountain State Park | MSAT/SNP |
| QUBO_W_IMLS152 | Wild | Oak Mountain State Park | MSAT |
| QUBO_W_IMLS153 | Wild | Oak Mountain State Park | MSAT |
| QUBO_W_IMLS154 | Wild | Oak Mountain State Park | MSAT/SNP |
| QUBO_W_IMLS155 | Wild | Oak Mountain State Park | MSAT |
| QUBO_W_IMLS156 | Wild | Oak Mountain State Park | MSAT |
| QUBO_W_IMLS157 | Wild | Oak Mountain State Park | MSAT |
| QUBO_W_IMLS158 | Wild | Oak Mountain State Park | MSAT |
| QUBO_W_IMLS159 | Wild | Oak Mountain State Park | MSAT |
| QUBO_W_IMLS160 | Wild | Oak Mountain State Park | MSAT |
| QUBO_W_IMLS161 | Wild | Oak Mountain State Park | MSAT |
| QUBO_W_IMLS162 | Wild | Oak Mountain State Park | MSAT |
| QUBO_W_IMLS163 | Wild | Oak Mountain State Park | MSAT |
| QUBO_W_IMLS164 | Wild | Oak Mountain State Park | MSAT/SNP |
| QUBO_W_IMLS165 | Wild | Oak Mountain State Park | MSAT |
| QUBO_W_IMLS166 | Wild | Oak Mountain State Park | MSAT |
| QUBO_W_IMLS167 | Wild | Oak Mountain State Park | MSAT |
| QUBO_W_IMLS168 | Wild | Oak Mountain State Park | MSAT |
| QUBO_W_IMLS169 | Wild | Oak Mountain State Park | MSAT |
| QUBO_W_IMLS170 | Wild | Oak Mountain State Park | MSAT/SNP |
| QUBO_W_IMLS171 | Wild | Oak Mountain State Park | MSAT |
| QUBO_W_IMLS172 | Wild | Oak Mountain State Park | MSAT |
| QUBO_W_IMLS173 | Wild | Oak Mountain State Park | MSAT/SNP |
| QUBO_W_IMLS174 | Wild | Oak Mountain State Park | MSAT |
| QUBO_W_IMLS175 | Wild | Oak Mountain State Park | MSAT |
| QUBO_W_IMLS176 | Wild | Oak Mountain State Park | MSAT |
| QUBO_W_IMLS177 | Wild | Oak Mountain State Park | MSAT/SNP |
| QUBO_W_IMLS178 | Wild | Oak Mountain State Park | MSAT/SNP |
| QUBO_W_IMLS179 | Wild | Oak Mountain State Park | MSAT |
| QUBO_W_IMLS180 | Wild | Oak Mountain State Park | MSAT |
| QUBO_W_IMLS181 | Wild | Hinds Road | MSAT |
| QUBO_W_IMLS182 | Wild | Hinds Road | MSAT |
| QUBO_W_IMLS183 | Wild | Hinds Road | MSAT |
| QUBO_W_IMLS184 | Wild | Hinds Road | MSAT |
| QUBO_W_IMLS185 | Wild | Hinds Road | MSAT |
| QUBO_W_IMLS186 | Wild | Hinds Road | MSAT/SNP |
| QUBO_W_IMLS187 | Wild | Hinds Road | MSAT/SNP |
| QUBO_W_IMLS188 | Wild | Hinds Road | MSAT |
| QUBO_W_IMLS189 | Wild | Hinds Road | MSAT/SNP |
| QUBO_W_IMLS190 | Wild | Hinds Road | MSAT |
| QUBO_W_IMLS191 | Wild | Hinds Road | MSAT |
| QUBO_W_IMLS192 | Wild | Hinds Road | MSAT |
| QUBO_W_IMLS193 | Wild | Hinds Road | MSAT |
| QUBO_W_IMLS194 | Wild | Hinds Road | MSAT |
| QUBO_W_IMLS195 | Wild | Hinds Road | MSAT/SNP |
| QUBO_W_IMLS196 | Wild | Hinds Road | MSAT |
| QUBO_W_IMLS197 | Wild | Hinds Road | MSAT/SNP |
| QUBO_W_IMLS198 | Wild | Hinds Road | MSAT |
| QUBO_W_IMLS199 | Wild | Hinds Road | MSAT |
| QUBO_W_IMLS200 | Wild | Hinds Road | MSAT/SNP |
| QUBO_W_IMLS201 | Wild | Hinds Road | MSAT/SNP |
| QUBO_W_IMLS202 | Wild | Hinds Road | MSAT |
| QUBO_W_IMLS209 | Wild | Hinds Road | MSAT |
| QUBO_W_IMLS210 | Wild | Hinds Road | MSAT/SNP |
| QUBO_W_IMLS211 | Wild | Hinds Road | MSAT |
| QUBO_W_IMLS212 | Wild | Hinds Road | MSAT |
| QUBO_W_IMLS213 | Wild | Hinds Road | MSAT |
| QUBO_W_IMLS214 | Wild | Hinds Road | MSAT |
| QUBO_W_IMLS215 | Wild | Hinds Road | MSAT |
| QUBO_W_IMLS216 | Wild | Hinds Road | MSAT/SNP |
| QUBO_W_IMLS217 | Wild | Hinds Road | MSAT |
| QUBO_W_IMLS218 | Wild | Hinds Road | MSAT |
| QUBO_W_IMLS219 | Wild | Hinds Road | MSAT |
| QUBO_W_IMLS220 | Wild | Hinds Road | MSAT/SNP |
| QUBO_W_IMLS221 | Wild | Hinds Road | MSAT |
| QUBO_W_IMLS222 | Wild | Hinds Road | MSAT/SNP |
| QUBO_W_IMLS223 | Wild | Hinds Road | MSAT/SNP |
| QUBO_W_IMLS224 | Wild | Hinds Road | MSAT/SNP |
| QUBO_W_IMLS225 | Wild | Hinds Road | MSAT |
| QUBO_W_IMLS226 | Wild | Hinds Road | MSAT/SNP |
| QUBO_W_IMLS227 | Wild | Hinds Road | MSAT |
| QUBO_W_IMLS228 | Wild | Hinds Road | MSAT |
| QUBO_W_IMLS229 | Wild | Hinds Road | MSAT |
| QUBO_W_IMLS230 | Wild | Hinds Road | MSAT |
| QUBO_W_IMLS231 | Wild | Hinds Road | MSAT |
| QUBO_W_IMLS232 | Wild | Hinds Road | MSAT/SNP |
| QUBO_W_IMLS233 | Wild | Hinds Road | MSAT/SNP |
| QUBO_W_IMLS234 | Wild | Hinds Road | MSAT/SNP |
| QUBO_W_IMLS235 | Wild | Hinds Road | MSAT |
| QUBO_W_IMLS236 | Wild | Hinds Road | MSAT |
| QUBO_W_IMLS237 | Wild | Hinds Road | MSAT/SNP |
| QUBO_W_IMLS238 | Wild | Hinds Road | MSAT/SNP |
| QUBO_W_IMLS239 | Wild | Hinds Road | MSAT |
| QUBO_W_IMLS240 | Wild | Hinds Road | MSAT/SNP |
| QUBO_W_IMLS241 | Wild | Hinds Road | MSAT |
| QUBO_W_IMLS242 | Wild | Hinds Road | MSAT |
| QUBO_W_IMLS243 | Wild | Hinds Road | MSAT |
| QUBO_W_IMLS244 | Wild | Hinds Road | MSAT/SNP |
| QUBO_W_IMLS245 | Wild | Oak Mountain State Park | MSAT/SNP |
| QUBO_W_IMLS246 | Wild | Oak Mountain State Park | MSAT |
| QUBO_W_IMLS247 | Wild | Oak Mountain State Park | MSAT |
| QUBO_W_IMLS248 | Wild | Oak Mountain State Park | MSAT/SNP |
| QUBO_W_IMLS249 | Wild | Oak Mountain State Park | MSAT |
| QUBO_W_IMLS250 | Wild | Oak Mountain State Park | MSAT |
| **QUBO_W_IMLS251** | **Wild** | **Oak Mountain State Park** | **MSAT/SNP** |
| QUBO_W_IMLS252 | Wild | Oak Mountain State Park | MSAT |
| QUBO_W_IMLS253 | Wild | Oak Mountain State Park | MSAT/SNP |
| QUBO_W_IMLS254 | Wild | Oak Mountain State Park | MSAT |
| QUBO_W_IMLS255 | Wild | Oak Mountain State Park | MSAT |
| QUBO_W_IMLS256 | Wild | Oak Mountain State Park | MSAT |
| QUBO_W_IMLS257 | Wild | Oak Mountain State Park | MSAT |
| QUBO_W_IMLS258 | Wild | Oak Mountain State Park | MSAT/SNP |
| QUBO_W_IMLS259 | Wild | Oak Mountain State Park | MSAT |
| QUBO_W_IMLS260 | Wild | Oak Mountain State Park | MSAT/SNP |
| QUBO_W_IMLS261 | Wild | Oak Mountain State Park | MSAT/SNP |
| QUBO_W_IMLS262 | Wild | Oak Mountain State Park | MSAT/SNP |
| QUBO_W_IMLS263 | Wild | Oak Mountain State Park | MSAT |
| QUBO_W_IMLS264 | Wild | Oak Mountain State Park | MSAT/SNP |
| QUBO_W_IMLS265 | Wild | Oak Mountain State Park | MSAT |
| QUBO_W_IMLS266 | Wild | Peavine Falls | MSAT |
| QUBO_W_IMLS267 | Wild | Oak Mountain State Park | MSAT |
| QUBO_W_IMLS268 | Wild | Oak Mountain State Park | MSAT/SNP |
| QUBO_W_IMLS269 | Wild | Oak Mountain State Park | MSAT |
| QUBO_W_IMLS270 | Wild | Oak Mountain State Park | MSAT |
| QUBO_W_IMLS271 | Wild | Oak Mountain State Park | MSAT |
| QUBO_W_IMLS272 | Wild | Oak Mountain State Park | MSAT |
| QUBO_W_IMLS273 | Wild | Oak Mountain State Park | MSAT |
| QUBO_W_IMLS274 | Wild | Oak Mountain State Park | MSAT |
| QUBO_W_IMLS275 | Wild | Oak Mountain State Park | MSAT |
| QUBO_W_IMLS276 | Wild | Oak Mountain State Park | MSAT/SNP |
| QUBO_W_IMLS277 | Wild | Oak Mountain State Park | MSAT |
| QUBO_W_IMLS278 | Wild | Oak Mountain State Park | MSAT/SNP |
| QUBO_W_IMLS279 | Wild | Oak Mountain State Park | MSAT/SNP |
| QUBO_W_IMLS280 | Wild | Oak Mountain State Park | MSAT/SNP |
| QUBO_W_IMLS281 | Wild | Peavine Falls | MSAT |
| QUBO_W_IMLS282 | Wild | Peavine Falls | MSAT/SNP |
| QUBO_W_IMLS283 | Wild | Peavine Falls | MSAT/SNP |
| QUBO_W_IMLS284 | Wild | Peavine Falls | MSAT |
| QUBO_W_IMLS285 | Wild | Peavine Falls | MSAT/SNP |
| QUBO_W_IMLS286 | Wild | Peavine Falls | MSAT |
| QUBO_W_IMLS287 | Wild | Peavine Falls | MSAT |
| QUBO_W_IMLS288 | Wild | Peavine Falls | MSAT |
| QUBO_W_IMLS289 | Wild | Peavine Falls | MSAT |
| QUBO_W_IMLS290 | Wild | Peavine Falls | MSAT |
| QUBO_W_IMLS291 | Wild | Peavine Falls | MSAT |
| QUBO_W_IMLS292 | Wild | Peavine Falls | MSAT |
| QUBO_W_IMLS293 | Wild | Peavine Falls | MSAT/SNP |
| QUBO_W_IMLS294 | Wild | Peavine Falls | MSAT |
| QUBO_W_IMLS295 | Wild | Peavine Falls | MSAT |
| QUBO_W_IMLS296 | Wild | Peavine Falls | MSAT |
| QUBO_W_IMLS297 | Wild | Peavine Falls | MSAT |
| QUBO_W_IMLS298 | Wild | Peavine Falls | MSAT |
| QUBO_W_IMLS299 | Wild | Peavine Falls | MSAT |
| QUBO_W_IMLS300 | Wild | Peavine Falls | MSAT |
| QUBO_W_IMLS301 | Wild | Peavine Falls | MSAT |
| QUBO_W_IMLS302 | Wild | Peavine Falls | MSAT |
| QUBO_W_IMLS303 | Wild | Peavine Falls | MSAT |
| QUBO_W_IMLS304 | Wild | Peavine Falls | MSAT |
| QUBO_W_IMLS305 | Wild | Peavine Falls | MSAT |
| QUBO_W_IMLS306 | Wild | Peavine Falls | MSAT |
| QUBO_W_IMLS307 | Wild | Peavine Falls | MSAT/SNP |
| QUBO_W_IMLS308 | Wild | Population 11 | MSAT/SNP |
| QUBO_W_IMLS309 | Wild | Population 11 | MSAT/SNP |
| QUBO_W_IMLS310 | Wild | Population 11 | MSAT/SNP |
| QUBO_W_IMLS311 | Wild | Population 11 | MSAT/SNP |
| QUBO_W_IMLS312 | Wild | Population 11 | MSAT/SNP |

##### Table S2: Microsatellite and NextRAD samples of *Q. boyntonii*. Samples in bold were excluded from final analyses, either because they were duplicates, or because they generated comparatively lower numbers of reads (less than 1 million) than all other samples in the library.

##### [Fig_S1] Figure S1. Map of the sampling locations for wild *Q. acerifolia* and *Q. boyntonii* individuals.

## 1.2 Library preparation and sequencing

DNA extractions were performed on the 198 *Q. acerifolia* (100 garden, 98 wild) and 183 *Q. boyntonii* (86 garden, 97 wild) individuals utilized for NextRAD libraries using the Omega BioTek E.Z.N.A.® Plant DNA Kit, following a slightly modified protocol (see [Zumwalde et al., 2022](https://paperpile.com/c/vNTjrJ/ngMN)). Following extraction, DNA concentrations were analyzed using Qubit. Samples were standardized to volumes of 30 µL and 5 ng/µL per well, and gel electrophoresis was used to confirm the DNA quality of all samples. Samples were organized such that each sample type (*Q. acerifolia* and *Q. boyntonii*, garden and wild) was present on each plate, to minimize potential plate affects, and at least one duplicate sample and one blank was included on each plate.

Plates were shipped to SNPsaurus LLC (Eugene, Oregon) for final library preparation and sequencing using the NextRAD genotyping approach [(Russello et al., 2015](https://paperpile.com/c/vNTjrJ/Lt1wh)). Genomic DNA underwent fragmentation and adapter-ligation using Nextera reagent (Illumina, Inc). The Nextera reaction was scaled for fragmenting 12 ng of genomic DNA, although 36 ng of genomic DNA was used for input. Fragmented DNA with short adapter sequences was then amplified for 27 cycles at 74° C, with one of the primers matching the adapter and extending 10 nucleotides into genomic DNA with the selective sequence GTGTAGAGCC. This allows for only fragments starting with a sequence that can be hybridized by the selective sequence of the primer to be efficiently amplified. The NextRAD libraries were sequenced on a NovaSeq 6000 S4 lane with 150 bp paired-end reads at the University of Oregon.

## 1.3 Quality filtering

We used two programs to assess the quality of raw, demultiplexed NextRAD reads. First, reads were passed through the FastQC software [(Andrews, 2010)](https://paperpile.com/c/vNTjrJ/Mg8gj) to detect possible sequencing issues. Second, the Stacks process_radtags command was used to remove adapter sequences, drop low quality bases (within an Illumina quality score window), drop reads below 100 bp in length, and rename sequences. After the process_radtags command completed, we used the stacks-dist-extract process_radtags.log per_file_raw_read_counts to assess the total number of retained reads and the number of reads retained per sample, after filtering.

A total of 2,300,413,604 *Q. acerifolia* reads and 2,194,260,539 *Q. boyntonii* reads were retained, with an average of 11,618,251 *Q. acerifolia* reads retained per sample and 11,447,885 reads retained per sample for *Q. boyntonii*. Two samples (both *Q. boyntonii*, one garden and one wild) had significantly lower numbers of retained reads (less than 1 million) than the rest of our dataset; these samples also showed low numbers of reads using FastQC. Following recommendations from [Rivera-Colón & Catchen, 2022](https://paperpile.com/c/vNTjrJ/pGybb), these samples were removed from all downstream analyses.

Scripts for quality filtering can be found in the [filtering_QC](https://github.com/HobanLab/Morton_SSRvSNP_Empirical/tree/main/radAnalysis/filtering_QC) on the project’s GitHub repository.

## 1.4 Genotyping

### 1.4.1 Microsatellites

Details for the microsatellite genotyping of both *Q. acerifolia* and *Q. boyntonii* are provided below. More information for *Q. boyntonii* can also be found in [Hoban et al., 2020](https://paperpile.com/c/vNTjrJ/USN5) and [Spence et al., 2021](https://paperpile.com/c/vNTjrJ/R3dq). For both species, after PCR amplification, fragment analysis was performed using an ABI 3730 XL Genetic Capillary Electrophoresis Sequencer (Applied Biosystems), and fragments were sized using a Genescan LIZ 600. Fragment length was assessed and scored using the Geneious (v.10.2.3) software (Biomatters, Auckland, New Zealand).

#### 1.4.1.1 *Q. acerifolia*

We extracted DNA from all 463 *Quercus acerifolia* individuals (174 wild and 289 garden individuals) according to protocols described in [Zumwalde et al., 2022](https://paperpile.com/c/vNTjrJ/ngMN). PCRs were performed using 15 microsatellite markers designed for other red oak (Section *Lobatae*) species, using the protocols outlined in [Zumwalde et al., 2021](https://paperpile.com/c/vNTjrJ/EuvA). Alterations to microsatellite primers were made according to tables S3 and S4 below.

Individuals with missing data greater than 25% were removed from analyses, leaving 172 wild individuals and 277 garden individuals for genetic analyses.

| **Multiplex** | **Primer** | **Forward** | **Reverse** | **Source** |
| --- | --- | --- | --- | --- |
| MP1-1 | quru-GA-1G13 | AAAACTCACACAGCCGATTACTA | GATTCCATTGTCAACTGCGAAGA | [Aldrich et al., 2002](https://paperpile.com/c/vNTjrJ/FL1VN) |
|  | quru-GA-0C11 | ATACCCAGCTCCCATGACCA | TCCCCAAATTCAGGTAGTGT | [Aldrich et al., 2002](https://paperpile.com/c/vNTjrJ/FL1VN) |
| MP 2-1 | quru-GA-1F02 | CCAATCCACCCTTCCAAGTTCC | TGGTTGTTTTGCTTTATTCAGCC | [Aldrich et al., 2002](https://paperpile.com/c/vNTjrJ/FL1VN) |
|  | QU.G07 | GCCAACAAATTTAACTATCCAT | TAACTGGGCTAGATAATCAG | [Toppila, 2012](https://paperpile.com/c/vNTjrJ/sby8P) |
| MP3 | POR016 | AGCAACAGCAGAGCCAAAAT | CAGCGGCTTTGAGGTAATTC | [Sullivan et al., 2016](https://paperpile.com/c/vNTjrJ/F5IZu) |
|  | FIR013 | CGGGGAGGTTGATGAGTATT | AACACTGTCACCCCCATAGC | [Sullivan et al., 2016](https://paperpile.com/c/vNTjrJ/F5IZu) |
|  | GOT009 | CACCTCACTAAGCAACCTGTCA | TTTTGGAGGCGGAGATAATG | [Sullivan et al., 2016](https://paperpile.com/c/vNTjrJ/F5IZu) |
|  | FIR031 | ACGAGTCCAACGGAAGTTGT | CACAACTTCACAAGGCAAGG | [Sullivan et al., 2016](https://paperpile.com/c/vNTjrJ/F5IZu) |
|  | GOT040 | AAGGCACTCGTCGCTTTCTA | ACCGATTTGAAGCTCGAGAA | [Craft et al., 2007](https://paperpile.com/c/vNTjrJ/99By3) |
| MP4 | FIR043 | TTCTCCATTTCACACGCTTC | ACGACATCGTTTTGGAGCTT | [Sullivan et al., 2016](https://paperpile.com/c/vNTjrJ/F5IZu) |
|  | PIE039 | GAGCCTCTTTCATCGCTCAC | TCAACACCCCAAAACTCCAT | [Sullivan et al., 2016](https://paperpile.com/c/vNTjrJ/F5IZu) |
| MP5 | FIR053 | AGTTTCCCCACATTTGTTGC | TACCATGCACCAAGCAATTC | [Sullivan et al., 2016](https://paperpile.com/c/vNTjrJ/F5IZu) |
|  | FIR048 | TGCACCAAAATTGGAGGATG | TTGATGCAAGGTGCAGTTTC | [Sullivan et al., 2016](https://paperpile.com/c/vNTjrJ/F5IZu) |
|  | QpZag9 | GCAATTACAGGCTAGGCTGG | GTCTGGACCTAGCCCTCATG | [Steinkellner et al., 1997](https://paperpile.com/c/JemJQJ/LVaTj) |
|  | PIE125 | AATACAAATCGCAGGAGGTG | CTAACCCATCGTTCATGGAG | [Sullivan et al., 2016](https://paperpile.com/c/vNTjrJ/F5IZu) |

##### Table S3: *Q. acerifolia* microsatellite primer sequences. Multiplexes and the sources of primer sequences are included.

| **Multiplex** | **Primer** | **Color** | **Repeat** | **Size (bp)** |
| --- | --- | --- | --- | --- |
| MP1-1 | quru-GA-1G13 | NED | TC | 163 - 193 |
|  | quru-GA-0C11 | VIC | AG | 121- 238 |
| MP 2-1 | quru-GA-1F02 | VIC | TC | 149- 179 |
|  | QU.G07 | FAM | GA | 204 - 212 |
| MP3 | POR016 | VIC | GGT | 112 - 124 |
|  | FIR013 | NED | CAG | 135- 143 |
|  | GOT009 | FAM | TC | 225 - 251 |
|  | FIR031 | FAM | TC | 134 - 166 |
| MP4 | GOT040 | VIC | GA | 220 - 244 |
|  | FIR043 | VIC | TC | 106 - 138 |
|  | PIE039 | NED | GA | 153 - 173 |
| MP5 | FIR053 | FAM | GTG | 125 - 149 |
|  | FIR048 | VIC | CT | 180- 220 |
|  | QpZag9 | PET | GA | 139 - 163 |
|  | PIE125 | PET | GGAAGC | 240 - 276 |

##### Table S4: *Q. acerifolia* primer sizes.

#### 1.4.1.2 *Q. boyntonii*

DNA extraction steps for *Q. boyntonii* can be found in the supplement for [(Hoban et al., 2020)](https://paperpile.com/c/vNTjrJ/USN5). 322 individuals (245 wild, 77 garden) were genotyped using the markers specified below.

| **Multiplex** | **Primer** | **Forward** | **Reverse** | **Source** |
| --- | --- | --- | --- | --- |
| MP1 | QrZAG20 | CCATTAAAAGAAGCAGTATTTTGT | GCAACACTCAGCCTATATCTAGAA | [Kampfer et al., 1998](https://paperpile.com/c/vNTjrJ/hb5T) |
|  | QpZAG110 | GGAGGCTTCCTTCAACCTACT | GATCTCTTGTGTGCTGTATTT | [Steinkellner et al., 1997](https://paperpile.com/c/vNTjrJ/Zm0a) |
|  | QS00314 | TCAAAACGCAACGTTTCAAG | TTCGGGTTTTCTTTGTGGTC | [Chatwin et al., 2014](https://paperpile.com/c/vNTjrJ/Oan4) |
|  | QpZAG9 | GCAATTACAGGCTAGGCTGG | GTCTGGACCTAGCCCTCATG | [Steinkellner et al., 1997](https://paperpile.com/c/vNTjrJ/Zm0a) |
| MP2 | QS1904 | TCAGTCAAAAACCCACCTCC | GGGTTTTCTTCAGTTTGCTTGT | [Chatwin et al., 2014](https://paperpile.com/c/vNTjrJ/Oan4) |
|  | QS03297 | ACACAAAGAGCCATTCGCTT | GAGGCATACCTACGGGACAA | [Chatwin et al., 2014](https://paperpile.com/c/vNTjrJ/Oan4) |
|  | MSQ4 | TCTCCTCTCCCATAAACAGG | GTTCCTCTATCCAATCAGTAGTGAG | [Dow et al., 1995](https://paperpile.com/c/vNTjrJ/bAuD) |
| MP3 | QS00562 | ACCCCCACCTAATCCCAAC | ACCCCCACCTAATCCCAAC | [Chatwin et al., 2014](https://paperpile.com/c/vNTjrJ/Oan4) |
|  | QrZAG87 | TCCCACCACTTTGGTCTCTCA | GTTGTCAGCAGTGGGATGGGTA | [Kampfer et al., 1998](https://paperpile.com/c/vNTjrJ/hb5T) |
|  | QM69-2M1 | CACAATCTGCCCACATCATC | GGATGGACGAAGAGAAAGAT | [Isagi & Suhandono, 1997](https://paperpile.com/c/vNTjrJ/jS49) |

##### Table S5: *Q. boyntonii* microsatellite primer sequences. Multiplexes and the sources of primer sequences are included.

| **Multiplex** | **Primer** | **Color** | **Repeat** | **Size (bp)** |
| --- | --- | --- | --- | --- |
| MP1 | QrZAG20 | VIC | TC | 154 - 185 |
|  | QpZAG110 | NED | AG | 199 - 219 |
|  | QS00314 | PET | GAA | 166 - 210 |
|  | QpZAG9 | PET | AG | 230 - 273 |
| MP2 | QS1904 | FAM | TC | 134 - 159 |
|  | QS03297 | FAM | CA | 206 - 245 |
|  | MSQ4 | VIC | AG | 195 - 257 |
| MP3 | QS00562 | FAM | GA | 188 - 227 |
|  | QrZAG87 | NED | TC | 93 - 99 |
|  | QM69-2M1 | PET | (TGG)_6_(CGG)(TGG)_2_ | 220 -267 |

##### Table S6: *Q. boyntonii* primer sizes.

#### 1.4.1.3 Filtering

Microsatellite null alleles were detected using the Micro-checker software [(Van Oosterhout et al., 2004)](https://paperpile.com/c/vNTjrJ/9pi3) and the R package *PopGenReport* [(Adamack & Gruber, 2014)](https://paperpile.com/c/vNTjrJ/m23l). To mirror the approach taken for SNP loci, we deliberately chose to avoid filtering loci based on Hardy-Weinberg equilibrium, as we expect this to remove many loci from garden samples, which we wish to retain for our *ex situ* conservation analyses.

### 1.4.2 SNPs

#### 1.4.2.1 De novo assembly

To build an optimized *de novo* assembly, we chose a subset of garden and wild samples for both species (20 *Q. acerifolia* samples; 22 *Q. boyntonii* samples), ensuring the representation of each wild population for each species. Using these sample subsets, assemblies were built using the Stacks denovo_map.pl script, altering the values of the m, M, n, and gt-alpha parameters; ranges for each of these parameters are provided in Table S7.

| *Stacks parameter* | *Description* | *Stacks process* | *Possible values* |
| --- | --- | --- | --- |
| m | Minimum depth of coverage required to create a stack (a putative allele) | ustacks | 3 – 7 |
| M | Maximum distance (in nucleotides) allowed between stacks (putative alleles) to merge into a putative locus | ustacks | 1 – 8 |
| n | Number of mismatches allowed between sample loci when building the catalog | cstacks | Equivalent to M value |
| gt-alpha | Alpha threshold for calling genotypes | gstacks | 0.01, 0.05 |

##### Table S7: De novo assembly parameter optimization values. Every possible combination of the above parameters (80 total parameter value sets) was used to analyze a subset of samples (20 *Q. acerifolia* samples, 22 *Q. boyntonii* samples) for each species. The resulting dataset was then analyzed for four different assembly metrics as described in [Paris et al., 2017](https://paperpile.com/c/vNTjrJ/3V55Z), and the parameter values maximizing these metrics were chosen for building a finalized *de novo* assembly of all samples for the given species.

For each assembly, the metrics below were calculated using loci present in 80% of all samples (R80 loci), following [Paris et al., 2017](https://paperpile.com/c/vNTjrJ/3V55Z).

1. Unweighted coverage of each sample (for determining the *m* parameter)
2. Number of assembled loci
3. Number of polymorphic loci
4. Number of SNPs (across all assembled loci)

We found the combination of parameters which maximized these 4 assembly metrics for the sample subset of each species. Figures S2–S4 show assembly metrics across parameter values for *Q. acerifolia*; Figures S5–S7 show values for *Q. boyntonii*. When different values of the same parameter lead to comparable assembly metric values, we chose the lowest parameter value. After determining optimal parameter values, we used those values to build a *de novo* assembly for all samples of that species (Table S4). Scripts for parameter optimization and construction of optimized *de novo* assemblies can be found in the [denovoAssembly folder](https://github.com/HobanLab/Morton_SSRvSNP_Empirical/tree/main/radAnalysis/denovo) on the project’s GitHub repository.

##### [Fig_S2] Figure S2: Assembly metrics across different values of the Stacks m parameter for *Q. acerifolia* sequences. The m parameter specifies the minimum coverage depth required to create a stack (a putative allele), and is primarily used to determine the optimal depth of coverage assembly metric (seeking to maximize the value of coverage). The value of m which maximized depth of coverage, while maintaining a sufficient number of loci and SNPs, was selected for the optimized *de novo* assembly (for *Q. acerifolia*, m=7).

##### [Fig_S3] Figure S3: Assembly metrics across different values of the Stacks M/n parameters for *Q. acerifolia* sequences. The M parameter specifies the maximum number of nucleotides allowed to differ between stacks (putative alleles), while the n parameter specifies number of nucleotide mismatches allowed between putative loci in different samples when building the loci catalog. We specified matching M and n parameters, and selected the lowest value of M/n which maximized depth of coverage, number of assembled and polymorphic loci, and the number of SNPs for the optimized *de novo* assembly (for *Q. acerifolia*, M/n=4).

##### [Fig_S4] Figure S4: Assembly metrics across different values of the Stacks gt-alpha parameter for *Q. acerifolia* sequences. The gt-alpha parameter specifies alpha value threshold level for calling SNP genotypes, with lower values indicating a more stringent threshold and more strongly supported genotypes. Because the number of loci/SNPs generated at the more stringent gt-alpha value was still sufficient for the purposes of our study, we used a gt-alpha value of 0.01, to mitigate the impact of sequencing error on our NextRAD samples.

##### [Fig_S5] Figure S5: Assembly metrics across different values of the Stacks m parameter for *Q. boyntonii* sequences. The m parameter specifies the minimum coverage depth required to create a stack (a putative allele), and is primarily used to determine the optimal depth of coverage assembly metric (seeking to maximize the value of coverage). The value of m which maximized depth of coverage, while maintaining a sufficient number of loci and SNPs, was selected for the optimized *de novo* assembly (for *Q. boyntonii*, m=7).

##### [Fig_S6] Figure S6: Assembly metrics across different values of the Stacks M/n parameters for *Q. boyntonii* sequences. The M parameter specifies the maximum number of nucleotides allowed to differ between stacks (putative alleles), while the n parameter specifies number of nucleotide mismatches allowed between putative loci in different samples when building the loci catalog. We specified matching M and n parameters, and selected the lowest value of M/n which maximized depth of coverage, number of assembled and polymorphic loci, and the number of SNPs for the optimized *de novo* assembly (for *Q. boyntonii*, M/n=5).

##### [Fig_S7] Figure S7: Assembly metrics across different values of the Stacks gt-alpha parameter for *Q. boyntonii* sequences. The gt-alpha parameter specifies alpha value threshold level for calling SNP genotypes, with lower values indicating a more stringent threshold and more strongly supported genotypes. Because the number of loci/SNPs generated at the more stringent gt-alpha value was still sufficient for the purposes of our study, we used a gt-alpha value of 0.01, to mitigate the impact of sequencing error on our NextRAD samples.

| *De novo* assembly metrics | *Q. acerifolia* | *Q. boyntonii* |
| --- | --- | --- |
| m | 7 | 7 |
| M/n | 4 | 5 |
| gt-alpha | 0.01 | 0.01 |

##### Table S8: Optimized parameter values for *de novo* assemblies of *Q. acerifolia* and *Q. boyntonii*. These values were chosen to maximize coverage (particularly for the m value), the number of assembled loci, the number of polymorphic loci, and the number of SNPs.

#### 1.4.2.2 Reference alignment

To build reference alignment datasets, we aligned sequences cleaned using the Stacks process_radtags command to different reference genomes. *Q. boyntonii* samples were aligned to the *Q. robur* reference genome V2_2N [(Plomion et al., 2018)](https://paperpile.com/c/vNTjrJ/BWZsm). *Q. acerifolia* samples were aligned to a reference genome created for *Q. rubra* [(Kapoor et al., 2023)](https://paperpile.com/c/vNTjrJ/hE0K). We used the program GSNAP [(Wu & Nacu, 2010)](https://paperpile.com/c/vNTjrJ/v0iP7) to index both reference genomes and align our samples, using the “promiscuous” alignment parameters specified in [Paris et al., 2017](https://paperpile.com/c/vNTjrJ/3V55Z) but with fewer allowed mismatches (4 instead of 5). We used the samtools flagstat command [(Li et al., 2009)](https://paperpile.com/c/vNTjrJ/oyn7n) to assess the quality of reference alignments (Table S9). Because the *Q. acerifolia* and *Q. boyntonii* are separated by over 50 million years of evolution [(Hipp et al., 2020)](https://paperpile.com/c/JemJQJ/w4ymo), we chose to utilize two different reference genomes to optimize the reference alignment for each species separately. We utilized *Q. robur* for *Q. boyntonii* alignments instead of the *Q. lobata* reference genome [(Sork et al., 2022)](https://paperpile.com/c/JemJQJ/BR2u7) due to the closer relationship between *Q. robur* and *Q. boyntonii* [(Hipp et al., 2020)](https://paperpile.com/c/JemJQJ/w4ymo).

Alignments were then passed through the Stacks ref_map.pl script to generate loci datasets. As in *de novo* assemblies, we removed PCR duplicates and specified a gt-alpha value of 0.01. Results from the ref_map.pl script were assessed using the stacks-dist-extract command (bam_stats_per_sample values) to determine the percentage of reads retained in the ref_map.pl step. Scripts for alignment to reference genomes and genotype calling can be found in the [referenceAlignment folder](https://github.com/HobanLab/Morton_SSRvSNP_Empirical/tree/main/radAnalysis/reference) on GitHub.

| **Reference Alignment Results** | | |
| --- | --- | --- |
| *Retained Reads* | *Q. acerifolia* | *Q. boyntonii* |
| Average | 18,721,172 | 17,316,907 |
| Minimum | 3,813,519 | 4,077,839 |
| Maximum | 33,076,124 | 27,304,646 |

##### Table S9: Average, minimum, and maximum retained reads for *Q. acerifolia* and *Q. boyntonii* reference alignments. Values were assessed using samtools flagstat command. *Q. acerifolia* samples were aligned to a *Q. rubra* reference genome; *Q. boyntonii* samples were aligned to a *Q. robur* reference genome. Alignments were generated using the GSNP software.

## 1.5 Filtering and population assignment

We used the Stacks populations module to filter the loci and alleles in our SNP datasets (*de novo* and reference) prior to utilizing these loci for *ex situ* conservation and population analyses. We also utilized the popmap file passed to the Stacks populations module to group samples into specified populations. For *ex situ* conservation analyses (*ex situ* representation and resampling), we assigned samples to either the “garden” or “wild” populations, based on whether they were obtained from *ex situ* collections or in the wild. For population clustering analyses (STRUCTURE and DAPC) and F_ST_ calculations, we used the popmap file to only utilize wild samples and to group those wild samples into populations based on previous studies (for *Q. acerifolia*, see [Jerome et al., 2017](https://paperpile.com/c/vNTjrJ/hFZXg); for *Q. boyntonii*, see [Spence et al., 2021](https://paperpile.com/c/vNTjrJ/R3dq)).

We generated two datasets for each approach (*de novo* and reference) using different values for the -R parameter, which specifies the minimum percentage of individuals in which a locus must be present to be included in the dataset. We generated datasets using values of R0 (no filter on missing data) and R80 (loci present in less than 80% of all samples are dropped), applying these filters to both *de novo* and reference datasets. We used the --write-single-snp flag to partially account for linkage between proximate SNP loci, and to ensure replicability of our *ex situ* conservation findings.  While we did not calculate estimates for linkage disequilibrium decay, we expect linkage to decline very rapidly in these *Quercus* species, as has been demonstrated in other long-lived, wind-pollinated tree species [(Ingvarsson, 2005; Plomion et al., 2014; Silva-Junior & Grattapaglia, 2015)](https://paperpile.com/c/JemJQJ/0LuL+5Etu+M4W5).

Our preliminary analyses utilized a value for the --min-maf flag (minimum minor allele frequency), which often led to unexpectedly high levels of *ex situ* representation of rare (less than 1% frequency in the wild population) alleles. Therefore, to avoid the biasing effect of this flag, we chose to specify no filter for minor alleles (either by minor allele frequency or by minor allele count).

For *Q. acerifolia*, wild individuals from the Kessler Mountain were included in sequencing and SNP calling steps (*de novo* assembly and reference alignment). However, recent analyses have shown this population to likely be made up of hybrids between *Q. acerifolia* and *Q. shumardii* ([Wu et al., 2023](https://paperpile.com/c/AFE7Nm/c4VK4), Table S1). Therefore, these samples were excluded from downstream *ex situ* conservation and population clustering analyses by removing them from the population map provided to the Stacks populations module. Similarly, we used microsatellite datasets for *Q. acerifolia* that excluded Kessler individuals for the analyses described below.

The .genepop and .structure files generated by the Stacks populations module were used in the downstream analyses for *ex situ* conservation, population genetic statistics, and population clustering.

## 1. 6 *Ex situ* conservation analyses

Table S10 summarizes the datasets used for both the *ex situ* conservation and population clustering analyses, including whether only wild or garden and wild samples were utilized for the analysis.

| Analyses | | **Microsatellite** | | | | **SNP** | | | | | | | |
| --- | --- | --- | --- | --- | --- | --- | --- | --- | --- | --- | --- | --- | --- |
|  |  |  |  |  |  | *De novo* assembly/Reference alignment | | | | | | | |
|  |  |  |  |  |  | *R0* | | | | *R80* | | | |
|  |  | Garden and Wild | | Wild Only | | Garden and Wild | | Wild Only | | Garden and Wild | | Wild Only | |
|  |  | C | S | C | S | C | S | C | S | C | S | C | S |
| *Ex situ* conservation | *Ex situ* representation | ✓ | ✓ | X | X | ✓ | ✓ | X | X | ✓ | ✓ | X | X |
|  | Resampling | X | X | ✓ | ✓ | X | X | ✓ | ✓ | X | X | ✓ | ✓ |
| Population clustering | Statistics (A_R_, H_e_, F_ST_) | ✓ | ✓ | ✓ | ✓ | ✓ | ✓ | ✓ | ✓ | ✓ | ✓ | ✓ | ✓ |
|  | STRUCTURE | X | ✓ | X | ✓ | X | X | X | X | X | ✓ | X | ✓ |
|  | DAPC | X | X | X | ✓ | X | X | X | X | X | X | X | ✓ |

##### Table S10: D*atasets used in the ex situ conservation and population analyses of Quercus acerifolia and Q. boyntonii. “C” stands for “Complete” datasets, which contain all samples included in original analyses (for both microsatellite and SNP analyses); “S” stands for “Subset” datasets, which only contain samples shared between microsatellite and SNP analyses. Missing data filter values refer to datasets using all SNP loci generated by an approach (R0) or only loci shared by 80% of samples (R80). For ex situ conservation analyses, only wild samples were utilized for resampling analyses to determine minimum sample size estimates, while garden and wild samples were used to determine ex situ representation of wild allelic diversity. For population analyses, R80 datasets were utilized to ease computational processing. “DAPC” stands for Discriminant Analysis of Principal Components. Cells highlighted in orange indicate datasets that were generated for only a single species (Q. acerifolia).*

## 1.7 Population clustering analyses

### 1.7.1 STRUCTURE

The software STRUCTURE [(Pritchard et al., 2000)](https://paperpile.com/c/vNTjrJ/96Nd8) was used to group individuals into putative source populations, in order to assess how microsatellite and SNP markers impacted population assignment.

STRUCTURE assigns individuals to one or more of K source populations using Bayesian clustering by assuming loci to be in Hardy-Weinberg proportions and linkage equilibrium. To directly compare results from different marker types, we clustered only wild samples shared between our microsatellite and SNP datasets (“Subset” datasets), and for computational feasibility, we only used R80 loci from SNP datasets.

STRUCTURE input files were generated using the Stacks populations module. For both species we ran STRUCTURE 20 times for each K value ranging from 2 to 7, using 100,000 burn-in iterations and 100,000 MCMC reps. We used the default admixture (0, no admixture) and allele frequency models (λ=1), and default values for all other parameters. To generate STRUCTURE input files for subset microsatellite datasets, we used the genind2structure function [(Clark, 2017)](https://paperpile.com/c/vNTjrJ/iZdp).

To summarize STRUCTURE results across repeated runs and K values, we used the “Main pipeline” module of the CLUMPAK server [(Kopelman et al., 2015)](https://paperpile.com/c/vNTjrJ/paMu5) to generate CLUMPP [(Jakobsson & Rosenberg, 2007)](https://paperpile.com/c/vNTjrJ/fqMNx) files, which were subsequently visualized using a [custom R script](https://github.com/HobanLab/Morton_SSRvSNP_Empirical/blob/main/popAnalysis/STRUCTURE/structure_PlottingMS.R). Additionally, the “Best K” CLUMPAK module was used to determine optimal K values using the Evanno method [(Evanno et al., 2005)](https://paperpile.com/c/vNTjrJ/jxbQ6) and the K value with the greatest likelihood, as described in the STRUCTURE manual [(Pritchard et al., 2010)](https://paperpile.com/c/vNTjrJ/YDClX).

For our *Q. acerifolia* reference analysis, R80 datasets contained many loci (15,531), which limited our downstream computation abilities using STRUCTURE and CLUMPAK. To address this, we generated a “whitelist” of 5,000 randomly selected loci from the entire R80 dataset using the Stacks populations module, and used these loci to generate a STRUCTURE input file. These whitelisted loci were only used for STRUCTURE comparisons, and not for any other analysis (DAPC, F_ST_, or *ex situ* representation analyses).

#### Garden provenance assessments

In addition to comparing overall clustering patterns in both species, we used STRUCTURE to cluster *Q. acerifolia* garden and wild samples from both microsatellite and SNP R80 datasets, in order to assess whether marker type impacted garden sample assignment back to wild source populations. To do so, we generated STRUCTURE input files using the Stacks populations module, including both garden and wild samples in the popmap file provided to the module. As in the analyses described above, we ran STRUCTURE 50 times for each K value ranging from 2 to 7, with 10,000 burn-in iterations and 20,000 MCMC reps, using the default admixture and allele frequency models and default values for all other parameters. We used CLUMPAK to summarize results across repeated runs and K values. For SNP Reference datasets, we again utilized a whitelist of 5,000 randomly selected loci.

### 1.7.2 Discriminant Analysis of Principal Components

Discriminant Analysis of Principal Components (DAPC; [Jombart et al., 2010)](https://paperpile.com/c/vNTjrJ/mHWZN) was used in addition to STRUCTURE to cluster samples into groups. DAPC is a statistical technique designed to accommodate the large sizes of NGS datasets by maximizing between-group genomic variation and minimizing within-group genomic variation. Genomic data is first transformed using a PCA, and then a k-means clustering algorithm is run and Bayesian Information Criterion (BIC) scores are estimated for each value of K clusters (usually interpreted as populations). The best supported number of clusters (that with the lowest BIC score) is used to map individuals in a Principal Components Analysis (PCA) format.

Using the *adegenet* package [(Jombart, 2008; Jombart & Ahmed, 2011)](https://paperpile.com/c/AFE7Nm/Q6dQR+Vg2lt), we ran DAPC in R by analyzing the .genepop files generated by the Stacks populations module. As in our STRUCTURE analyses, we compared markers using only wild samples shared between microsatellite and SNP datasets (“Subset” samples), and utilized R80 loci for SNP datasets.

# Section 2. Results

[Fig_S8]

##### Figure S8: Allele frequency histograms for Subset *Q. acerifolia* microsatellite, SNP *de novo* (R80), and SNP reference (R80)*.* Only alleles present in wild individuals are shown. Note that the y-axis scales are different for each marker type.

[Fig_S9]

## Figure S9: Allele frequency histograms for Subset *Q. boyntonii* microsatellite, SNP *de novo* (R80), and SNP reference (R80)*.* Only alleles present in wild individuals are shown. Note that the y-axis scales are different for each marker type.

## 2.1 *Ex situ* conservation

Results from *ex situ* representation and resampling analyses for *Q. acerifolia* and *Q. boyntonii* are provided below.

| ***Quercus acerifolia*: *ex situ* representation** | | | | | | |
| --- | --- | --- | --- | --- | --- | --- |
|  |  | Percentage  (Raw counts) | | | | |
|  | Data type  (sample size) | *Very common (>10%)* | *Common  (>5%)* | *Low frequency  (1% – 10%)* | *Rare  (<1%)* | *Total  (>0%)* |
| *Complete* | Microsatellites  (277 Garden, 164 Wild) | 100.00% (42/42) | 100.00% (71/71) | 98.53% (67/68) | 82.61% (19/23) | 96.24%  (128/133) |
|  | SNPs: *De novo*, R0  (96 Garden, 91 Wild) | 99.82% (46,101/46,183) | 97.97% (70,880/72,352) | 74.77% (98,472/131,697) | 42.26% (10,656/25,214) | 76.43%  (155,229/203,094) |
|  | SNPs: *De novo*, R80  (96 Garden, 91 Wild) | 100.00% (5,986/5,986) | 99.91% (6,328/6,334) | 73.28% (1,892/2,582) | 41.06% (560/1,364) | 84.96%  (8,438/9,932) |
|  | SNPs: Reference, R0  (96 Garden, 91 Wild) | 99.93% (51,147/51,181) | 98.73% (80,420/81,458) | 89.37% (118,126/132,177) | 51.02% (12,122/23,760) | 87.58%  (181,395/207,118) |
|  | SNPs: Reference, R80  (96 Garden, 91 Wild) | 100.00% (15,565/15,565) | 99.93% (16,046/16,058) | 67.01% (3,983/5,944) | 43.99% (1,796/4,083) | 83.40%  (21,344/25,592) |
| *Subset* | Microsatellites  (88 Garden, 91 Wild) | 100.00% (40/40) | 100.00% (73/73) | 92.00% (69/75) | 90.00% (9/10) | 94.40%  (118/125) |
|  | SNPs: *De novo*, R0  (88 Garden, 91 Wild) | 99.79% (46,084/46,183) | 97.72% (70,705/72,352) | 73.36% (96,619/131,697) | 40.71% (10,264/25,214) | 75.32%  (152,967/203,094) |
|  | SNPs: *De novo*, R80  (88 Garden, 91 Wild) | 99.98% (5,985/5,986) | 99.87% (6,326/6,334) | 72.27% (1,866/2,582) | 39.74% (542/1,364) | 84.50%  (8,393/9,932) |
|  | SNPs: Reference, R0  (88 Garden, 91 Wild) | 99.92% (51,138/51,181) | 98.56% (80,283/81,458) | 88.32% (116,739/132,177) | 49.44% (11,748/23,760) | 86.73%  (179,625/207,118) |
|  | SNPs: Reference, R80  (88 Garden, 91 Wild) | 100.00% (15,565/15,565) | 99.92% (16,045/16,058) | 65.85% (3,914/5,944) | 43.03% (1,757/4,083) | 82.98% (21,236/25,592) |

Table S11: Raw *ex situ* representation values for *Quercus acerifolia* across marker types. Values represent allelic diversity represented in different categories of alleles based on their frequency in wild samples, as well as the sum across all frequency categories (“Total”). Rows indicate different marker types and processing approaches for SNP markers (de novo assembly and reference alignment). R values indicate the missing data level (R0: no filter on missing data; R80: loci present in at least 80% of all samples). “Complete” values indicate all samples for each marker type; “Subset” values indicate only samples shared across microsatellite and SNP datasets. Values in parentheses show the number of garden and wild samples for each dataset.

| ***Quercus boyntonii*: *ex situ* representation** | | | | | | |
| --- | --- | --- | --- | --- | --- | --- |
|  |  | Percentage  (Raw counts) | | | | |
|  | Data type  (sample size) | *Very common (>10%)* | *Common  (>5%)* | *Low frequency  (1% – 10%)* | *Rare  (<1%)* | *Total  (>0%)* |
| *Complete* | Microsatellites  (77 Garden, 245 Wild) | 100.00% (19/19) | 100.00% (36/36) | 66.20% (47/71) | 31.71% (13/41) | 60.31% (79/131) |
|  | SNPs: *De novo*, R0  (85 Garden, 95 Wild) | 99.67% (50,372/50,540) | 96.94% (81,519/84,088) | 66.55% (118,600/178,216) | 36.24% (11,286/31,143) | 69.46% (181,113/260,762) |
|  | SNPs: *De novo*, R80  (85 Garden, 95 Wild) | 99.98% (5,476/5,477) | 99.84% (5,762/5,771) | 66.44% (1,651/2,485) | 28.19% (528/1,873) | 77.87% (7,671/9,851) |
|  | SNPs: Reference, R0  (85 Garden, 95 Wild) | 99.88% (26,315/26,347) | 98.21% (44,023/44,824) | 86.42% (65,558/75,863) | 44.59% (6,370/14,286) | 84.37% (98,555/116,809) |
|  | SNPs: Reference, R80  (85 Garden, 95 Wild) | 100.00% (6,348/6,348) | 99.80% (6,521/6,534) | 59.71% (1,559/2,611) | 31.79% (763/2,400) | 76.34% (8,677/11,366) |
| *Subset* | Microsatellites  (75 Garden, 94 Wild) | 100.00% (20/20) | 97.14% (34/35) | 69.12% (47/68) | 16.67% (3/18) | 66.04% (70/106) |
|  | SNPs: *De novo*, R0  (75 Garden, 94 Wild) | 99.59% (50,784/50,991) | 96.43% (80,456/83,525) | 64.21% (114,139/177,767) | 34.02% (10,581/31,103) | 67.54% (175,504/259,861) |
|  | SNPs: *De novo*, R80  (75 Garden, 94 Wild) | 99.98% (5,490/5,491) | 99.83% (5,757/5,767) | 63.99% (1,580/2,469) | 26.25% (492/1,874) | 76.90% (7,562/9,834) |
|  | SNPs: Reference, R0  (75 Garden, 94 Wild) | 99.80% (26,404/26,457) | 97.79% (43,515/44,500) | 84.52% (64,040/75,765) | 42.35% (6,063/14,316) | 82.81% (96,507/116,538) |
|  | SNPs: Reference, R80  (75 Garden, 94 Wild) | 100.00% (6,354/6,354) | 99.77% (6,514/6,529) | 56.73% (1,475/2,600) | 29.87% (710/2,377) | 75.36% (8,539/11,331) |

Table S12: Raw *ex situ* representation values for *Quercus boyntonii* across marker types. Values represent allelic diversity represented in different categories of alleles based on their frequency in wild samples, as well as the sum across all frequency categories (“Total”). Rows indicate different marker types and processing approaches for SNP markers (*de novo* assembly and reference alignment). R values indicate the missing data level (R0: no filter on missing data; R80: loci present in at least 80% of all samples). “Complete” values indicate all samples for each marker type; “Subset” values indicate only samples shared across microsatellite and SNP datasets. Values in parentheses show the number of garden and wild samples for each dataset.

### 2.1.2 Resampling

The plots below show resampling curves for Complete and Subset microsatellite and SNP datasets, for both species. An explanation of resampling plots can be found in the description for Figure S10.

[Fig_S10]

##### Figure S10: Resampling curves for Complete *Q. acerifolia* microsatellite dataset*.* Points reflect the average allelic representation of the number of wild individuals randomly selected from the entire dataset, across 5,000 replicates. Different colors indicate alleles of different frequencies, with red points indicating the Total allelic representation (alleles of all subcategories). The horizontal dotted line represents the 95% threshold, maintained as a standard for *ex situ* collections, and the solid vertical line represents the number of samples (on average) required to reach that threshold.

[Fig_S11]

##### Figure S11: Resampling curves for Subset *Q. acerifolia* microsatellite dataset*.*

[Fig_S12]

##### Figure S12: Resampling curves for Complete *Q. acerifolia* SNP (*de novo*) datasets (R0 and R80)*.*

[Fig_S13]

##### Figure S13: Resampling curves for Subset *Q. acerifolia* SNP (*de novo*) datasets (R0 and R80)*.* Because all *Q. acerifolia* wild samples in the SNP analysis are also included in the microsatellite analysis, sample sets are identical between the Complete and Subset datasets.

[Fig_S14]

##### Figure S14: Resampling curves for Complete *Q. acerifolia* SNP (reference) datasets (R0 and R80)*.*

[Fig_S15]

##### Figure S15: Resampling curves for Subset *Q. acerifolia* SNP (reference) datasets (R0 and R80)*.* Because all *Q. acerifolia* wild samples in the SNP analysis are also included in the microsatellite analysis, sample sets are identical between the Complete and Subset datasets.

[Fig_S16]

##### Figure S16: Resampling curves for Complete *Q. boyntonii* microsatellite dataset*.*

[Fig_S17]

##### Figure S17: Resampling curves for Subset *Q. boyntonii* microsatellite dataset*.*

[Fig_S18]

##### Figure S18: Resampling curves for Complete *Q. boyntonii* SNP (*de novo*) datasets (R0 and R80)*.*

[Fig_S19]

##### Figure S19: Resampling curves for Subset *Q. boyntonii* SNP (*de novo*) datasets (R0 and R80)*.*

[Fig_S20]

##### Figure S20: Resampling curves for Complete *Q. boyntonii* SNP (reference) datasets (R0 and R80)*.*

[Fig_S21]

##### Figure S21: Resampling curves for Subset *Q. boyntonii* SNP (reference) datasets (R0 and R80)*.*

## 2.2 Population clustering analyses

Results for STRUCTURE and DAPC analyses are provided below. Population genetic statistics for both species are provided in the main text.

### 2.2.1 STRUCTURE

[Fig_S22]

##### Figure S22: STRUCTURE plots for MSAT, SNP *De novo*, and SNP Reference wild *Q. acerifolia* datasets. SNP datasets are generated using R80 loci. Results shown are the “Major Cluster'' outputs (the most common mode detected by CLUMPP, out of 50 replicate runs of STRUCTURE). MSAT stands for “microsatellite”.

[Fig_S23]

##### Figure S23: STRUCTURE plots for MSAT, SNP *De novo*, and SNP Reference wild *Q. boyntonii* datasets. SNP datasets are generated using R80 loci. Results shown are the “Major Cluster” outputs (the most common mode out of 50 replicate STRUCTURE runs). Site collection names, as outlined in [Hoban et al., 2020](https://paperpile.com/c/vNTjrJ/USN5) Supplementary materials, are indicated along the bottom*.* MSAT stands for “microsatellite”.

|  | ***Q. acerifolia*** | | ***Q. boyntonii*** | |
| --- | --- | --- | --- | --- |
| *Marker (Approach)* | *Evanno Best K* | *Max. Likelihood K* | *Evanno Best K* | *Max. Likelihood K* |
| Microsatellite | 5 | 6 | 6 | 2 |
| SNP (*De novo* assembly) | 6 | 6 | 5 | 2 |
| SNP (Reference alignment) | 4 | 4 | 5 | 2 |

##### Table S13: “Optimal” K values determined using STRUCTURE and CLUMPAK, for *Q. acerifolia* and *Q.* *boyntonii*. Values for two metrics are shown: the optimal K value determined by the Evanno method [(Evanno et al., 2005)](https://paperpile.com/c/vNTjrJ/jxbQ6) and the maximum likelihood K value, as described in [Pritchard et al., 2010](https://paperpile.com/c/vNTjrJ/YDClX).

[Fig_S24]

##### Figure S24: STRUCTURE plots across marker types for garden and wild *Q. acerifolia* individuals, at K=4 clusters. “W” indicates wild samples, and “G” indicates garden samples; the last 5 samples are garden samples with unknown provenances. Samples are organized according to associated populations. Values shown are the major clusters identified by CLUMPP after 50 replicate STRUCTURE runs, and SNP datasets (de novo and reference) are processed using R80 loci. K=4 values are used to better assess garden provenances. MSAT stands for “microsatellite”

### 2.2.2 DAPC

[Fig_S25]

##### Figure S25: DAPC plot of QUAC Wild individuals generated using microsatellite (MSAT) loci, visualized using the optimal number of clusters (K=4). Points represent individuals, and colors correspond to the geographic areas (mountaintops) from which wild individuals were sampled. Axes represent discriminant functions of synthetic variables built from loci. “Subset” samples are used to enable comparisons across marker types.

[[Fig_S26](https://drive.google.com/open?id=1mhZljwYt1TJUgAY-PdB801nWb3BaUddL&usp=drive_copy)]

##### Figure S26: DAPC plot of QUAC Wild individuals generated using SNP (*de novo*) loci, visualized using the optimal number of clusters (K=2). “Subset” samples are used to enable comparisons across marker types (see Table S6).

[Fig_S27]

##### Figure S27: DAPC plot of QUAC Wild individuals generated using SNP (reference) loci, visualized using the optimal number of clusters (K=2). “Subset” samples are used to enable comparisons across marker types (see Table S6).

[Fig_S28]

##### Figure S28: DAPC plot of QUBO Wild individuals generated using microsatellite (MSAT) loci, visualized using the optimal number of clusters (K=3). Several individuals from several populations (described in [Spence et al., 2021](https://paperpile.com/c/vNTjrJ/R3dq) and [Hoban et al., 2020](https://paperpile.com/c/vNTjrJ/USN5)) were separated into distinct clusters determined in the k-means clustering step. OMSP stands for “Oak Mountain State Park”.

[Fig_S29]

##### Figure S29: DAPC plot of QUBO Wild individuals generated using SNP (*de novo*) loci, visualized using the optimal number of clusters (K=2).

[Fig_S30]

##### Figure S30: DAPC plot of QUBO Wild individuals generated using SNP (reference) loci, visualized using the optimal number of clusters (K=2).

# Section 3. References

[Adamack, A. T., & Gruber, B. (2014). PopGenReport: simplifying basic population genetic analyses in R. *Mol Ecol*. https://doi.org/](http://paperpile.com/b/vNTjrJ/m23l)[10.1111/2041-210X.12158](http://dx.doi.org/10.1111/2041-210X.12158)

[Aldrich, P. R., Michler, C. H., Sun, W., & Romero-Severson, J. (2002). Microsatellite markers for northern red oak (Fagaceae: *Quercus rubra*). *Molecular Ecology Notes*, *2*(4), 472–474.](http://paperpile.com/b/vNTjrJ/FL1VN)

[Andrews, S. (2010). *FastQC: A Quality Control Tool for High Throughput Sequence Data* [Online].](http://paperpile.com/b/vNTjrJ/Mg8gj) <http://www.bioinformatics.babraham.ac.uk/projects/fastqc/>

[Chatwin, W. B., Carpenter, K. K., Jimenez, F. R., Elzinga, D. B., Johnson, L. A., & Maughan, P. J. (2014). Microsatellite primer development for post oak, *Quercus stellata* (Fagaceae). *Applications in Plant Sciences*, *2*(10). https://doi.org/](http://paperpile.com/b/vNTjrJ/Oan4)[10.3732/apps.1400070](http://dx.doi.org/10.3732/apps.1400070)

[Clark, L. (2017). *lvclark/R_genetics_conv: R_genetics_conv 1.1*. https://doi.org/](http://paperpile.com/b/vNTjrJ/iZdp)[10.5281/zenodo.846816](http://dx.doi.org/10.5281/zenodo.846816)

[Craft, K. J., Owens, J. D., & Ashley, M. V. (2007). Application of plant DNA markers in forensic botany: genetic comparison of *Quercus* evidence leaves to crime scene trees using microsatellites. *Forensic Science International*, *165*(1), 64–70.](http://paperpile.com/b/vNTjrJ/99By3)

[Dow, B. D., Ashley, M. V., & Howe, H. F. (1995). Characterization of highly variable (GA/CT)_n_ microsatellites in the bur oak, *Quercus macrocarpa*. *TAG. Theoretical and Applied Genetics. Theoretische Und Angewandte Genetik*, *91*(1), 137–141.](http://paperpile.com/b/vNTjrJ/bAuD)

[Evanno, G., Regnaut, S., & Goudet, J. (2005). Detecting the number of clusters of individuals using the software STRUCTURE: a simulation study. *Molecular Ecology*, *14*(8), 2611–2620.](http://paperpile.com/b/vNTjrJ/jxbQ6)

[Hipp, A. L., Manos, P. S., Hahn, M., Avishai, M., Bodénès, C., Cavender-Bares, J., Crowl, A. A., Deng, M., Denk, T., Fitz-Gibbon, S., Gailing, O., González-Elizondo, M. S., González-Rodríguez, A., Grimm, G. W., Jiang, X. L., Kremer, A., Lesur, I., McVay, J. D., Plomion, C.,  Valencia-Avalos, S. (2020). Genomic landscape of the global oak phylogeny. *The New Phytologist*, *226*(4), 1198–1212.](http://paperpile.com/b/JemJQJ/w4ymo)

[Hoban, S., Callicrate, T., Clark, J., Deans, S., Dosmann, M., Fant, J., Gailing, O., Havens, K., Hipp, A. L., Kadav, P., Kramer, A. T., Lobdell, M., Magellan, T., Meerow, A. W., Meyer, A., Pooler, M., Sanchez, V., Spence, E., Thompson, P., Griffith, M. P. (2020). Taxonomic similarity does not predict necessary sample size for ex situ conservation: a comparison among five genera. *Proceedings. Biological Sciences / The Royal Society*, *287*(1926), 20200102.](http://paperpile.com/b/vNTjrJ/USN5)

[Ingvarsson, P. K. (2005). Nucleotide polymorphism and linkage disequilibrium within and among natural populations of European aspen (*Populus tremula* L., Salicaceae). *Genetics*, *169*(2), 945–953.](http://paperpile.com/b/JemJQJ/0LuL)

[Isagi, Y., & Suhandono, S. (1997). PCR primers amplifying microsatellite loci of *Quercus myrsinifolia* Blume and their conservation between oak species. *Molecular Ecology*, *6*(9), 897–899.](http://paperpile.com/b/vNTjrJ/jS49)

[Jakobsson, M., & Rosenberg, N. A. (2007). CLUMPP: a cluster matching and permutation program for dealing with label switching and multimodality in analysis of population structure. *Bioinformatics* , *23*(14), 1801–1806.](http://paperpile.com/b/vNTjrJ/fqMNx)

[Jerome, D., Beckman, E., Kenny, L., Wenzell, K., Chai-Shian, K., & Westwood, M. (2017). The red list of US oaks. In *The red list of US oaks.* Morton Arboretum.](http://paperpile.com/b/vNTjrJ/hFZXg)

[Jombart, T. (2008). adegenet: a R package for the multivariate analysis of genetic markers. *Bioinformatics* , *24*(11), 1403–1405.](http://paperpile.com/b/AFE7Nm/Q6dQR)

[Jombart, T., Devillard, S., & Balloux, F. (2010). Discriminant analysis of principal components: a new method for the analysis of genetically structured populations. *BMC Genetics*, *11*, 94.](http://paperpile.com/b/vNTjrJ/mHWZN)

[Jombart, T., & Ahmed, I. (2011). adegenet 1.3-1: new tools for the analysis of genome-wide SNP data. *Bioinformatics*, *27*(21), 3070–3071.](http://paperpile.com/b/AFE7Nm/Vg2lt)

[Kampfer, S., Lexer, C., Glössl, J., & Steinkellner, H. (1998). Characterization of (GA)_n_ microsatellite loci from Quercus robur. *Hereditas*, *129*(2), 183–186.](http://paperpile.com/b/vNTjrJ/hb5T)

[Kapoor, B., Jenkins, J., Schmutz, J., Zhebentyayeva, T., Kuelheim, C., Coggeshall, M., Heim, C., Lasky, J. R., Leites, L., Islam-Faridi, N., Romero-Severson, J., DeLeo, V. L., Lucas, S. M., Lazic, D., Gailing, O., Carlson, J., & Staton, M. (2023). A haplotype-resolved chromosome-scale genome for *Quercus rubra* L. provides insights into the genetics of adaptive traits for red oak species. *G3* . https://doi.org/](http://paperpile.com/b/vNTjrJ/hE0K)[10.1093/g3journal/jkad209](http://dx.doi.org/10.1093/g3journal/jkad209)

[Kopelman, N. M., Mayzel, J., Jakobsson, M., Rosenberg, N. A., & Mayrose, I. (2015). CLUMPAK: a program for identifying clustering modes and packaging population structure inferences across K. *Molecular Ecology Resources*, *15*(5), 1179–1191.](http://paperpile.com/b/vNTjrJ/paMu5)

[Li, H., Handsaker, B., Wysoker, A., Fennell, T., Ruan, J., Homer, N., Marth, G., Abecasis, G., Durbin, R., & 1000 Genome Project Data Processing Subgroup. (2009). The Sequence Alignment/Map format and SAMtools. *Bioinformatics*, *25*(16), 2078–2079.](http://paperpile.com/b/vNTjrJ/oyn7n)

[Paris, J. R., Stevens, J. R., & Catchen, J. M. (2017). Lost in parameter space: a road map for Stacks. *Methods in Ecology and Evolution / British Ecological Society*, *8*(10), 1360–1373.](http://paperpile.com/b/vNTjrJ/3V55Z)

[Plomion, C., Chancerel, E., Endelman, J., Lamy, J.B., Mandrou, E., Lesur, I., Ehrenmann, F., Isik, F., Bink, M. C. A. M., van Heerwaarden, J., & Bouffier, L. (2014). Genome-wide distribution of genetic diversity and linkage disequilibrium in a mass-selected population of maritime pine. *BMC Genomics*, *15*, 171.](http://paperpile.com/b/JemJQJ/5Etu)

[Plomion, C., Aury, J. M., Amselem, J., Leroy, T., Murat, F., Duplessis, S., Faye, S., Francillonne, N., Labadie, K., Le Provost, G., Lesur, I., Bartholomé, J., Faivre-Rampant, P., Kohler, A., Leplé, J. C., Chantret, N., Chen, J., Diévart, A., Alaeitabar, T., Salse, J. (2018). Oak genome reveals facets of long lifespan. *Nature Plants*, *4*(7), 440–452.](http://paperpile.com/b/vNTjrJ/BWZsm)

[Pritchard, J. K., Stephens, M., & Donnelly, P. (2000). Inference of population structure using multilocus genotype data. *Genetics*, *155*(2), 945–959.](http://paperpile.com/b/vNTjrJ/96Nd8)

[Pritchard, J. K., Wen, X., & Falush, D. (2010). *Documentation for structure software* (Version 2.3).](http://paperpile.com/b/vNTjrJ/YDClX) <https://web.stanford.edu/group/pritchardlab/structure_software/release_versions/v2.3.4/structure_doc.pdf>

Rivera-Colón, A. G., & Catchen, J. (2022). Population Genomics Analysis with RAD, Reprised: Stacks 2. *Marine Genomics: Methods in Molecular Biology*, *2498*, 99–149.

[Russello, M. A., Waterhouse, M. D., Etter, P. D., & Johnson, E. A. (2015). From promise to practice: pairing non-invasive sampling with genomics in conservation. *PeerJ*, *3*, e1106.](http://paperpile.com/b/vNTjrJ/Lt1wh)

[Silva-Junior, O. B., & Grattapaglia, D. (2015). Genome-wide patterns of recombination, linkage disequilibrium and nucleotide diversity from pooled resequencing and single nucleotide polymorphism genotyping unlock the evolutionary history of *Eucalyptus grandis*. *The New Phytologist*, *208*(3), 830–845.](http://paperpile.com/b/JemJQJ/M4W5)

[Sork, V. L., Cokus, S. J., Fitz-Gibbon, S. T., Zimin, A. V., Puiu, D., Garcia, J. A., Gugger, P. F., Henriquez, C. L., Zhen, Y., Lohmueller, K. E., Pellegrini, M., & Salzberg, S. L. (2022). High-quality genome and methylomes illustrate features underlying evolutionary success of oaks. *Nature Communications*, *13*(1), 2047.](http://paperpile.com/b/JemJQJ/BR2u7)

[Spence, E. S., Fant, J. B., Gailing, O., Griffith, M. P., Havens, K., Hipp, A. L., Kadav, P., Kramer, A., Thompson, P., Toppila, R., Westwood, M., Wood, J., Zumwalde, B. A., & Hoban, S. (2021). Comparing genetic diversity in three threatened oaks. *Forests, Trees and Livelihoods*, *12*(5), 561.](http://paperpile.com/b/vNTjrJ/R3dq)

[Steinkellner, H., Lexer, C., Turetschek, E., & Glössl, J. (1997). Conservation of (GA)_n_ microsatellite loci between *Quercus* species. *Molecular Ecology*, *6*(12), 1189–1194.](http://paperpile.com/b/vNTjrJ/Zm0a)

[Sullivan, A. R., Owusu, S. A., Weber, J. A., Hipp, A. L., & Gailing, O. (2016). Hybridization and divergence in multi-species oak (*Quercus*) communities. *Botanical Journal of the Linnean Society. Linnean Society of London*, *181*(1), 99–114.](http://paperpile.com/b/vNTjrJ/F5IZu)

[Toppila, R. (2012). *Ex situ conservation of oak (Quercus l.) in botanic gardens : a North American perspective* [University of Delaware].](http://paperpile.com/b/vNTjrJ/sby8P) <https://udspace.udel.edu/handle/19716/12054>

[Van Oosterhout, C., Hutchinson, W. F., Wills, D. P. M., & Shipley, P. (2004). Micro-checker: Software for identifying and correcting genotyping errors in microsatellite data. *Molecular Ecology Notes*, *4*(3), 535–538.](http://paperpile.com/b/vNTjrJ/9pi3)

[Wu, T. D., & Nacu, S. (2010). Fast and SNP-tolerant detection of complex variants and splicing in short reads. *Bioinformatics*, *26*(7), 873–881.](http://paperpile.com/b/vNTjrJ/v0iP7)

Wu, Y., Hipp, A. L., Fargo, G., Stith, N., & Ricklefs, R. E. (2023). Improving species delimitation for effective conservation: a case study in the endemic maple-leaf oak (*Quercus acerifolia*). *The New Phytologist*. https://doi.org/[10.1111/nph.18777](http://dx.doi.org/10.1111/nph.18777)

[Zumwalde, B. A., Fredlock, B., Beckman Bruns, E., Duckett, D., McCauley, R. A., Spence, E. S., & Hoban, S. (2022). Assessing ex situ genetic and ecogeographic conservation in a threatened but widespread oak after rangewide collecting effort. *Evolutionary Applications*. https://doi.org/](http://paperpile.com/b/vNTjrJ/ngMN)[10.1111/eva.13391](http://dx.doi.org/10.1111/eva.13391)

[Zumwalde, B. A., McCauley, R. A., Fullinwider, I. J., Duckett, D., Spence, E., & Hoban, S. (2021). Genetic, morphological, and environmental differentiation of an arid-adapted oak with a disjunct distribution. *Forests, Trees and Livelihoods*, *12*(4), 465.](http://paperpile.com/b/vNTjrJ/EuvA)
